# Whole-Genome Characterization of Rotavirus G9P[6] and G9P[4] Strains That Emerged after Rotavirus Vaccine Introduction in Mozambique

**DOI:** 10.3390/v16071140

**Published:** 2024-07-16

**Authors:** Benilde Munlela, Eva D. João, Amy Strydom, Adilson Fernando Loforte Bauhofer, Assucênio Chissaque, Jorfélia J. Chilaúle, Isabel L. Maurício, Celeste M. Donato, Hester G. O’Neill, Nilsa de Deus

**Affiliations:** 1 Instituto Nacional de Saúde (INS), Parcela 3943, Vila de Marracuene, Maputo 0205-02, Mozambique; evajoao29@gmail.com (E.D.J.); adilson.bauhofer@ins.gov.mz (A.F.L.B.); assucenio.chissaque@ins.gov.mz (A.C.); jorfelia.chilaule@ins.gov.mz (J.J.C.); nilsa.dedeus@ins.gov.mz (N.d.D.); 2 Instituto de Higiene e Medicina Tropical (IHMT), Universidade NOVA de Lisboa (UNL), Rua da Junqueira 100, 1349-008 Lisboa, Portugal; 3 Department of Microbiology and Biochemistry, University of the Free State, 205 Nelson Mandela Avenue, Bloemfontein 9301, South Africa; aimster.strydom@gmail.com (A.S.); oneillhg@ufs.ac.za (H.G.O.); 4 Global Health and Tropical Medicine (GHTM), Associate Laboratory in Translation and Innovation towards Global Health (LA-REAL), Instituto de Higiene e Medicina Tropical (IHMT), Universidade NOVA de Lisboa (UNL), Rua da Junqueira 100, 1349-008 Lisboa, Portugal; isabel.mauricio@ihmt.unl.pt; 5 The Peter Doherty Institute for Infection and Immunity, 792 Elizabeth Street, Melbourne, VIC 3000, Australia; celeste.donato@unimelb.edu.au; 6 Departamento de Ciências Biológicas, Universidade Eduardo Mondlane, Julius Nyerere Avenue, Maputo 3453, Mozambique

**Keywords:** rotavirus A, G9P[4], G9P[6], NSP4 E6 genotype, Mozambique

## Abstract

Mozambique introduced the Rotarix^®^ vaccine into the National Immunization Program in September 2015. Following vaccine introduction, rotavirus A (RVA) genotypes, G9P[4] and G9P[6], were detected for the first time since rotavirus surveillance programs were implemented in the country. To understand the emergence of these strains, the whole genomes of 47 ELISA RVA positive strains detected between 2015 and 2018 were characterized using an Illumina MiSeq-based sequencing pipeline. Of the 29 G9 strains characterized, 14 exhibited a typical Wa-like genome constellation and 15 a DS-1-like genome constellation. Mostly, the G9P[4] and G9P[6] strains clustered consistently for most of the genome segments, except the G- and P-genotypes. For the G9 genotype, the strains formed three different conserved clades, separated by the P type (P[4], P[6] and P[8]), suggesting different origins for this genotype. Analysis of the VP6-encoding gene revealed that seven G9P[6] strains clustered close to antelope and bovine strains. A rare E6 NSP4 genotype was detected for strain RVA/Human-wt/MOZ/HCN1595/2017/G9P[4] and a genetically distinct lineage IV or OP354-like P[8] was identified for RVA/Human-wt/MOZ/HGJM0644/2015/G9P[8] strain. These results highlight the need for genomic surveillance of RVA strains detected in Mozambique and the importance of following a One Health approach to identify and characterize potential zoonotic strains causing acute gastroenteritis in Mozambican children.

## 1. Introduction

Rotavirus remains one of the primary causative agents of gastroenteritis in children under five years of age, exerting a substantial global health burden [1,2]. It is estimated that rotavirus infections resulted in 128,500 deaths in 2016, of which 104,733 occurred in Sub-Saharan Africa [3].

Rotavirus is a member of the Sedoreoviridae family [4]. The virus has an icosahedral capsid formed by three concentric protein layers and a genome comprising 11 double-stranded ribonucleic acid (dsRNA) segments, encoding six viral and structural proteins (VP) and five or six non-structural proteins (NSP) [5]. The gene segments encoding the external capsid proteins, VP7 and VP4, of rotavirus group A (RVA) are used in a binary classification system defining G and P genotypes, respectively [2,5]. Currently, 42 G and 58 P genotypes have been described [6]. Globally, G1P[8], G2P[4], G3P[8], G4P[8], G9P[8] and G12P[8] are the most frequently detected genotype combinations, with varying prevalence observed across different countries [7,8,9,10].

A whole-genome classification system (based on nucleotide percent cut-off values) allows for the classification of all 11 RVA genes into genotype constellations designated as Gx-P[x]-Ix-Rx-Cx-Mx-Ax-Nx-Tx-Ex-Hx, with “x” indicating the number of genotypes assigned. These genotypes correspond to genome segments encoding VP7-VP4-VP6-VP1-VP2-VP3-NSP1-NSP2-NSP3-NSP4-NSP5/6 proteins [11]. To date 32I, 28R, 24C, 24M, 39A, 28N, 28T, 32E and 28H genotypes have been described [6]. The most prevalent genotype constellations in humans are the Wa-like (I1-R1-C1-M1-A1-N1-T1-E1-H1) and DS-1-like (I2-R2-C2-M2-A2-N2-T2-E2-H2) constellations. A third group known as AU-1-like (I3-R3-C3-M3-A3-N3-T3-E3-H3) is also detected in humans, albeit at a lower frequency [11,12,13].

Four rotavirus vaccines have received prequalification from the World Health Organization (WHO): Rotarix^®^, RotaTeq^®^, Rotavac^®^ and Rotasiil^®^. These vaccines have demonstrated significant efficacy in reducing diarrheal morbidity and mortality on a global scale [8]. In September 2015, Mozambique introduced the Rotarix^®^ vaccine into the National Immunization Program. Since then, the prevalence of rotavirus infection in Mozambique has decreased from 40.6% to 19.1% [14,15], the vaccine effectiveness has been estimated to be lower than what was reported in many other African countries, with an effectiveness of 30% against G1P[8] strains and 35% against non-G1P[8] strains [16].

Prior to vaccine implementation, G9P[8] and G1P[8] had been the most predominant genotypes in Mozambique [15,17]. Whole-genome analyses (WGA) of human Mozambican RVA strains before vaccine introduction have suggested genetic diversity was partially driven by reassortment events between animal and human strains [18,19]. However, post-vaccine introduction, G1P[8] became the predominant genotype nationwide [15,17], despite the use of a G1P[8]-based vaccine, although WGA of these G1P[8] strains indicated no significant mutations in epitope regions that might lead to vaccine escape, and no distinct clustering was observed between pre- and post-vaccine strains [20].

During the post-vaccine period, G9P[4], G9P[6], G3P[8] and G3P[4] have emerged as predominant genotype combinations. However, the origin of these strains, as well as their relation to the G9P[8] strains reported before vaccine introduction, remains unclear [14]. Therefore, the whole genomes of strains detected between 2015 and 2018 were determined and analyzed in the current study in order to elucidate the origin of the G9 genotype detected following vaccine introduction in Mozambique.

## 2. Materials and Methods

### 2.1. Sample Collection

Fifty-seven fecal samples collected between 2015 and 2018 as part of ongoing hospital-based sampling within the National Diarrhea Surveillance System (ViNaDia) in Mozambique [15] were selected for WGA. The samples collected in 2015 represent the pre-vaccine period and those collected in 2016–2018, the post-vaccine period (Table S1). These samples had previously tested positive for RVA by ELISA (Prospect EIA rotavirus, Basingstoke, United Kingdom) and the binary genotype combination was determined by multiplex Reverse Transcriptase (RT)-PCR [21,22,23].

### 2.2. Viral Genomic dsRNA Extraction, cDNA Library Building and Illumina MiSeq Sequencing

Total RNA was extracted from stool samples with TRI-reagent (Sigma, Darmstadt, Germany), and single-stranded RNA was precipitated with lithium chloride. The self-priming PC3-T7 loop primer (Integrated DNA Technologies, Coralville, IA, USA) was ligated to dsRNA to obtain full-length sequences. Complementary DNA (cDNA) was synthesized using the Maxima H Minus double-stranded cDNA kit (Thermo Fisher Scientific, Massachusetts, MA, USA) as previously described [19]. The cDNA was synthesized at the Next Generation Sequencing Unit at the University of the Free State in Bloemfontein, South Africa. In brief, the cDNA library was made by NEBNext Ultra RNA Library Prep Kit for Illumina v1.2 (New England Biolabs, Ipswich, MA, USA), and NEBNext Multiplex Oligos for Illumina (New England Biolabs, Ipswich, MA, USA) according to the manufacturer’s instructions and purified using Agencourt AMPure XP magnetic beads (Beckman Coulter, Brea, CA, USA). Nucleotide sequencing was performed using an Illumina MiSeq sequencer (Illumina, San Diego, CA, USA) using a MiSeq Reagent Kit V3 (Illumina, San Diego, CA, USA) [19]. 

### 2.3. Data Analysis

#### 2.3.1. Genome Assembly

A de novo assembly was performed for all samples using CLC Bio Genomics Workbench (12.0.3; Qiagen, Aarhus, Denmark); all contigs with an average coverage above 100 were identified on the Nucleotide Basic Local Alignment Search Tool (BLASTn at the National Center for Biotechnology Information—NCBI). Reference sequences were chosen based on the BLASTn results for reference mapping and extraction of consensus sequences for each segment [19]. 

#### 2.3.2. Determination of RVA Genotypes

The genotype of each of the 11 genes for each strain was determined using the Virus Pathogenic database and analysis resource (ViPR) according to the guidelines proposed by the Rotavirus Classification Working Group [6,13].

#### 2.3.3. Phylogenetic Analysis

Multiple sequence alignment of each gene was carried out using Multiple Sequence Comparison by Log Expectation (MUSCLE) alignment available in Molecular Evolutionary Genetic Analysis X (MEGA X) [24]. 

The best nucleotide substitution model, considered as having the lowest Bayesian Information Criterion, was calculated through Maximum Likelihood, as implemented in Mega X, for phylogenetic analysis and the models selected for each gene were: Tamura-3-parameter (T92+G+I) for VP7-G9, VP4-P[6], VP6-I2, VP3-M2, VP7-G1 and VP2-C1, T92+G for VP4-P[8], VP7-G3, VP7-G2, NSP1-A2 and NSP3-T2, T92+I for VP4-P[4], NSP4-E2 and NSP4-E1, T92 for NSP4-E6, NSP1-A1 and NSP2-N1, General Time Reversible (GTR+G+I) for VP1-R2, NSP2-N2 and VP3-M1, GTR+G for VP2-C1 and NSP5/6-H2, GTR+I for VP1-R1 and NSP5/6-H1. Maximum likelihood gene trees based on phylogenetic analysis of the complete ORF of the 11 genome segments for all strains were constructed using MEGA X using 1000 bootstrap replicates to estimate branch support. Pairwise distance matrix nucleotides were obtained in MEGA X using the p-distance algorithm [24]. Mozambican strains previously characterized as DS-1-like and Wa-like constellations were included in the analyses, as well as other genetically similar reference strains obtained from GenBank. The lineages were defined by previously published designations [25,26,27,28,29,30,31,32]. 

The computational tools for comparative genomics (mVISTA) online platform were used to visualize the sequence similarities of concatenated full genomes of the G9P[4] and G9P[6] exhibiting a DS-1 backbone using RVA/Human-wt/MOZ/HCN1347/2016/G9P[6] strain as reference [33]. 

#### 2.3.4. Nucleotide Sequence Accession Numbers

The nucleotide sequence data presented were deposited in GenBank under the following accession numbers: PP585813-PP586043 and PP848501-PP848786.

## 3. Results

### 3.1. Genome Constellations

Of the 47 successfully sequenced samples, 61.7% (29/4) were collected in males, 53.3% (25/47) were from children between 0–11 months old and 38.3% (18/47) were from children aged between 12–23 months old; 29.7% (14/47) were from unvaccinated children, 27.7%(13/47) from children who received two doses of the vaccine and 4.3% (2/47) were from children that received a single dose of the Rotarix^®^; 91.5% (43/47) were hospitalized due to their clinical presentation (Table S1). 

Twenty-nine strains were identified as G9 strains (Table 1). The G9P[6] (*n* = 9) and G9P[4] (*n* = 6) strains presented a typical DS-1-like constellation (-R2-C2-M2-I2-A2-N2-T2-E2-H2), except the strain RVA/Human-wt/MOZ/HCN1595/2017/G9P[4] which contained an E6 NSP4 gene (Table 1). The 14 G9P[8] strains contained a Wa-like constellation (-R1-C1-M1-I1-A1-N1-T1-E1-H1) (Table 1). The remaining strains (*n* = 18) characterized as G2P[4] (*n* = 2), G2P[6] (*n* = 7) G3P[4] (*n* = 4), G3P[8] (*n* = 3) and G1P[8] (*n* = 2) presented typical DS-1-like and Wa-like constellations (Table S2).

### 3.2. Phylogenetic Analysis

#### 3.2.1. VP7 Encoding Gene

##### G9 Genotype

**Figure 1:**
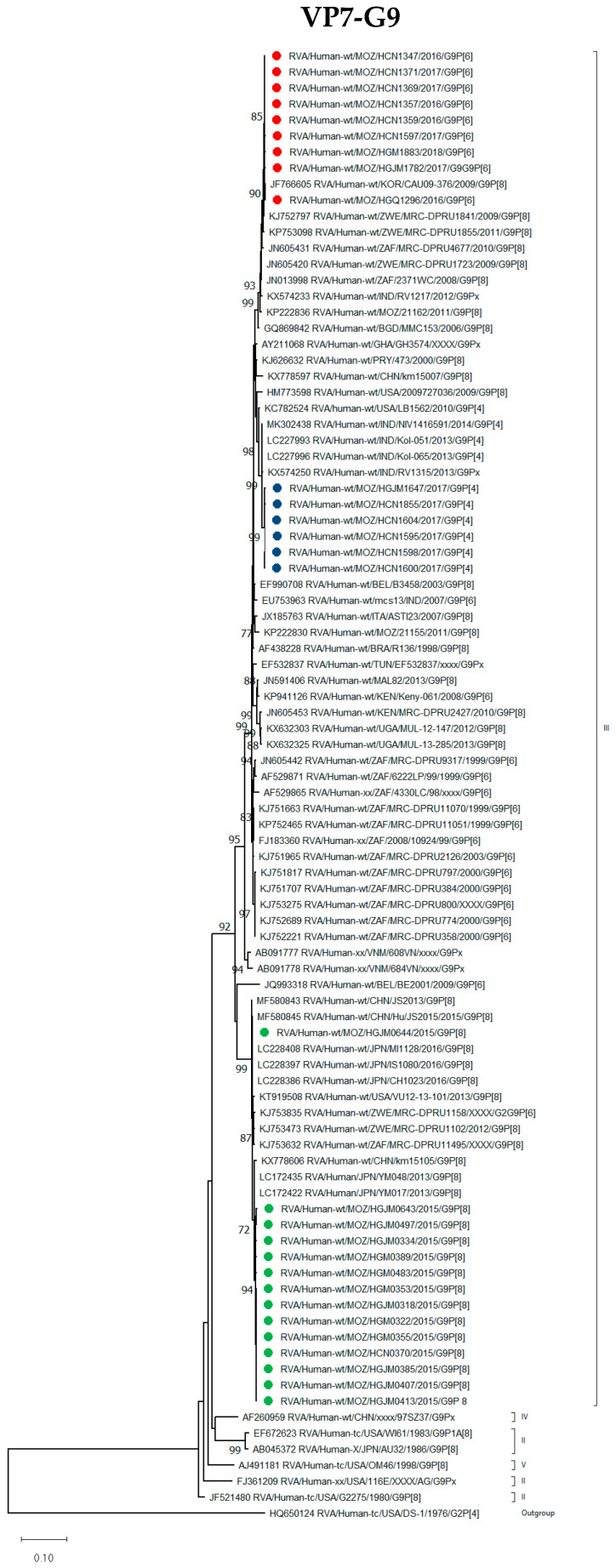
Phylogenetic tree based on the open reading frame (ORF) nucleotide sequence of the VP7-G9 encoding gene of strains circulating in Mozambique compared to global strains obtained from GenBank. The tree was constructed based on the maximum likelihood method implemented in MEGA X [24], applying Tamura-3-parameter (T92+G+I) as the model. Bootstrap values (1000 replicates) ≥70% are shown with Wa-like strain serving as an out-group. The scale bar indicates genetic distance expressed as the number of nucleotide substitutions per site. G9P[4] Mozambican strains are indicated by blue circles, G9P[6] by red circles and G9P[8] by green circles.

The G9 strains, in combination with P[4], P[6] and P[8], formed three different conserved clades within lineage III (Figure 1). The G9P[4] strains from the post-vaccine period grouped with G9P[4] strains from India detected in 2013 and 2014. The nine G9P[6] strains detected in the post-vaccine introduction period clustered with strains from Zimbabwe (2009 and 2011) and South Africa (2008 and 2010), but in combination with P[8] and no other G9P[6] strains previously detected in Southern Africa. The 13 G9P[8] strains, detected before the introduction of the rotavirus vaccine, clustered together with G9P[8] strains from Japan that circulated in 2013. Seven of the nine G9P[6] strains were detected in unvaccinated children. One G9P[8] strain (RVA/Human-wt/MOZ/HGJM0644/2015/G9P[8]), clustered distinctly from other Mozambican strains and shared 99.1–99.2% nucleotide (nt) identity and 98.8–99.1% amino acid (aa) identity with the other 12 G9P[8] Mozambican strains (Table S3). This Mozambican strain clustered with a Japanese strain from 2016 (Figure 1). 

##### G3, G2 and G1 Genotypes

The seven G3 strains clustered in lineage III (Figure S1) and shared an identity of 98.7–99.9% (98.2–100%) nt (aa). The three G3P[8] detected in vaccinated children were closely related to G3P[8] strains from Japan and Kenya detected in 2017 and 2019 (RVA/Human-wt/JPN/Tokyo17-21/2017/G3P[8] and RVA/Human-wt/KEN/KLF0929/2019/G3P[8]). The four G3P[4] were closely related to RVA/Human-wt/PAK/PAK663/2016/G3P[4] from Pakistan (Figure S1). Two Mozambican G3P[8] strains previously described from a rural site in Mozambique (RVA/Human-wt/MOZ/MAN1811450.8/2021/G3P[8] and RVA/Human-wt/MOZ/MAN1811463.8/2021/G3P[8]) clustered between the G3P[8] and G3P[4] study strains and shared an identity of 97.5–98.5% (98.4–99.0%) nt (aa). 

All G2 strains from pre (2015) and post-vaccine introduction (2016) grouped together in lineage IV sub lineage a-3 III (Figure S1). These strains were closely related to G2P[4] strains from Southern Africa, including Malawi (RVA/Human-wt/MWI/BID115/2012/G2P[4]) as well as three Mozambican G2P[4] strains that circulated in 2013 (Figure S1).

The G1P[8] strains, RVA/Human-wt/MOZ/HGJM0408/2015/G1P[8] (pre-vaccine period) and RVA/Human-wt/MOZ/HCN1556/2017/G1P[8] (detected in a vaccinated child), clustered in a highly conserved clade of previously characterized G1P[8] Mozambican strains in lineage II (Figure S1).

#### 3.2.2. VP4 Encoding Gene

##### P[4], P[6] and P[8] Genotypes

**Figure 2:**
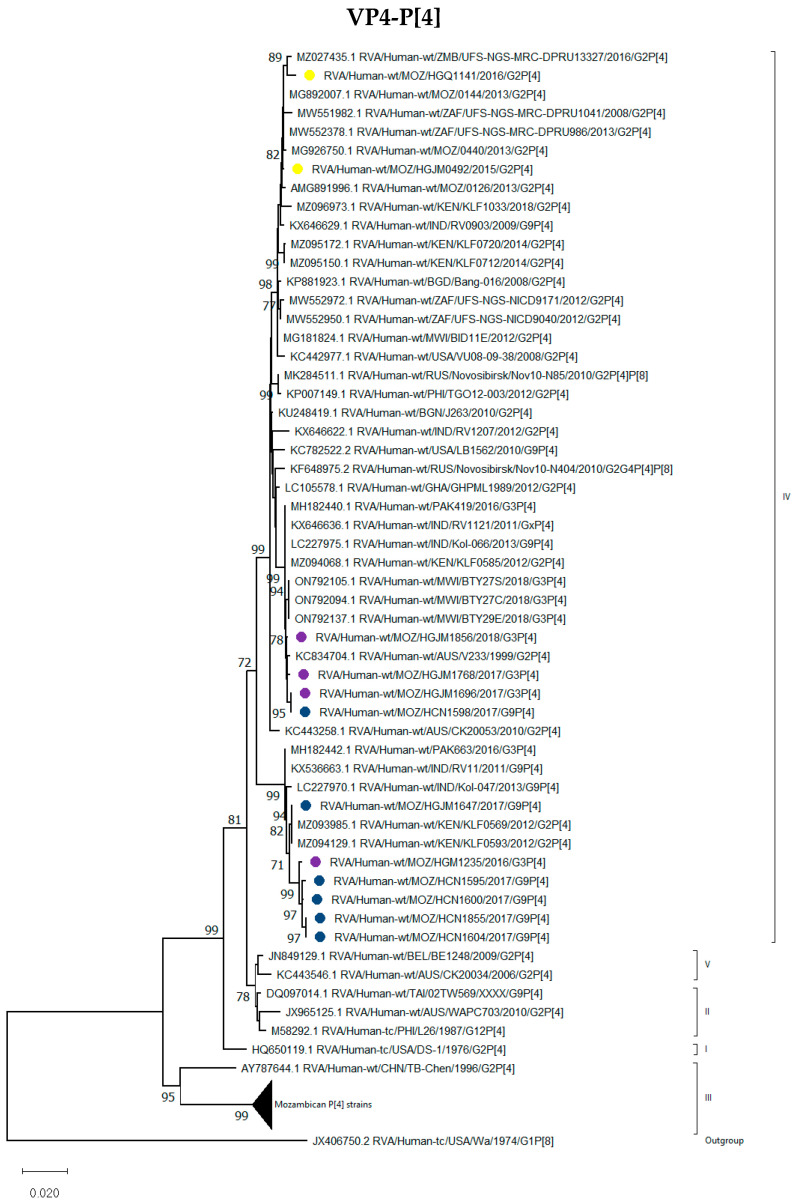
Phylogenetic tree based on the open reading frame (ORF) nucleotide sequence of the VP4-P[4] encoding gene of strains circulating in Mozambique compared to global strains obtained from GenBank. The tree was constructed based on the maximum likelihood method implemented in MEGA X [24], applying Tamura-3-parameter (T92+I) as the model. Bootstrap values (1000 replicates) ≥70% are shown with Wa-like strain serving as an out-group. The scale bar indicates genetic distance expressed as the number of nucleotide substitutions per site. G9P[4] Mozambican strains are indicated by blue circles, G3P[4] by purple circles and G2P[4] by yellow circles.

All P[4] genotypes detected in the study were compared to rotavirus sequences representing the five lineages of the P[4] encoding gene, and the results showed that all sequences were grouped in lineage IV. It was observed that five of the G9P[4] and one G3P[4] strains grouped with the G2P[4] strains from Kenya that circulated in 2012 (RVA/Human-wt/KEN/KLF0569/2012/G2P[4] and RVA/Human-wt/KEN/KLF0593/2012/G2P[4]) and G9P[4] strains from India reported in 2011 (RVA/Human-wt/IND/RV11/2011/G9P[4]) and 2013 (RVA/Human-wt/IND/Kol-047/2013/G9P[4]) and a G3P[4] strain from Pakistan (Figure 2). Interestingly, the remaining G9P[4] strain, RVA/Human-wt/MOZ/HCN1598/2017/G9P[4], detected in northern Mozambique, clustered with G3P[4] Mozambican strains detected in southern Mozambique and shared a % nt (aa) identity of 99.7–100% (99.6–100%) (Figure 2).

**Figure 3:**
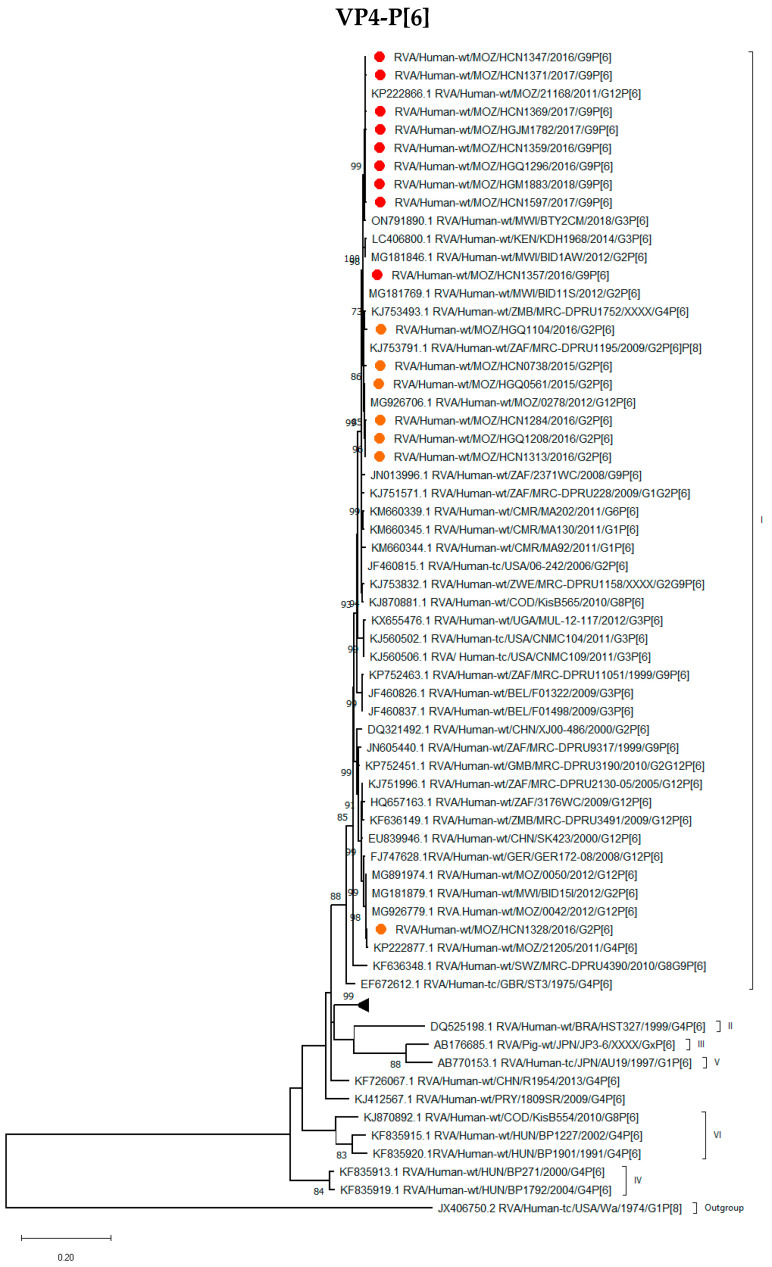
Phylogenetic tree based on the ORF nucleotide sequence of the VP4-P[6] encoding gene of strains circulating in Mozambique compared to global strains obtained from GenBank. The tree was constructed based on the maximum likelihood method implemented in MEGA X [24], applying T92+G+I as the model. Bootstrap values (1000 replicates) ≥70% are shown with Wa-like strain serving as an out-group. The scale bar indicates genetic distance expressed as the number of nucleotide substitutions per site. G9P[6] Mozambican strains are indicated by red circles and G2P[6] by brown circles.

All nine P[6] strains in combination with G9 clustered closely together in lineage I with the G2P[6] strains described in this study and with other G12P[6] and G2P[6] strains previously described from Mozambique and other Southern and Eastern African countries. The exception was RVA/Human-wt/MOZ/HCN1328/2016/G2P[6] that grouped separately from the rest of the study strains with G12P[6] Mozambican strains detected in 2012 (Figure 3).

**Figure 4:**
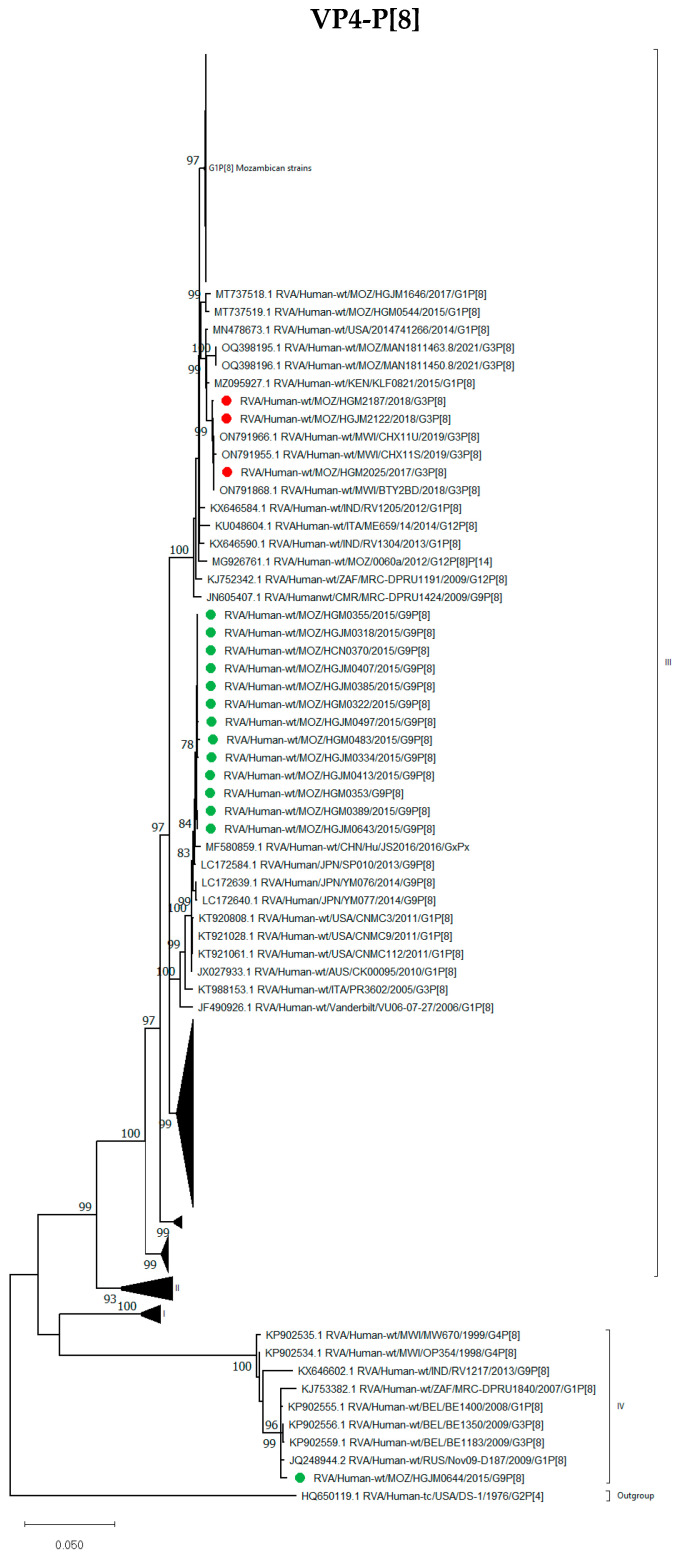
Phylogenetic tree based on the ORF nucleotide sequence of the VP4-encoding genes (P[8]) of strains circulating in Mozambique compared to global strains obtained from GenBank. The tree was constructed based on the maximum likelihood method implemented in MEGA X [24], applying T92+G as the model. Bootstrap values (1000 replicates) ≥70% are shown with DS-1-like strains serving as an out-group. The scale bar indicates genetic distance expressed as the number of nucleotide substitutions per site. G3P[8] Mozambican strains are indicated by red circles and G9P[8] by green circles.

Thirteen G9P[8] strains from 2015 (pre-vaccine period) formed a conserved clade in lineage III and grouped close to G9P[8] strains from Japan and China, as well as G1P[8] strains from Australia and the USA. The three G3P[8] strains were also grouped in lineage III, although in a separate cluster from the G9P[8] strains. The two G1P[8] (RVA/Human-wt/MOZ/HGJM0408/2015/G1P[8] and RVA/Human-wt/MOZ/HCN1556/2017/G1P[8]) strains formed clusters with Mozambican G1P[8] strains reported between 2012 and 2017. One G9P[8] strain, RVA/Human-wt/MOZ/HGJM0644/2015/G9P[8], clustered separately in the rare lineage IV with G1P[8] and G3P[8] strains from Belgium and Russia (2008 and 2009, respectively) (Figure 4).

#### 3.2.3. VP1–VP3 and VP6 Encoding Genes

##### VP1–VP3

For genotypes R2 (VP1) and C2 (VP2), the G9P[6] and G9P[4] strains grouped into the same cluster with G2P[6] and G3P[4] study strains, and was closely related to Mozambican G2P[4] strains that circulated in 2013. An exception was observed for the C2 genotype, where the G3P[4] strains formed a separate cluster with one of the G9P[6] strains, (RVA/Human-wt/MOZ/HGM1782/2017/G9P[6]) and G3P[4] strains from Pakistan which circulated in 2016 (Figure S1).

Similar groupings were observed for the M2 (VP3) genotype. where the G9P[4] and G9P[6] clustered in the same major clade with the G2P[6] study strains and G2P[4] that circulated in 2013 and 2015. The exception was two study strains, (RVA/Human-wt/MOZ/HGM1782/2017/G9P[6] and RVA/Human-wt/MOZ/HCN1595/2017/G9P[4]), that clustered separately with the study G3P[4] strains from Mozambique (Figure S1).

##### VP6

The I2 genotype of G9P[4] strains grouped into a conserved cluster in lineage V and were closely related to Malawian (RVA/Human-wt/MWI/BID1JK/2013/G2P[4] and RVA/Human-wt/MWI/BID2DE/2013/G1P[8]) and Indian (RVA/Human-wt/IND/CMC00024/2012/G2Px) strains. 

**Figure 5:**
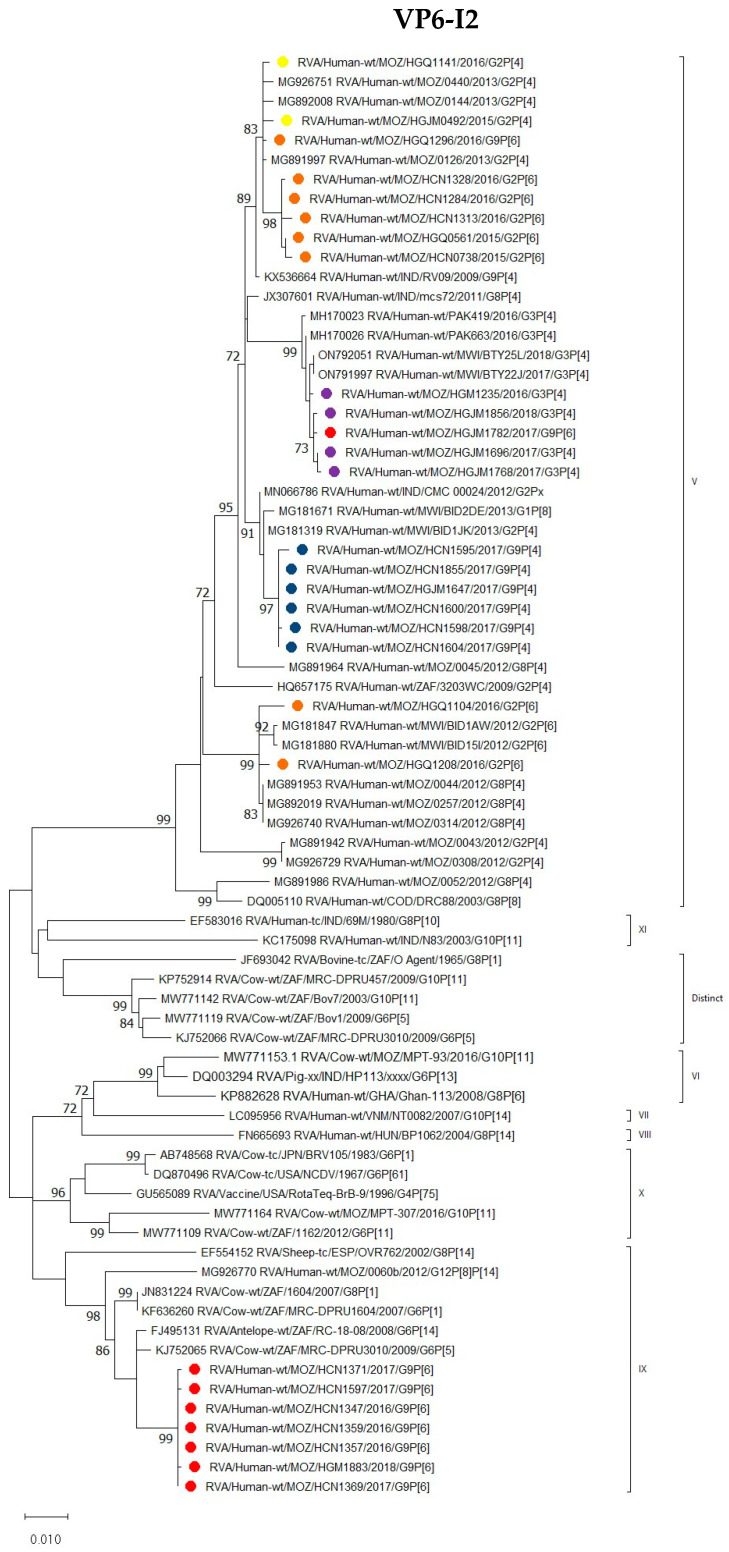
Phylogenetic tree based on the ORF nucleotide sequence of the VP6-encoding I2 genes of strains circulating in Mozambique compared to global strains obtained from GenBank. The best-fit nucleotide substitution model T92+G+I was used. The tree was constructed based on the maximum likelihood method implemented in MEGA X [24]. Bootstrap values (1000 replicates) ≥70% are shown with Wa-like as out-group. The scale bar indicates genetic distance expressed as the number of nucleotide substitutions per site. G9P[4] Mozambican strains are indicated by blue circles, G9P[6] by red circles, G3P[4] by purple circles, G2P[4] by yellow circles and G2P[6] by brown circles.

Seven of the G9P[6] strains that circulated between 2016 and 2018 shared an nt (aa) identity of 99.9% (100%). These strains grouped into lineage IX in a cluster that contained animal strains, such as antelope (RVA/Antelope-wt/ZAF/RC-18-08/G6P[14]), with which it shared nt (aa) identity of 98.8% (100%). Also included in this cluster were bovine strains RVA/Cow-wt/ZAF/1604/2007/G8P[1], RVA/Cow-wt/ZAF/MRC-DPRU1604/2007/G6P[1] and RVA/Cow-wt/ZAF/MRC-DPRU3010/2009/G6P[5] with an average nt (aa) identity of 98.3% (99.6%). A VP6-encoding sequence of a human mixed infection strain (RVA/Human-wt/MOZ/0060b/2012/G12P[8]P[14]), previously reported to be of animal origin, also clustered in lineage IX, whereas bovine strains from Mozambique (RVA/Cow-wt/MOZ/MPT-93/2016/G10P[11] and RVA/Cow-wt/MOZ/MPT-307/2016/G10P[11]) clustered in lineages VI and X, respectively, with an average nt (aa) identity of 94.8% (99.6%) to the study strains (Figure 5).

Two G9P[6] strains were grouped in separated clusters; one strain (RVA/Human-wt/MOZ/HGQ1296/2016/G9P[6]) shared an nt identity of only 93.8% with the other seven G9P[6] strains, and formed a cluster with G2P[6] and G2P[4] study strains. Study strain RVA/Human-wt/MOZ/HGJM1782/2017/G9P[6] detected in a vaccinated child shared an average nt identity of 92.5%, with the rest of the G9P[6] strains and grouped with G3P[4] Mozambican study strains (detected in unvaccinated children) and with G3P[4] from Pakistan as in the other segments (Figure 5).

#### 3.2.4. NSP1-NSP5/NSP6 Encoding Genes

The conserved clade observed in the VPs encoding genes, formed by eight G9P[6] and five G9P[4], clustered together with G2P[4] strains from Mozambique that circulated in 2013, 2015 and 2016 for the NSP-encoding genes. The RVA/Human-wt/MOZ/HCN1595/2017/G9P[4], RVA/Human-wt/MOZ/HGM1782/2017/G9P[6] and RVA/Human-wt/MOZ/HGQ1296/2016/G9P[6] strains, continued to show varied clustering patterns across the trees. In the NSP1-encoding gene tree RVA/Human-wt/MOZ/HCN1595/2017/G9P[4] clustered separately with a G9P[4] strains from India, RVA/Human-wt/MOZ/HGM1782/2017/G9P[6] grouped with the four G3P[4] Mozambican strains and RVA/Human-wt/MOZ/HGQ1296/2016/G9P[6] was closely related to the major clade of the G9P[6] and G9P[4] study strains (Figure S1).

In the NSP2, NSP3 and NSP5 trees, only the RVA/Human-wt/MOZ/HCN1595/2017/G9P[4] strain diverged from the group and clustered separately from the major clade. This strain was closely related to G1P[8], G2P[6] and G9P[4] Mozambican study and Asian strains across the trees. The major clade was related to G2P[4] and G2P[6] strains from Mozambique and Kenya (Figure S1).

The E2 NSP4 genotypes were identified in all 15 strains with the exception of RVA/Human-wt/MOZ/HCN1595/2017/G9P[4] strain (Table 1). Five G9P[4] and eight G9P[6] clustered with four G8P[4] and G2P[4] Mozambican strains from 2012 (Figure S1). The rare E6 genotype partial ORF of NSP4 encoding RVA/Human-wt/MOZ/HCN1595/2017/G9P[4] strain clustered with a G9P[4] Indian strain RVA/Human-wt/IND/RV0903/2009/G9P[4] detected in 2009 with nt (aa) identity of 99.6 (98.7)% (Figure S1). This study strain was detected in a vaccinated 8-month-old male child from Nampula province, northern Mozambique (Table S1). 

### 3.3. Mvista Analyses

To further study the reassortment events suggested by the phylogenetic analysis, the concatenated DS-1-like genetic backbone (VP6-VP1-VP2-VP3-NSP1-NSP2-NSP3-NSP4 NSP5/6) of the G9P[4] and G9P[6] where aligned by Mvista, using the RVA/Human-wt/MOZ/HCN1347/2016/G9P[6] strain as reference. The results showed that all nine genes of the Mozambican strain exhibited a relatively high degree of conservation, with the exception of the VP6-encoding gene. The VP6 of the G9P[4] strains was conserved in all strains, but showed differences when compared to G9P[6] strains except RVA/Human-wt/MOZ/HGQ1296/2016/G9P[6] and RVA/human-wt/MOZ/HGM1782/2017/G9P[6]. 

**Figure 6:**
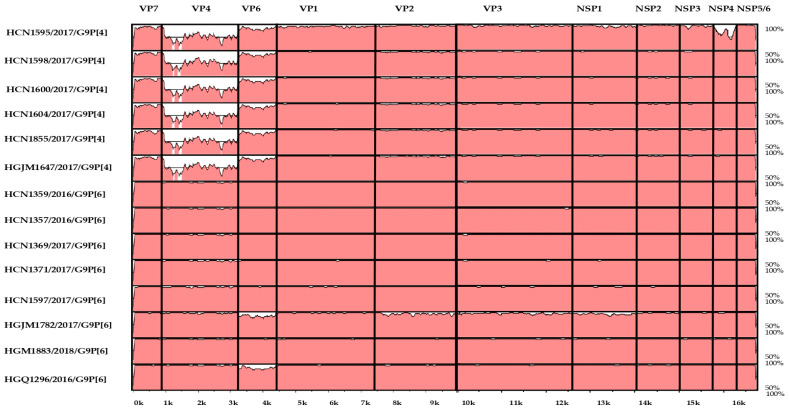
Nucleotide sequence similarities of the G9P[4] and G9P[6] concatenated genomes using the RVA/Human-wt/MOZ/HCN1347/2016/G9P[6] strain as reference. The name of the Mozambican strains is indicated on the left, and the positions of the 11 genes are indicated at the top. The scale indicates the distance in kb.

In addition, the VP1–VP3, NSP1, NSP3 and NSP4 encoding genes of the RVA/Human-wt/MOZ/HCN1595/2017/G9P[4] and RVA/Human-wt/MOZ/HGM1782/2017/G9P[6] strains showed a different pattern compared to the other study strains, which likely derived through reassortment events (Figure 6).

## 4. Discussion

In the present study, WGS was performed for 47 strains with specific focus on strains identified as G9P[6], G9P[4] and G9P[8], obtained from Mozambican children with gastroenteritis between 2015 and 2018. 

The report of G9P[8] strains as the most predominant genotype in the country before vaccine introduction and the first detection of G9P[4] and G9P[6] genotypes after the vaccine introduction in Mozambique [15], led to the need to monitor changes in strain diversity at gene level. Phylogenetic analysis of the G9 Mozambican strains showed that G9P[8] strains had a Wa-like constellation and the G9P[6] and G9P[4] strains, a DS-1-like constellation with the exception of strain RVA/Human-wt/MOZ/HCN1595/2017/G9P[4] which contained an E6 NSP4 gene. These results highlight the increase of the DS-1 backbone strains after vaccine introduction following the global trend at the time that the strains were detected [10,34,35,36,37].

The 13 G9P[8] strains, detected before the introduction of the rotavirus vaccine, clustered together in lineage III. The P[8] lineage III is described as the most common globally [25].

Strain RVA/Human-wt/MOZ/HGJM0644/2015/G9P[8] clustered in a genetically distinct lineage known as lineage IV or OP354-like P[8]. This lineage has been reported in different parts of Europe, Africa and Asia [25,27]. In fact, the Mozambican P[8] lineage IV strain in this study was detected in a 9-month-old female child and could not be detected by RT-PCR, being non-typable for the P genotype. The same observation was reported in Ghana, where 10.4% of non-typeable rotavirus VP4 genes were identified as rare OP354-like P[8] by full-genome sequencing of this rare strain [27,38].

The phylogenetic analysis of the G9 Mozambican strains showed a common pattern between the G9P[4] and G9P[6] strains since most of them clustered together for all gene segments. These results indicate a common ancestral strain with the exception of the RVA/Human-wt/MOZ/HCN1595/2017/G9P[4], RVA/Human-wt/MOZ/HGQ1296/2016/G9P[6], RVA/Human-wt/MOZ/HGM1782/2017/G9P[6] and RVA/Human-wt/MOZ/HCN1598/2017/G9P[4] strains, which clustered distinctly from the larger clade in some segments.

Interestingly, the RVA/Human-wt/MOZ/HGM1782/2017/G9P[6] strain clustered with the G3P[4] strains in segments encoding VP6, VP2, VP3 and NSP1. HGM1782 was detected in the Maputo province, in the same geographic location as the G3P[4] strains, which could explain the similar clustering for the four genome segments. The RVA/Human-wt/MOZ/HCN1598/2017/G9P[4] strain from the northern region of the country, clustered with G3P[4] Mozambican strains from the southern region of Mozambique, for the VP4 encoding gene. These results were confirmed in the Mvista analysis, where these strains were diverse in relation to the others, thus suggesting that they originated through reassortment events. Most of the G9P[4] study strains were detected in vaccinated children, unlike the G9P[6] study strains, which were mostly obtained from unvaccinated children. Regardless, no different clusters were observed between strains detected in vaccinated and unvaccinated children for both genotypes.

The segment encoding for VP6 had the most distinct clustering pattern among the strains. In this segment, seven G9P[6] strains had a higher genetic identity and formed a cluster with animal strains from South Africa and a human Mozambican strain, which had previously been reported as a mixed infection of animal origin [18]. The antelope strain, which was similar to the G9P[6] study strains, was highlighted as having a common origin with the G6P[14] human strains in some segments, excluding the VP6 encoding gene [39]. Three of these Mozambican strains were isolated from children who had contact with animals, including horses, sheep and cats. These results suggest that interspecies transmission occurred. 

Unusual G9P[4] RVA strains have been reported in several countries, such as India, Italy, Japan, Benin and Ghana, during pre- and post-vaccine introduction periods [31,32,34,35,36,37,40,41,42]. It has been hypothesized that reassortment events among contemporary human rotavirus strains generated these unusual G9P[4] strains [43]. The E6 NSP4 genotype was first identified in 2000 in India, and the analysis showed that the most common recent ancestor was likely to have been around 1981 in Asia [31]. A recent study in Benin and Ghana described this genotype in Africa for the first time [34,40]. The G9P[4] E6-NSP4 Mozambican strain was identified in the same period as the Benin and Ghanian strains; however, it was related to strains from India. Only one of the characterized strains exhibited the E6 genotype, which indicates a single inter-genotype reassortment event or a sporadic event in Mozambique [37]. Further analyses are necessary to compare diarrhea severity and changes in the NSP4 protein; for example, an evaluation of the E6 genotype in an animal model could possibly determine if the observed chances are linked to increased pathogenesis of the genotype. In addition, continued genome surveillance is needed to monitor the occurrence of such unusual strains and the possibility of becoming predominant in the country causing severe acute gastroenteritis in children.

Limitations of the study include the short period analyzed (2015–2018) and the inability to calculate the Vesikari score to compare the severity of strains from the post-vaccine period. There is a need to expand the whole-genome analysis to strains detected after 2018 to fully comprehend the genetic diversity of rotavirus strains detected after vaccine introduction.

## 5. Conclusions

The study results indicate that G9P[4] and G9P[6] strains exhibited a DS-1-like genetic constellation after Rotarix^®^ vaccine introduction in Mozambique. The occurrence of unusual genotypes and close relationship with animal strains, suggesting inter-genotype reassortment and interspecies events, highlight the need for continuous genomic surveillance of RVA strains detected in Mozambique and the importance of following a One Health approach to identify and characterize potential zoonotic strains causing acute gastroenteritis in children. 

## Figures and Tables

**Table 1:** Genotype constellation of Mozambican G9P[4], G9P[6] and G9P[8] strains.

Strain Name	VP7	VP4	VP6	VP1	VP2	VP3	NSP1	NSP2	NSP3	NSP4	NSP5/6
Segment	9	4	6	1	2	3	5	8	7	10	11
**Wa-like**	G1	P[8]	I1	R1	C1	M1	A1	N1	T1	E1	H1
**DS1-like**	G2	P[4]	I2	R2	C2	M2	A2	N2	T2	E2	H2
RVA/Human-wt/MOZ/HCN1357/2016/G9P[6]	G9	P[6]	I2	R2	C2	M2	A2	N2	T2	E2	H2
RVA/Human-wt/MOZ/HCN1359/2016/G9P[6]	G9	P[6]	I2	R2	C2	M2	A2	N2	T2	E2	H2
RVA/Human-wt/MOZ/HCN1369/2017/G9P[6]	G9	P[6]	I2	R2	C2	M2	A2	N2	T2	E2	H2
RVA/Human-wt/MOZ/HCN1371/2017/G9P[6]	G9	P[6]	I2	R2	C2	M2	A2	N2	T2	E2	H2
RVA/Human-wt/MOZ/HCN1597/2017/G9P[6]	G9	P[6]	I2	R2	C2	M2	A2	N2	T2	E2	H2
RVA/Human-wt/MOZ/HGJM1782/2017/G9P[6]	G9	P[6]	I2	R2	C2	M2	A2	N2	T2	E2	H2
RVA/Human-wt/MOZ/HGQ1296/2016/G9P[6]	G9	P[6]	I2	R2	C2	M2	A2	N2	T2	E2	H2
RVA/Human-wt/MOZ/HCN1347/2016/G9P[6]	G9	P[6]	I2	R2	C2	M2	A2	N2	T2	E2	H2
RVA/Human-wt/MOZ/HGM1883/2018/G9P[6]	G9	P[6]	I2	R2	C2	M2	A2	N2	T2	E2	H2
RVA/Human-wt/MOZ/HCN1595/2017/G9P[4]	G9	P[4]	I2	R2	C2	M2	A2	N2	T2	E6	H2
RVA/Human-wt/MOZ/HCN1855/2017/G9P[4]	G9	P[4]	I2	R2	C2	M2	A2	N2	T2	E2	H2
RVA/Human-wt/MOZ/HCN1598/2017/G9P[4]	G9	P[4]	I2	R2	C2	M2	A2	N2	T2	E2	H2
RVA/Human-wt/MOZ/HGJM1647/2017/G9P[4]	G9	P[4]	I2	R2	C2	M2	A2	N2	T2	E2	H2
RVA/Human-wt/MOZ/HCN1600/2017/G9P[4]	G9	P[4]	I2	R2	C2	M2	A2	N2	T2	E2	H2
RVA/Human-wt/MOZ/HCN1604/2017/G9P[4]	G9	P[4]	I2	R2	C2	M2	A2	N2	T2	E2	H2
RVA/Human-wt/MOZ/HGM483/2015/G9P[8]	G9	P[8]	I1	R1	C1	M1	A1	N1	T1	E1	H1
RVA/Human-wt/MOZ/HGJM0318/2015/G9P[8]	G9	P[8]	I1	R1	C1	M1	A1	N1	T1	E1	H1
RVA/Human-wt/MOZ/HGJM0334/2015/G9P[8]	G9	P[8]	I1	R1	C1	M1	A1	N1	T1	E1	H1
RVA/Human-wt/MOZ/HGM0355/2015/G9P[8]	G9	P[8]	I1	R1	C1	M1	A1	N1	T1	E1	H1
RVA/Human-wt/MOZ/HCN0370/2015/G9P[8]	G9	P[8]	I1	R1	C1	M1	A1	N1	T1	E1	H1
RVA/Human-wt/MOZ/HGJM0407/2015/G9P[8]	G9	P[8]	I1	R1	C1	M1	A1	N1	T1	E1	H1
RVA/Human-wt/MOZ/HGJM0385/2015/G9P[8]	G9	P[8]	I1	R1	C1	M1	A1	N1	T1	E1	H1
RVA/Human-wt/MOZ/HGM0322/2015/G9P[8]	G9	P[8]	I1	R1	C1	M1	A1	N1	T1	E1	H1
RVA/Human-wt/MOZ/HGJM0497/2015/G9P[8]	G9	P[8]	I1	R1	C1	M1	A1	N1	T1	E1	H1
RVA/Human-wt/MOZ/HGJM0413/2015/G9P[8]	G9	P[8]	I1	R1	C1	M1	A1	N1	T1	E1	H1
RVA/Human-wt/MOZ/HGM0353/2015/G9P[8]	G9	P[8]	I1	R1	C1	M1	A1	N1	T1	E1	H1
RVA/Human-wt/MOZ/HGM0389/2015/G9P[8]	G9	P[8]	I1	R1	C1	M1	A1	N1	T1	E1	H1
RVA/Human-wt/MOZ/HGJM0643/2015/G9P[8]	G9	P[8]	I1	R1	C1	M1	A1	N1	T1	E1	H1
RVA/Human-wt/MOZ/HGJM0644/2015/G9P[8]	G9	P[8]	I1	R1	C1	M1	A1	N1	T1	E1	H1

Wa-like and DS-1-like genotypes constellation are shown in green and red respectively. The G9 and E6 genotype are shown in white and P[6] genotype is shown in blue [12].

## Data Availability

The data are available upon request from the corresponding author.

## Supplementary Materials

The following supporting information can be downloaded at: https://www.mdpi.com/article/10.3390/v16071140/s1, Figure S1: Phylogenetic trees based on the ORF nucleotide sequence of the Mozambican strains compared to global strains obtained from GenBank; Table S1: Vaccination status of the Children infected by G2P[6], G2P[6], G3P[4], G3P[8] and G1P[8] Mozambican strains; Table S2: Genotype constellation of Mozambican G2P[6], G2P[6], G3P[4], G3P[8] and G1P[8] strains; Table S3: Nucleotide and amino acid identities of the Mozambicans trains.
