# Supplementary material for: Whole-Genome Characterization of Rotavirus G9P[6] and G9P[4] Strains That Emerged after Rotavirus Vaccine Introduction in Mozambique
Source: Viruses. 2024 Jul 16;16(7):1140. doi: 10.3390/v16071140 (PMC11281483; doi:10.3390/v16071140)
Supplement: Supplementary file 1 [file viruses-16-01140-s001.zip › Figure S1.pdf]

VP7-G2

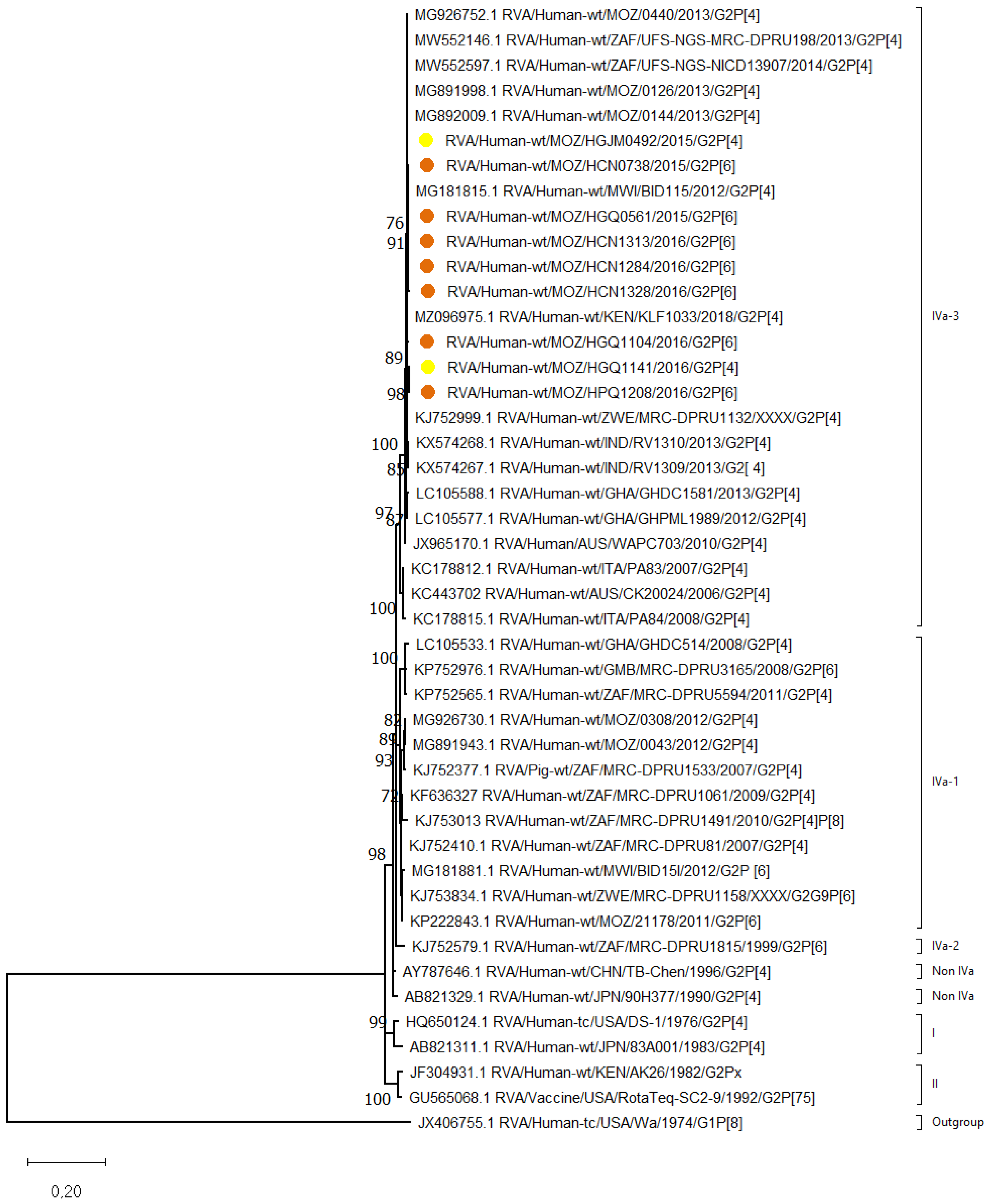

Figure S1: Phylogenetic trees based on the ORF nucleotide sequences of the DS1-S (VP7-G2, VP7-G3, VP1-R2, VP2-C2, VP3-M2, NSP1-A2, NSP2-N2, NSP3-T2, NSP4-E2 and NSP5/6-H2) and (VP7-G1, VP6-I1, VP1-R1, VP2-C1, VP3-M1, NSP1-A1, NSP2-N1, NSP3-T1, NSP4-E1, and NSP5/6-H1) genes of circulating Mozambican and global strains obtained from GenBank. The trees were constructed based on the maximum likelihood method implemented in MEGA X [29], using the best-fit nucleotide substitution model Tamura-3-parameter (T92+G+I) for VP6-I2, VP3- M2, VP7-G1 and VP2-C1, T92+G for VP7-G3, VP7-G2, NSP1-A2 and NSP3-T2, T92+I for , NSP4-E2 and NSP4-E1, T92 for NSP4-E6, NSP1-A1 and NSP2-N1, General Time Reversible (GTR+G+I) for VP1-R2, NSP2-N2 and VP3-M1, GTR+G for VP2-C1 and NSP5/6-H2, GTR+I for VP1-R1 and NSP5/6-H1. Bootstrap values (1000 replicates) 70%. Scale bar indicates genetic distance expressed as the number of nucleotide substitutions per site. G9P[4] strains are indicated by blue, G9P[6] by red, G3P[4] by purple, G3P[8] by red, G2P[4] by yellow, G2P[6] by brown and G1P[8] by black circles.

VP7-G3

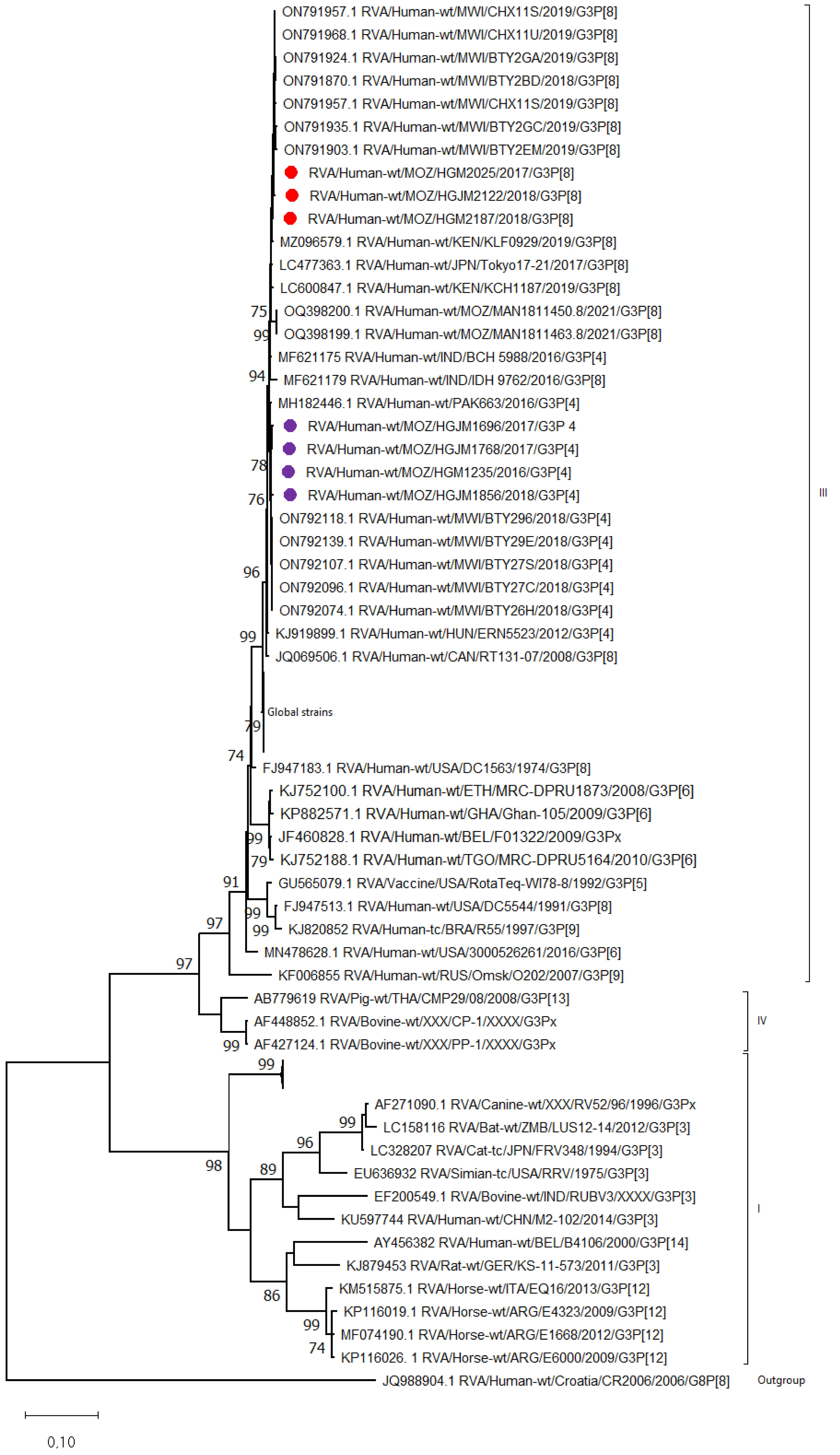
ON791957.1 RVA/Human-wt/MWI/CHX11S/2019/G3P[8]
ON791968.1 RVA/Human-wt/MWI/CHX11U/2019/G3P[8]
ON791924.1 RVA/Human-wt/MWI/BTY2GA/2019/G3P[8]
ON791870.1 RVA/Human-wt/MWI/BTY2BD/2018/G3P[8]
ON791957.1 RVA/Human-wt/MWI/CHX11S/2019/G3P[8]
ON791935.1 RVA/Human-wt/MWI/BTY2GC/2019/G3P[8]
ON791903.1 RVA/Human-wt/MWI/BTY2EM/2019/G3P[8]
RVA/Human-wt/MOZ/HGM2025/2017/G3P[8]
RVA/Human-wt/MOZ/HGJM2122/2018/G3P[8]
RVA/Human-wt/MOZ/HGM2187/2018/G3P[8]
MZ096579.1 RVA/Human-wt/KEN/KLF0929/2019/G3P[8]
LC477363.1 RVA/Human-wt/JPN/Tokyo17-21/2017/G3P[8]
LC600847.1 RVA/Human-wt/KEN/KCH1187/2019/G3P[8]
OQ398200.1 RVA/Human-wt/MOZ/MAN1811450.8/2021/G3P[8]
OQ398199.1 RVA/Human-wt/MOZ/MAN1811463.8/2021/G3P[8]
MF621175 RVA/Human-wt/IND/BCH 5988/2016/G3P[4]
MF621179 RVA/Human-wt/IND/IDH 9762/2016/G3P[8]
MH182446.1 RVA/Human-wt/PAK663/2016/G3P[4]
RVA/Human-wt/MOZ/HGJM1696/2017/G3P 4
RVA/Human-wt/MOZ/HGJM1768/2017/G3P[4]
RVA/Human-wt/MOZ/HGM1235/2016/G3P[4]
RVA/Human-wt/MOZ/HGJM1856/2018/G3P[4]
ON792118.1 RVA/Human-wt/MWI/BTY296/2018/G3P[4]
ON792139.1 RVA/Human-wt/MWI/BTY29E/2018/G3P[4]
ON792107.1 RVA/Human-wt/MWI/BTY27S/2018/G3P[4]
ON792096.1 RVA/Human-wt/MWI/BTY27C/2018/G3P[4]
ON792074.1 RVA/Human-wt/MWI/BTY26H/2018/G3P[4]
KJ919899.1 RVA/Human-wt/HUN/ERN5523/2012/G3P[4]
JQ069506.1 RVA/Human-wt/CAN/RT131-07/2008/G3P[8]
Global strains
FJ947183.1 RVA/Human-wt/USA/DC1563/1974/G3P[8]
KJ752100.1 RVA/Human-wt/ETH/MRC-DPRU1873/2008/G3P[6]
KP882571.1 RVA/Human-wt/GHA/Ghan-105/2009/G3P[6]
JF460828.1 RVA/Human-wt/BEL/F01322/2009/G3Px
KJ752188.1 RVA/Human-wt/TGO/MRC-DPRU5164/2010/G3P[6]
GU565079.1 RVA/Vaccine/USA/RotaTeq-WI78-8/1992/G3P[5]
FJ947513.1 RVA/Human-wt/USA/DC5544/1991/G3P[8]
KJ820852 RVA/Human-tc/BRA/R55/1997/G3P[9]
MN478628.1 RVA/Human-wt/USA/3000526261/2016/G3P[6]
KF006855 RVA/Human-wt/RUS/Omsk/O202/2007/G3P[9]
AB779619 RVA/Pig-wt/THA/CMP29/08/2008/G3P[13]
AF448852.1 RVA/Bovine-wt/XXX/CP-1/XXXX/G3Px
AF427124.1 RVA/Bovine-wt/XXX/PP-1/XXXX/G3Px
AF271090.1 RVA/Canine-wt/XXX/RV52/96/1996/G3Px
LC158116 RVA/Bat-wt/ZMB/LUS12-14/2012/G3P[3]
LC328207 RVA/Cat-tc/JPN/FRV348/1994/G3P[3]
EU636932 RVA/Simian-tc/USA/RRV/1975/G3P[3]
EF200549.1 RVA/Bovine-wt/IND/RUBV3/XXXX/G3P[3]
KU597744 RVA/Human-wt/CHN/M2-102/2014/G3P[3]
AY456382 RVA/Human-wt/BEL/B4106/2000/G3P[14]
KJ879453 RVA/Rat-wt/GER/KS-11-573/2011/G3P[3]
KM515875.1 RVA/Horse-wt/ITA/EQ16/2013/G3P[12]
KP116019.1 RVA/Horse-wt/ARG/E4323/2009/G3P[12]
MF074190.1 RVA/Horse-wt/ARG/E1668/2012/G3P[12]
KP116026. 1 RVA/Horse-wt/ARG/E6000/2009/G3P[12]
JQ988904.1 RVA/Human-wt/Croatia/CR2006/2006/G8P[8]
Outgroup
III
IV
I
75
99
94
78
76
96
99
79
74
99
79
91
99
99
97
97
99
99
99
96
89
98
86
99
74
0.10

VP1-R2

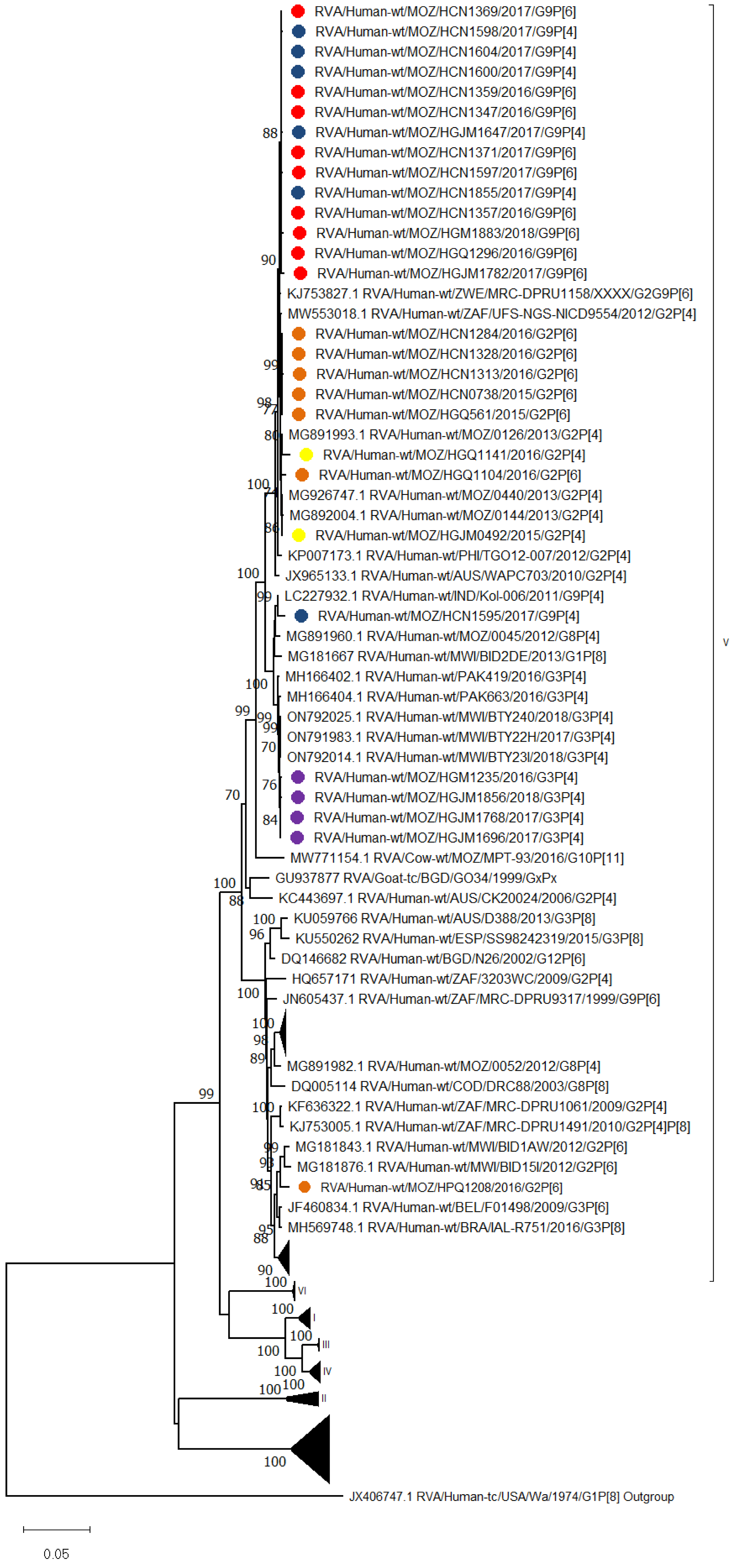

RVA/Human-wt/MOZ/HCN1369/2017/G9P[6]
RVA/Human-wt/MOZ/HCN1598/2017/G9P[4]
RVA/Human-wt/MOZ/HCN1604/2017/G9P[4]
RVA/Human-wt/MOZ/HCN1600/2017/G9P[4]
RVA/Human-wt/MOZ/HCN1359/2016/G9P[6]
RVA/Human-wt/MOZ/HCN1347/2016/G9P[6]
RVA/Human-wt/MOZ/HGJM1647/2017/G9P[4]
RVA/Human-wt/MOZ/HCN1371/2017/G9P[6]
RVA/Human-wt/MOZ/HCN1597/2017/G9P[6]
RVA/Human-wt/MOZ/HCN1855/2017/G9P[4]
RVA/Human-wt/MOZ/HCN1357/2016/G9P[6]
RVA/Human-wt/MOZ/HGM1883/2018/G9P[6]
RVA/Human-wt/MOZ/HGQ1296/2016/G9P[6]
RVA/Human-wt/MOZ/HGJM1782/2017/G9P[6]
KJ753827.1 RVA/Human-wt/ZWE/MRC-DPRU1158/XXXX/G2G9P[6]
MW553018.1 RVA/Human-wt/ZAF/UFS-NGS-NICD9554/2012/G2P[4]
RVA/Human-wt/MOZ/HCN1284/2016/G2P[6]
RVA/Human-wt/MOZ/HCN1328/2016/G2P[6]
RVA/Human-wt/MOZ/HCN1313/2016/G2P[6]
RVA/Human-wt/MOZ/HCN0738/2015/G2P[6]
RVA/Human-wt/MOZ/HGQ561/2015/G2P[6]
MG891993.1 RVA/Human-wt/MOZ/0126/2013/G2P[4]
RVA/Human-wt/MOZ/HGQ1141/2016/G2P[4]
RVA/Human-wt/MOZ/HGQ1104/2016/G2P[6]
MG926747.1 RVA/Human-wt/MOZ/0440/2013/G2P[4]
MG892004.1 RVA/Human-wt/MOZ/0144/2013/G2P[4]
RVA/Human-wt/MOZ/HGJM0492/2015/G2P[4]
KP007173.1 RVA/Human-wt/PHI/TGO12-007/2012/G2P[4]
JX965133.1 RVA/Human-wt/AUS/WAPC703/2010/G2P[4]
LC227932.1 RVA/Human-wt/IND/Kol-006/2011/G9P[4]
RVA/Human-wt/MOZ/HCN1595/2017/G9P[4]
MG891960.1 RVA/Human-wt/MOZ/0045/2012/G8P[4]
MG181667 RVA/Human-wt/MWI/BID2DE/2013/G1P[8]
MH166402.1 RVA/Human-wt/PAK419/2016/G3P[4]
MH166404.1 RVA/Human-wt/PAK663/2016/G3P[4]
ON792025.1 RVA/Human-wt/MWI/BTY240/2018/G3P[4]
ON791983.1 RVA/Human-wt/MWI/BTY22H/2017/G3P[4]
ON792014.1 RVA/Human-wt/MWI/BTY23I/2018/G3P[4]
RVA/Human-wt/MOZ/HGM1235/2016/G3P[4]
RVA/Human-wt/MOZ/HGJM1856/2018/G3P[4]
RVA/Human-wt/MOZ/HGJM1768/2017/G3P[4]
RVA/Human-wt/MOZ/HGJM1696/2017/G3P[4]
MW771154.1 RVA/Cow-wt/MOZ/MPT-93/2016/G10P[11]
GU937877 RVA/Goat-tc/BGD/GO34/1999/GxPx
KC443697.1 RVA/Human-wt/AUS/CK20024/2006/G2P[4]
KU059766 RVA/Human-wt/AUS/D388/2013/G3P[8]
KU550262 RVA/Human-wt/ESP/SS98242319/2015/G3P[8]
DQ146682 RVA/Human-wt/BGD/N26/2002/G12P[6]
HQ657171 RVA/Human-wt/ZAF/3203WC/2009/G2P[4]
JN605437.1 RVA/Human-wt/ZAF/MRC-DPRU9317/1999/G9P[6]
MG891982.1 RVA/Human-wt/MOZ/0052/2012/G8P[4]
DQ005114 RVA/Human-wt/COD/DRC88/2003/G8P[8]
KF636322.1 RVA/Human-wt/ZAF/MRC-DPRU1061/2009/G2P[4]
KJ753005.1 RVA/Human-wt/ZAF/MRC-DPRU1491/2010/G2P[4]P[8]
MG181843.1 RVA/Human-wt/MWI/BID1AW/2012/G2P[6]
MG181876.1 RVA/Human-wt/MWI/BID15I/2012/G2P[6]
RVA/Human-wt/MOZ/HPQ1208/2016/G2P[6]
JF460834.1 RVA/Human-wt/BEL/F01498/2009/G3P[6]
MH569748.1 RVA/Human-wt/BRA/IAL-R751/2016/G3P[8]
V
VI
I
III
IV
II
JX406747.1 RVA/Human-tc/USA/Wa/1974/G1P[8] Outgroup
0.05

VP2-C2

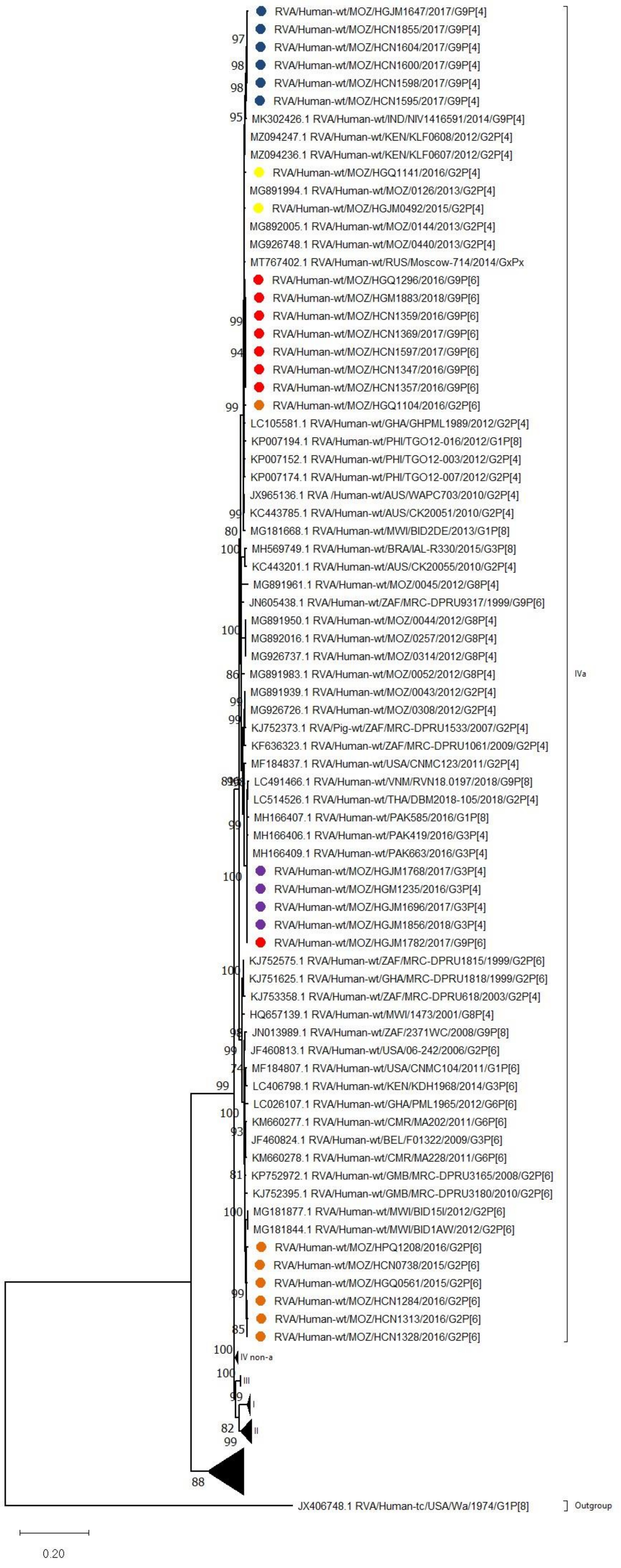

RVA/Human-wt/MOZ/HGJM1647/2017/G9P[4]
RVA/Human-wt/MOZ/HCN1855/2017/G9P[4]
RVA/Human-wt/MOZ/HCN1604/2017/G9P[4]
RVA/Human-wt/MOZ/HCN1600/2017/G9P[4]
RVA/Human-wt/MOZ/HCN1598/2017/G9P[4]
RVA/Human-wt/MOZ/HCN1595/2017/G9P[4]
MK302426.1 RVA/Human-wt/IND/NIV1416591/2014/G9P[4]
MZ094247.1 RVA/Human-wt/KEN/KLF0608/2012/G2P[4]
MZ094236.1 RVA/Human-wt/KEN/KLF0607/2012/G2P[4]
RVA/Human-wt/MOZ/HGQ1141/2016/G2P[4]
MG891994.1 RVA/Human-wt/MOZ/0126/2013/G2P[4]
RVA/Human-wt/MOZ/HGJM0492/2015/G2P[4]
MG892005.1 RVA/Human-wt/MOZ/0144/2013/G2P[4]
MG926748.1 RVA/Human-wt/MOZ/0440/2013/G2P[4]
MT767402.1 RVA/Human-wt/RUS/Moscow-714/2014/GxPx
RVA/Human-wt/MOZ/HGQ1296/2016/G9P[6]
RVA/Human-wt/MOZ/HGM1883/2018/G9P[6]
RVA/Human-wt/MOZ/HCN1359/2016/G9P[6]
RVA/Human-wt/MOZ/HCN1369/2017/G9P[6]
RVA/Human-wt/MOZ/HCN1597/2017/G9P[6]
RVA/Human-wt/MOZ/HCN1347/2016/G9P[6]
RVA/Human-wt/MOZ/HCN1357/2016/G9P[6]
RVA/Human-wt/MOZ/HGQ1104/2016/G2P[6]
LC105581.1 RVA/Human-wt/GHA/GHPML1989/2012/G2P[4]
KP007194.1 RVA/Human-wt/PHI/TGO12-016/2012/G1P[8]
KP007152.1 RVA/Human-wt/PHI/TGO12-003/2012/G2P[4]
KP007174.1 RVA/Human-wt/PHI/TGO12-007/2012/G2P[4]
JX965136.1 RVA /Human-wt/AUS/WAPC703/2010/G2P[4]
KC443785.1 RVA/Human-wt/AUS/CK20051/2010/G2P[4]
MG181668.1 RVA/Human-wt/MWI/BID2DE/2013/G1P[8]
MH569749.1 RVA/Human-wt/BRA/IAL-R330/2015/G3P[8]
KC443201.1 RVA/Human-wt/AUS/CK20055/2010/G2P[4]
MG891961.1 RVA/Human-wt/MOZ/0045/2012/G8P[4]
JN605438.1 RVA/Human-wt/ZAF/MRC-DPRU9317/1999/G9P[6]
MG891950.1 RVA/Human-wt/MOZ/0044/2012/G8P[4]
MG892016.1 RVA/Human-wt/MOZ/0257/2012/G8P[4]
MG926737.1 RVA/Human-wt/MOZ/0314/2012/G8P[4]
MG891983.1 RVA/Human-wt/MOZ/0052/2012/G8P[4]
MG891939.1 RVA/Human-wt/MOZ/0043/2012/G2P[4]
MG926726.1 RVA/Human-wt/MOZ/0308/2012/G2P[4]
KJ752373.1 RVA/Pig-wt/ZAF/MRC-DPRU1533/2007/G2P[4]
KF636323.1 RVA/Human-wt/ZAF/MRC-DPRU1061/2009/G2P[4]
MF184837.1 RVA/Human-wt/USA/CNMC123/2011/G2P[4]
LC491466.1 RVA/Human-wt/VNM/RVN18.0197/2018/G9P[8]
LC514526.1 RVA/Human-wt/THA/DBM2018-105/2018/G2P[4]
MH166407.1 RVA/Human-wt/PAK585/2016/G1P[8]
MH166406.1 RVA/Human-wt/PAK419/2016/G3P[4]
MH166409.1 RVA/Human-wt/PAK663/2016/G3P[4]
RVA/Human-wt/MOZ/HGJM1768/2017/G3P[4]
RVA/Human-wt/MOZ/HGM1235/2016/G3P[4]
RVA/Human-wt/MOZ/HGJM1696/2017/G3P[4]
RVA/Human-wt/MOZ/HGJM1856/2018/G3P[4]
RVA/Human-wt/MOZ/HGJM1782/2017/G9P[6]
KJ752575.1 RVA/Human-wt/ZAF/MRC-DPRU1815/1999/G2P[6]
KJ751625.1 RVA/Human-wt/GHA/MRC-DPRU1818/1999/G2P[6]
KJ753358.1 RVA/Human-wt/ZAF/MRC-DPRU618/2003/G2P[4]
HQ657139.1 RVA/Human-wt/MWI/1473/2001/G8P[4]
JN013989.1 RVA/Human-wt/ZAF/2371WC/2008/G9P[8]
JF460813.1 RVA/Human-wt/USA/06-242/2006/G2P[6]
MF184807.1 RVA/Human-wt/USA/CNMC104/2011/G1P[6]
LC406798.1 RVA/Human-wt/KEN/KDH1968/2014/G3P[6]
LC026107.1 RVA/Human-wt/GHA/PML1965/2012/G6P[6]
KM660277.1 RVA/Human-wt/CMR/MA202/2011/G6P[6]
JF460824.1 RVA/Human-wt/BEL/F01322/2009/G3P[6]
KM660278.1 RVA/Human-wt/CMR/MA228/2011/G6P[6]
KP752972.1 RVA/Human-wt/GMB/MRC-DPRU3165/2008/G2P[6]
KJ752395.1 RVA/Human-wt/GMB/MRC-DPRU3180/2010/G2P[6]
MG181877.1 RVA/Human-wt/MWI/BID15I/2012/G2P[6]
MG181844.1 RVA/Human-wt/MWI/BID1AW/2012/G2P[6]
RVA/Human-wt/MOZ/HPQ1208/2016/G2P[6]
RVA/Human-wt/MOZ/HCN0738/2015/G2P[6]
RVA/Human-wt/MOZ/HGQ0561/2015/G2P[6]
RVA/Human-wt/MOZ/HCN1284/2016/G2P[6]
RVA/Human-wt/MOZ/HCN1313/2016/G2P[6]
RVA/Human-wt/MOZ/HCN1328/2016/G2P[6]
IVa
IV non-a
III
I
II
JX406748.1 RVA/Human-tc/USA/Wa/1974/G1P[8]
Outgroup
0.20

VP3-M2

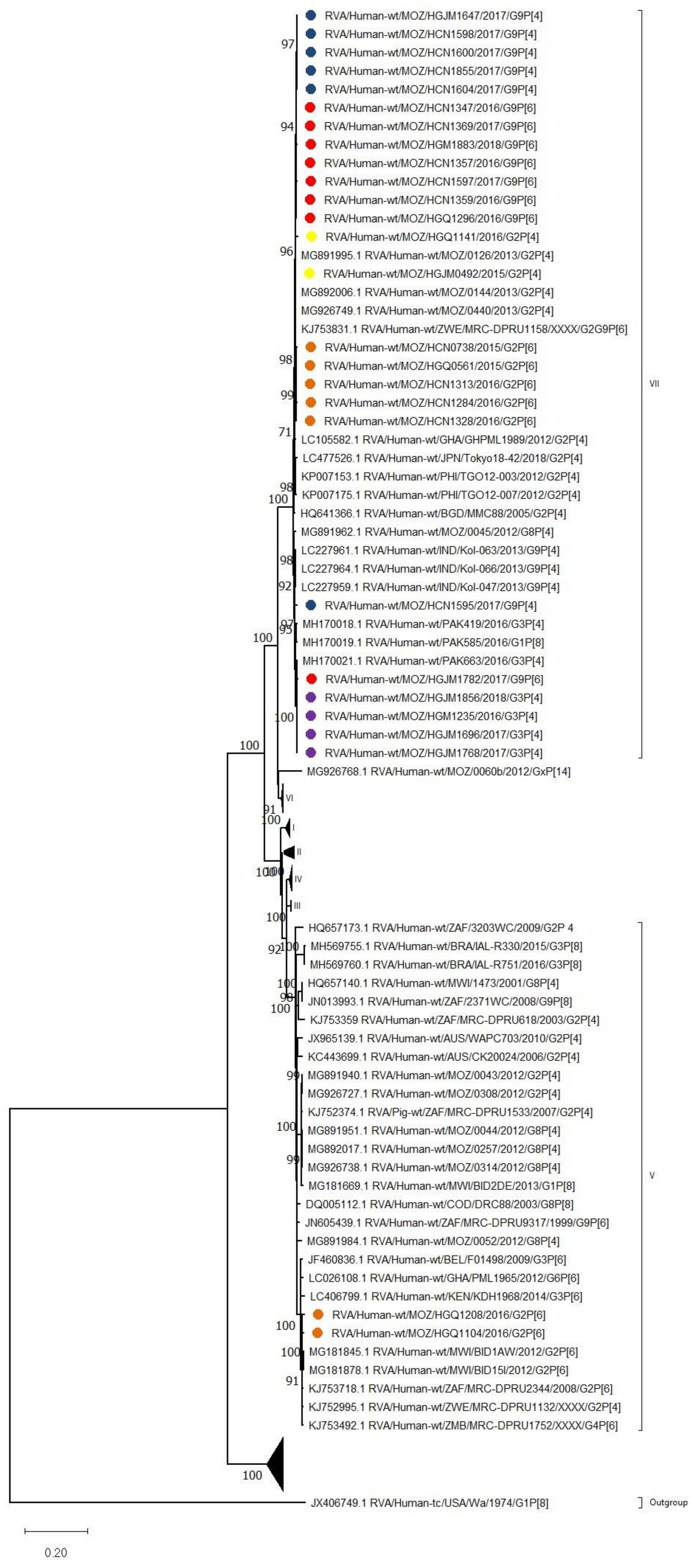

RVA/Human-wt/MOZ/HGJM1647/2017/G9P[4]
RVA/Human-wt/MOZ/HCN1598/2017/G9P[4]
RVA/Human-wt/MOZ/HCN1600/2017/G9P[4]
RVA/Human-wt/MOZ/HCN1855/2017/G9P[4]
RVA/Human-wt/MOZ/HCN1604/2017/G9P[4]
RVA/Human-wt/MOZ/HCN1347/2016/G9P[6]
RVA/Human-wt/MOZ/HCN1369/2017/G9P[6]
RVA/Human-wt/MOZ/HGM1883/2018/G9P[6]
RVA/Human-wt/MOZ/HCN1357/2016/G9P[6]
RVA/Human-wt/MOZ/HCN1597/2017/G9P[6]
RVA/Human-wt/MOZ/HCN1359/2016/G9P[6]
RVA/Human-wt/MOZ/HGQ1296/2016/G9P[6]
RVA/Human-wt/MOZ/HGQ1141/2016/G2P[4]
MG891995.1 RVA/Human-wt/MOZ/0126/2013/G2P[4]
RVA/Human-wt/MOZ/HGJM0492/2015/G2P[4]
MG892006.1 RVA/Human-wt/MOZ/0144/2013/G2P[4]
MG926749.1 RVA/Human-wt/MOZ/0440/2013/G2P[4]
KJ753831.1 RVA/Human-wt/ZWE/MRC-DPRU1158/XXXX/G2G9P[6]
RVA/Human-wt/MOZ/HCN0738/2015/G2P[6]
RVA/Human-wt/MOZ/HGQ0561/2015/G2P[6]
RVA/Human-wt/MOZ/HCN1313/2016/G2P[6]
RVA/Human-wt/MOZ/HCN1284/2016/G2P[6]
RVA/Human-wt/MOZ/HCN1328/2016/G2P[6]
LC105582.1 RVA/Human-wt/GHA/GHPML1989/2012/G2P[4]
LC477526.1 RVA/Human-wt/JPN/Tokyo18-42/2018/G2P[4]
KP007153.1 RVA/Human-wt/PHI/TGO12-003/2012/G2P[4]
KP007175.1 RVA/Human-wt/PHI/TGO12-007/2012/G2P[4]
HQ641366.1 RVA/Human-wt/BGD/MMC88/2005/G2P[4]
MG891962.1 RVA/Human-wt/MOZ/0045/2012/G8P[4]
LC227961.1 RVA/Human-wt/IND/Kol-063/2013/G9P[4]
LC227964.1 RVA/Human-wt/IND/Kol-066/2013/G9P[4]
LC227959.1 RVA/Human-wt/IND/Kol-047/2013/G9P[4]
RVA/Human-wt/MOZ/HCN1595/2017/G9P[4]
MH170018.1 RVA/Human-wt/PAK419/2016/G3P[4]
MH170019.1 RVA/Human-wt/PAK585/2016/G1P[8]
MH170021.1 RVA/Human-wt/PAK663/2016/G3P[4]
RVA/Human-wt/MOZ/HGJM1782/2017/G9P[6]
RVA/Human-wt/MOZ/HGJM1856/2018/G3P[4]
RVA/Human-wt/MOZ/HGM1235/2016/G3P[4]
RVA/Human-wt/MOZ/HGJM1696/2017/G3P[4]
RVA/Human-wt/MOZ/HGJM1768/2017/G3P[4]
VII
MG926768.1 RVA/Human-wt/MOZ/0060b/2012/GxP[14]
VI
I
II
IV
III
HQ657173.1 RVA/Human-wt/ZAF/3203WC/2009/G2P 4
MH569755.1 RVA/Human-wt/BRA/IAL-R330/2015/G3P[8]
MH569760.1 RVA/Human-wt/BRA/IAL-R751/2016/G3P[8]
HQ657140.1 RVA/Human-wt/MWI/1473/2001/G8P[4]
JN013993.1 RVA/Human-wt/ZAF/2371WC/2008/G9P[8]
KJ753359 RVA/Human-wt/ZAF/MRC-DPRU618/2003/G2P[4]
JX965139.1 RVA/Human-wt/AUS/WAPC703/2010/G2P[4]
KC443699.1 RVA/Human-wt/AUS/CK20024/2006/G2P[4]
MG891940.1 RVA/Human-wt/MOZ/0043/2012/G2P[4]
MG926727.1 RVA/Human-wt/MOZ/0308/2012/G2P[4]
KJ752374.1 RVA/Pig-wt/ZAF/MRC-DPRU1533/2007/G2P[4]
MG891951.1 RVA/Human-wt/MOZ/0044/2012/G8P[4]
MG892017.1 RVA/Human-wt/MOZ/0257/2012/G8P[4]
MG926738.1 RVA/Human-wt/MOZ/0314/2012/G8P[4]
MG181669.1 RVA/Human-wt/MWI/BID2DE/2013/G1P[8]
DQ005112.1 RVA/Human-wt/COD/DRC88/2003/G8P[8]
JN605439.1 RVA/Human-wt/ZAF/MRC-DPRU9317/1999/G9P[6]
MG891984.1 RVA/Human-wt/MOZ/0052/2012/G8P[4]
JF460836.1 RVA/Human-wt/BEL/F01498/2009/G3P[6]
LC026108.1 RVA/Human-wt/GHA/PML1965/2012/G6P[6]
LC406799.1 RVA/Human-wt/KEN/KDH1968/2014/G3P[6]
RVA/Human-wt/MOZ/HGQ1208/2016/G2P[6]
RVA/Human-wt/MOZ/HGQ1104/2016/G2P[6]
MG181845.1 RVA/Human-wt/MWI/BID1AW/2012/G2P[6]
MG181878.1 RVA/Human-wt/MWI/BID15I/2012/G2P[6]
KJ753718.1 RVA/Human-wt/ZAF/MRC-DPRU2344/2008/G2P[6]
KJ752995.1 RVA/Human-wt/ZWE/MRC-DPRU1132/XXXX/G2P[4]
KJ753492.1 RVA/Human-wt/ZMB/MRC-DPRU1752/XXXX/G4P[6]
V
JX406749.1 RVA/Human-tc/USA/Wa/1974/G1P[8]
Outgroup
0.20

NSP1-A2

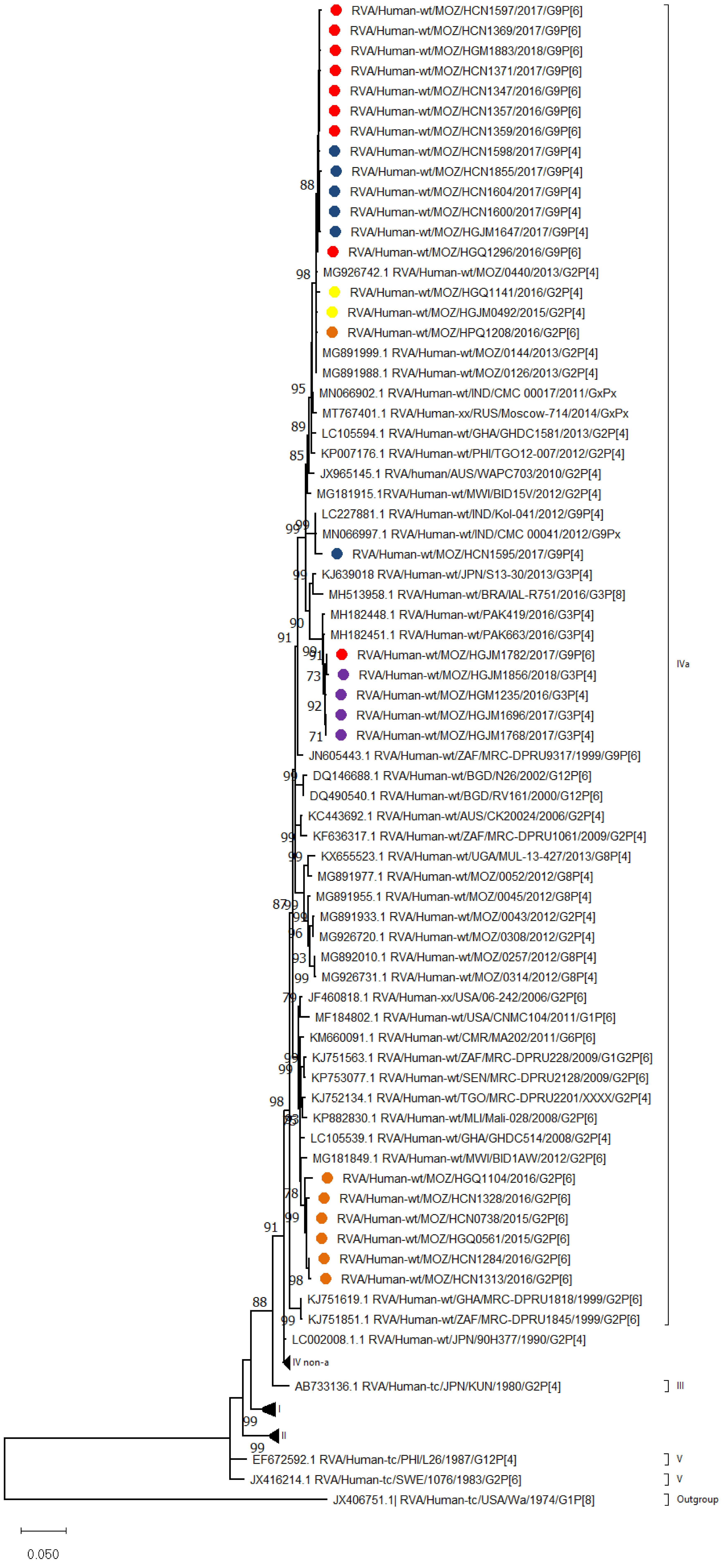

RVA/Human-wt/MOZ/HCN1597/2017/G9P[6]
RVA/Human-wt/MOZ/HCN1369/2017/G9P[6]
RVA/Human-wt/MOZ/HGM1883/2018/G9P[6]
RVA/Human-wt/MOZ/HCN1371/2017/G9P[6]
RVA/Human-wt/MOZ/HCN1347/2016/G9P[6]
RVA/Human-wt/MOZ/HCN1357/2016/G9P[6]
RVA/Human-wt/MOZ/HCN1359/2016/G9P[6]
RVA/Human-wt/MOZ/HCN1598/2017/G9P[4]
RVA/Human-wt/MOZ/HCN1855/2017/G9P[4]
RVA/Human-wt/MOZ/HCN1604/2017/G9P[4]
RVA/Human-wt/MOZ/HCN1600/2017/G9P[4]
RVA/Human-wt/MOZ/HGJM1647/2017/G9P[4]
RVA/Human-wt/MOZ/HGQ1296/2016/G9P[6]
MG926742.1 RVA/Human-wt/MOZ/0440/2013/G2P[4]
RVA/Human-wt/MOZ/HGQ1141/2016/G2P[4]
RVA/Human-wt/MOZ/HGJM0492/2015/G2P[4]
RVA/Human-wt/MOZ/HPQ1208/2016/G2P[6]
MG891999.1 RVA/Human-wt/MOZ/0144/2013/G2P[4]
MG891988.1 RVA/Human-wt/MOZ/0126/2013/G2P[4]
MN066902.1 RVA/Human-wt/IND/CMC 00017/2011/GxPx
MT767401.1 RVA/Human-xx/RUS/Moscow-714/2014/GxPx
LC105594.1 RVA/Human-wt/GHA/GHDC1581/2013/G2P[4]
KP007176.1 RVA/Human-wt/PHI/TGO12-007/2012/G2P[4]
JX965145.1 RVA/human/AUS/WAPC703/2010/G2P[4]
MG181915.1RVA/Human-wt/MWI/BID15V/2012/G2P[4]
LC227881.1 RVA/Human-wt/IND/Kol-041/2012/G9P[4]
MN066997.1 RVA/Human-wt/IND/CMC 00041/2012/G9Px
RVA/Human-wt/MOZ/HCN1595/2017/G9P[4]
KJ639018 RVA/Human-wt/JPN/S13-30/2013/G3P[4]
MH513958.1 RVA/Human-wt/BRA/IAL-R751/2016/G3P[8]
MH182448.1 RVA/Human-wt/PAK419/2016/G3P[4]
MH182451.1 RVA/Human-wt/PAK663/2016/G3P[4]
RVA/Human-wt/MOZ/HGJM1782/2017/G9P[6]
RVA/Human-wt/MOZ/HGJM1856/2018/G3P[4]
RVA/Human-wt/MOZ/HGM1235/2016/G3P[4]
RVA/Human-wt/MOZ/HGJM1696/2017/G3P[4]
RVA/Human-wt/MOZ/HGJM1768/2017/G3P[4]
JN605443.1 RVA/Human-wt/ZAF/MRC-DPRU9317/1999/G9P[6]
DQ146688.1 RVA/Human-wt/BGD/N26/2002/G12P[6]
DQ490540.1 RVA/Human-wt/BGD/RV161/2000/G12P[6]
KC443692.1 RVA/Human-wt/AUS/CK20024/2006/G2P[4]
KF636317.1 RVA/Human-wt/ZAF/MRC-DPRU1061/2009/G2P[4]
KX655523.1 RVA/Human-wt/UGA/MUL-13-427/2013/G8P[4]
MG891977.1 RVA/Human-wt/MOZ/0052/2012/G8P[4]
MG891955.1 RVA/Human-wt/MOZ/0045/2012/G8P[4]
MG891933.1 RVA/Human-wt/MOZ/0043/2012/G2P[4]
MG926720.1 RVA/Human-wt/MOZ/0308/2012/G2P[4]
MG892010.1 RVA/Human-wt/MOZ/0257/2012/G8P[4]
MG926731.1 RVA/Human-wt/MOZ/0314/2012/G8P[4]
JF460818.1 RVA/Human-xx/USA/06-242/2006/G2P[6]
MF184802.1 RVA/Human-wt/USA/CNMC104/2011/G1P[6]
KM660091.1 RVA/Human-wt/CMR/MA202/2011/G6P[6]
KJ751563.1 RVA/Human-wt/ZAF/MRC-DPRU228/2009/G1G2P[6]
KP753077.1 RVA/Human-wt/SEN/MRC-DPRU2128/2009/G2P[6]
KJ752134.1 RVA/Human-wt/TGO/MRC-DPRU2201/XXXX/G2P[4]
KP882830.1 RVA/Human-wt/MLI/Mali-028/2008/G2P[6]
LC105539.1 RVA/Human-wt/GHA/GHDC514/2008/G2P[4]
MG181849.1 RVA/Human-wt/MWI/BID1AW/2012/G2P[6]
RVA/Human-wt/MOZ/HGQ1104/2016/G2P[6]
RVA/Human-wt/MOZ/HCN1328/2016/G2P[6]
RVA/Human-wt/MOZ/HCN0738/2015/G2P[6]
RVA/Human-wt/MOZ/HGQ0561/2015/G2P[6]
RVA/Human-wt/MOZ/HCN1284/2016/G2P[6]
RVA/Human-wt/MOZ/HCN1313/2016/G2P[6]
KJ751619.1 RVA/Human-wt/GHA/MRC-DPRU1818/1999/G2P[6]
KJ751851.1 RVA/Human-wt/ZAF/MRC-DPRU1845/1999/G2P[6]
LC002008.1.1 RVA/Human-wt/JPN/90H377/1990/G2P[4]
IV non-a
AB733136.1 RVA/Human-tc/JPN/KUN/1980/G2P[4]
I
II
EF672592.1 RVA/Human-tc/PHI/L26/1987/G12P[4]
JX416214.1 RVA/Human-tc/SWE/1076/1983/G2P[6]
JX406751.1| RVA/Human-tc/USA/Wa/1974/G1P[8]
IVa
III
V
V
Outgroup
0.050

NSP2-N2

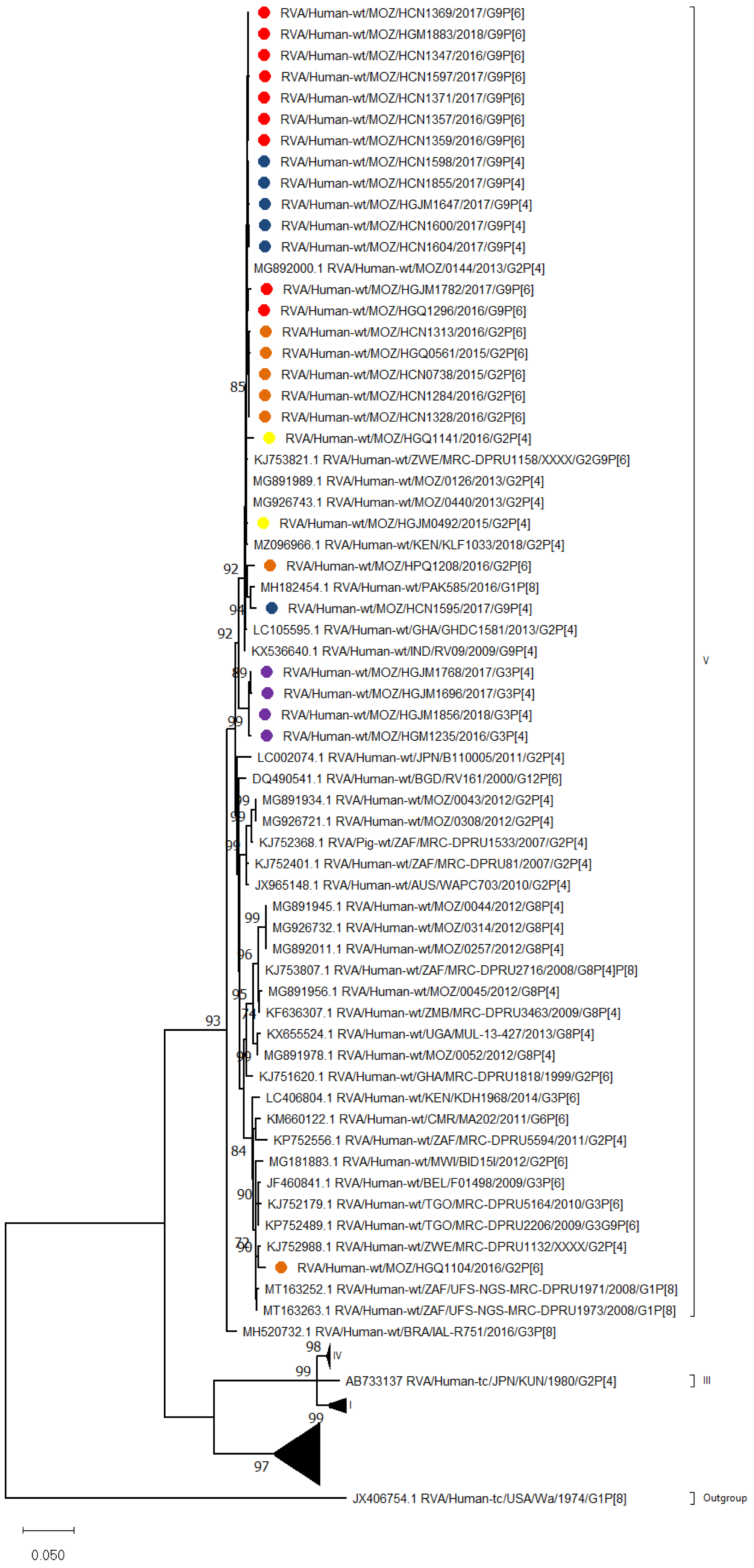
RVA/Human-wt/MOZ/HCN1369/2017/G9P[6]
RVA/Human-wt/MOZ/HGM1883/2018/G9P[6]
RVA/Human-wt/MOZ/HCN1347/2016/G9P[6]
RVA/Human-wt/MOZ/HCN1597/2017/G9P[6]
RVA/Human-wt/MOZ/HCN1371/2017/G9P[6]
RVA/Human-wt/MOZ/HCN1357/2016/G9P[6]
RVA/Human-wt/MOZ/HCN1359/2016/G9P[6]
RVA/Human-wt/MOZ/HCN1598/2017/G9P[4]
RVA/Human-wt/MOZ/HCN1855/2017/G9P[4]
RVA/Human-wt/MOZ/HGJM1647/2017/G9P[4]
RVA/Human-wt/MOZ/HCN1600/2017/G9P[4]
RVA/Human-wt/MOZ/HCN1604/2017/G9P[4]
MG892000.1 RVA/Human-wt/MOZ/0144/2013/G2P[4]
RVA/Human-wt/MOZ/HGJM1782/2017/G9P[6]
RVA/Human-wt/MOZ/HGQ1296/2016/G9P[6]
RVA/Human-wt/MOZ/HCN1313/2016/G2P[6]
RVA/Human-wt/MOZ/HGQ0561/2015/G2P[6]
85
RVA/Human-wt/MOZ/HCN0738/2015/G2P[6]
RVA/Human-wt/MOZ/HCN1284/2016/G2P[6]
RVA/Human-wt/MOZ/HCN1328/2016/G2P[6]
RVA/Human-wt/MOZ/HGQ1141/2016/G2P[4]
KJ753821.1 RVA/Human-wt/ZWE/MRC-DPRU1158/XXXX/G2G9P[6]
MG891989.1 RVA/Human-wt/MOZ/0126/2013/G2P[4]
MG926743.1 RVA/Human-wt/MOZ/0440/2013/G2P[4]
RVA/Human-wt/MOZ/HGJM0492/2015/G2P[4]
MZ096966.1 RVA/Human-wt/KEN/KLF1033/2018/G2P[4]
92
RVA/Human-wt/MOZ/HPQ1208/2016/G2P[6]
MH182454.1 RVA/Human-wt/PAK585/2016/G1P[8]
94
RVA/Human-wt/MOZ/HCN1595/2017/G9P[4]
92
LC105595.1 RVA/Human-wt/GHA/GHDC1581/2013/G2P[4]
KX536640.1 RVA/Human-wt/IND/RV09/2009/G9P[4]
V
89
RVA/Human-wt/MOZ/HGJM1768/2017/G3P[4]
RVA/Human-wt/MOZ/HGJM1696/2017/G3P[4]
RVA/Human-wt/MOZ/HGJM1856/2018/G3P[4]
99
RVA/Human-wt/MOZ/HGM1235/2016/G3P[4]
LC002074.1 RVA/Human-wt/JPN/B110005/2011/G2P[4]
DQ490541.1 RVA/Human-wt/BGD/RV161/2000/G12P[6]
99
MG891934.1 RVA/Human-wt/MOZ/0043/2012/G2P[4]
99
MG926721.1 RVA/Human-wt/MOZ/0308/2012/G2P[4]
KJ752368.1 RVA/Pig-wt/ZAF/MRC-DPRU1533/2007/G2P[4]
99
KJ752401.1 RVA/Human-wt/ZAF/MRC-DPRU81/2007/G2P[4]
JX965148.1 RVA/Human-wt/AUS/WAPC703/2010/G2P[4]
MG891945.1 RVA/Human-wt/MOZ/0044/2012/G8P[4]
99
MG926732.1 RVA/Human-wt/MOZ/0314/2012/G8P[4]
MG892011.1 RVA/Human-wt/MOZ/0257/2012/G8P[4]
96
KJ753807.1 RVA/Human-wt/ZAF/MRC-DPRU2716/2008/G8P[4]P[8]
95
MG891956.1 RVA/Human-wt/MOZ/0045/2012/G8P[4]
74
KF636307.1 RVA/Human-wt/ZMB/MRC-DPRU3463/2009/G8P[4]
KX655524.1 RVA/Human-wt/UGA/MUL-13-427/2013/G8P[4]
93
99
MG891978.1 RVA/Human-wt/MOZ/0052/2012/G8P[4]
KJ751620.1 RVA/Human-wt/GHA/MRC-DPRU1818/1999/G2P[6]
LC406804.1 RVA/Human-wt/KEN/KDH1968/2014/G3P[6]
KM660122.1 RVA/Human-wt/CMR/MA202/2011/G6P[6]
KP752556.1 RVA/Human-wt/ZAF/MRC-DPRU5594/2011/G2P[4]
84
MG181883.1 RVA/Human-wt/MWI/BID15I/2012/G2P[6]
JF460841.1 RVA/Human-wt/BEL/F01498/2009/G3P[6]
90
KJ752179.1 RVA/Human-wt/TGO/MRC-DPRU5164/2010/G3P[6]
KP752489.1 RVA/Human-wt/TGO/MRC-DPRU2206/2009/G3G9P[6]
72
90
KJ752988.1 RVA/Human-wt/ZWE/MRC-DPRU1132/XXXX/G2P[4]
RVA/Human-wt/MOZ/HGQ1104/2016/G2P[6]
MT163252.1 RVA/Human-wt/ZAF/UFS-NGS-MRC-DPRU1971/2008/G1P[8]
MT163263.1 RVA/Human-wt/ZAF/UFS-NGS-MRC-DPRU1973/2008/G1P[8]
MH520732.1 RVA/Human-wt/BRA/IAL-R751/2016/G3P[8]
98
IV
99
AB733137 RVA/Human-tc/JPN/KUN/1980/G2P[4]
III
I
99
97
JX406754.1 RVA/Human-tc/USA/Wa/1974/G1P[8]
Outgroup
0.050

NSP3-T2

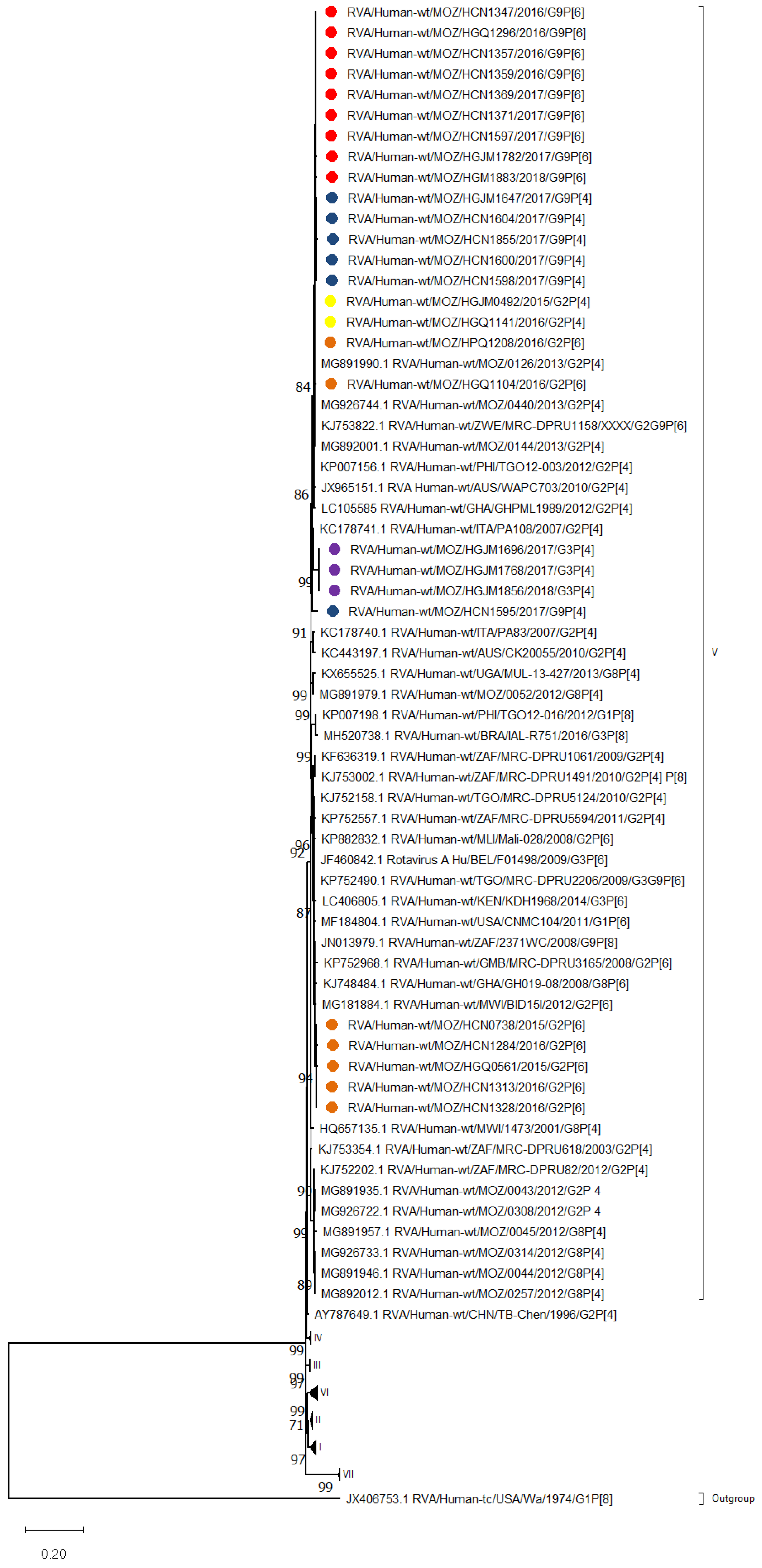
RVA/Human-wt/MOZ/HCN1347/2016/G9P[6]
RVA/Human-wt/MOZ/HGQ1296/2016/G9P[6]
RVA/Human-wt/MOZ/HCN1357/2016/G9P[6]
RVA/Human-wt/MOZ/HCN1359/2016/G9P[6]
RVA/Human-wt/MOZ/HCN1369/2017/G9P[6]
RVA/Human-wt/MOZ/HCN1371/2017/G9P[6]
RVA/Human-wt/MOZ/HCN1597/2017/G9P[6]
RVA/Human-wt/MOZ/HGJM1782/2017/G9P[6]
RVA/Human-wt/MOZ/HGM1883/2018/G9P[6]
RVA/Human-wt/MOZ/HGJM1647/2017/G9P[4]
RVA/Human-wt/MOZ/HCN1604/2017/G9P[4]
RVA/Human-wt/MOZ/HCN1855/2017/G9P[4]
RVA/Human-wt/MOZ/HCN1600/2017/G9P[4]
RVA/Human-wt/MOZ/HCN1598/2017/G9P[4]
RVA/Human-wt/MOZ/HGJM0492/2015/G2P[4]
RVA/Human-wt/MOZ/HGQ1141/2016/G2P[4]
RVA/Human-wt/MOZ/HPQ1208/2016/G2P[6]
MG891990.1 RVA/Human-wt/MOZ/0126/2013/G2P[4]
RVA/Human-wt/MOZ/HGQ1104/2016/G2P[6]
MG926744.1 RVA/Human-wt/MOZ/0440/2013/G2P[4]
KJ753822.1 RVA/Human-wt/ZWE/MRC-DPRU1158/XXXX/G2G9P[6]
MG892001.1 RVA/Human-wt/MOZ/0144/2013/G2P[4]
KP007156.1 RVA/Human-wt/PHI/TGO12-003/2012/G2P[4]
JX965151.1 RVA Human-wt/AUS/WAPC703/2010/G2P[4]
LC105585 RVA/Human-wt/GHA/GHPML1989/2012/G2P[4]
KC178741.1 RVA/Human-wt/ITA/PA108/2007/G2P[4]
RVA/Human-wt/MOZ/HGJM1696/2017/G3P[4]
RVA/Human-wt/MOZ/HGJM1768/2017/G3P[4]
RVA/Human-wt/MOZ/HGJM1856/2018/G3P[4]
RVA/Human-wt/MOZ/HCN1595/2017/G9P[4]
KC178740.1 RVA/Human-wt/ITA/PA83/2007/G2P[4]
KC443197.1 RVA/Human-wt/AUS/CK20055/2010/G2P[4]
KX655525.1 RVA/Human-wt/UGA/MUL-13-427/2013/G8P[4]
MG891979.1 RVA/Human-wt/MOZ/0052/2012/G8P[4]
KP007198.1 RVA/Human-wt/PHI/TGO12-016/2012/G1P[8]
MH520738.1 RVA/Human-wt/BRA/IAL-R751/2016/G3P[8]
KF636319.1 RVA/Human-wt/ZAF/MRC-DPRU1061/2009/G2P[4]
KJ753002.1 RVA/Human-wt/ZAF/MRC-DPRU1491/2010/G2P[4] P[8]
KJ752158.1 RVA/Human-wt/TGO/MRC-DPRU5124/2010/G2P[4]
KP752557.1 RVA/Human-wt/ZAF/MRC-DPRU5594/2011/G2P[4]
KP882832.1 RVA/Human-wt/MLI/Mali-028/2008/G2P[6]
JF460842.1 Rotavirus A Hu/BEL/F01498/2009/G3P[6]
KP752490.1 RVA/Human-wt/TGO/MRC-DPRU2206/2009/G3G9P[6]
LC406805.1 RVA/Human-wt/KEN/KDH1968/2014/G3P[6]
MF184804.1 RVA/Human-wt/USA/CNMC104/2011/G1P[6]
JN013979.1 RVA/Human-wt/ZAF/2371WC/2008/G9P[8]
KP752968.1 RVA/Human-wt/GMB/MRC-DPRU3165/2008/G2P[6]
KJ748484.1 RVA/Human-wt/GHA/GH019-08/2008/G8P[6]
MG181884.1 RVA/Human-wt/MWI/BID15I/2012/G2P[6]
RVA/Human-wt/MOZ/HCN0738/2015/G2P[6]
RVA/Human-wt/MOZ/HCN1284/2016/G2P[6]
RVA/Human-wt/MOZ/HGQ0561/2015/G2P[6]
RVA/Human-wt/MOZ/HCN1313/2016/G2P[6]
RVA/Human-wt/MOZ/HCN1328/2016/G2P[6]
HQ657135.1 RVA/Human-wt/MWI/1473/2001/G8P[4]
KJ753354.1 RVA/Human-wt/ZAF/MRC-DPRU618/2003/G2P[4]
KJ752202.1 RVA/Human-wt/ZAF/MRC-DPRU82/2012/G2P[4]
MG891935.1 RVA/Human-wt/MOZ/0043/2012/G2P 4
MG926722.1 RVA/Human-wt/MOZ/0308/2012/G2P 4
MG891957.1 RVA/Human-wt/MOZ/0045/2012/G8P[4]
MG926733.1 RVA/Human-wt/MOZ/0314/2012/G8P[4]
MG891946.1 RVA/Human-wt/MOZ/0044/2012/G8P[4]
MG892012.1 RVA/Human-wt/MOZ/0257/2012/G8P[4]
V
AY787649.1 RVA/Human-wt/CHN/TB-Chen/1996/G2P[4]
IV
III
VI
II
I
VII
JX406753.1 RVA/Human-tc/USA/Wa/1974/G1P[8]
Outgroup
0.20

NSP4-E6

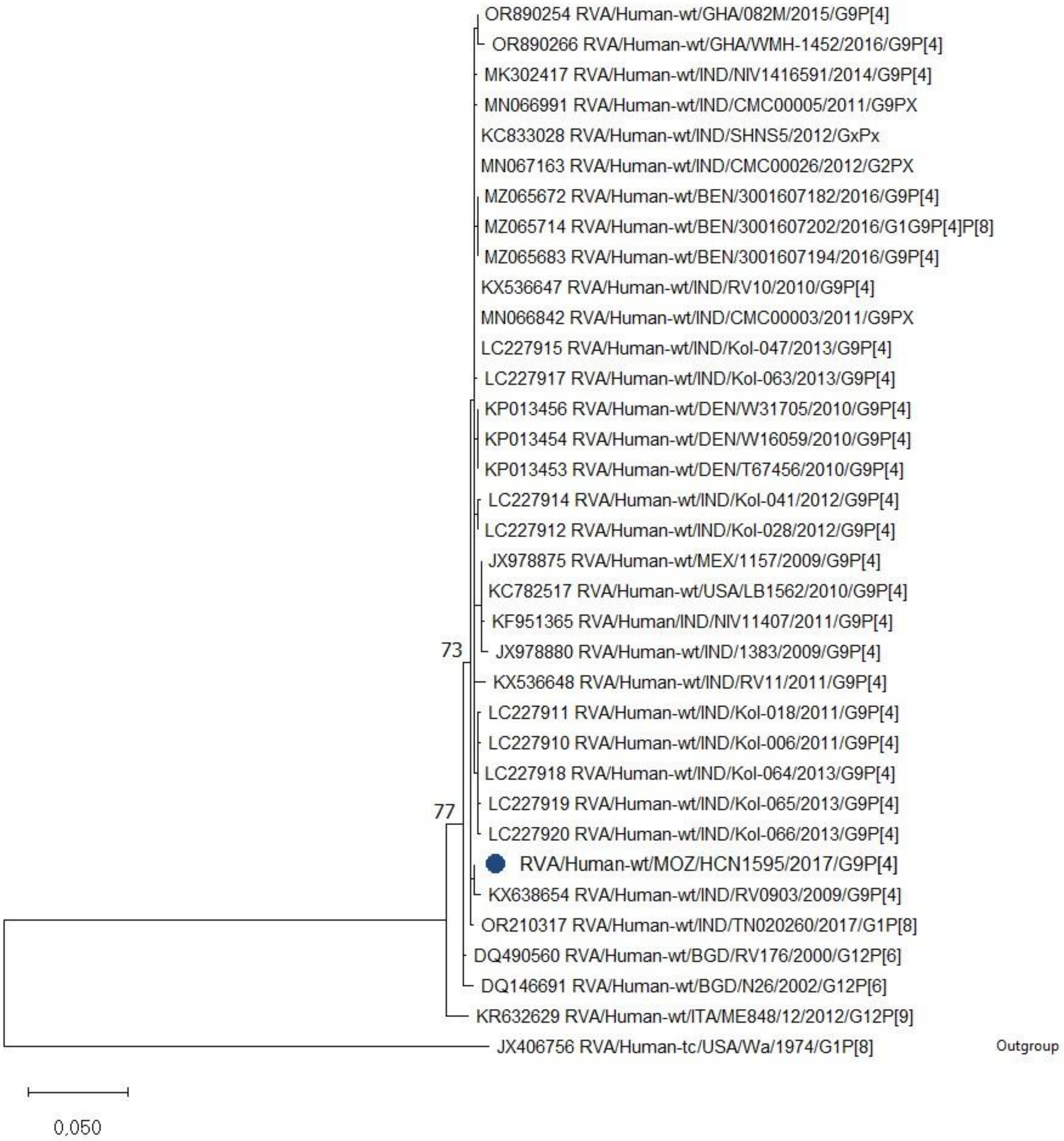
OR890254 RVA/Human-wt/GHA/082M/2015/G9P[4]
OR890266 RVA/Human-wt/GHA/WMH-1452/2016/G9P[4]
MK302417 RVA/Human-wt/IND/NIV1416591/2014/G9P[4]
MN066991 RVA/Human-wt/IND/CMC00005/2011/G9PX
KC833028 RVA/Human-wt/IND/SHNS5/2012/GxPx
MN067163 RVA/Human-wt/IND/CMC00026/2012/G2PX
MZ065672 RVA/Human-wt/BEN/3001607182/2016/G9P[4]
MZ065714 RVA/Human-wt/BEN/3001607202/2016/G1G9P[4]P[8]
MZ065683 RVA/Human-wt/BEN/3001607194/2016/G9P[4]
KX536647 RVA/Human-wt/IND/RV10/2010/G9P[4]
MN066842 RVA/Human-wt/IND/CMC00003/2011/G9PX
LC227915 RVA/Human-wt/IND/Kol-047/2013/G9P[4]
LC227917 RVA/Human-wt/IND/Kol-063/2013/G9P[4]
KP013456 RVA/Human-wt/DEN/W31705/2010/G9P[4]
KP013454 RVA/Human-wt/DEN/W16059/2010/G9P[4]
KP013453 RVA/Human-wt/DEN/T67456/2010/G9P[4]
LC227914 RVA/Human-wt/IND/Kol-041/2012/G9P[4]
LC227912 RVA/Human-wt/IND/Kol-028/2012/G9P[4]
JX978875 RVA/Human-wt/MEX/1157/2009/G9P[4]
KC782517 RVA/Human-wt/USA/LB1562/2010/G9P[4]
KF951365 RVA/Human/IND/NIV11407/2011/G9P[4]
JX978880 RVA/Human-wt/IND/1383/2009/G9P[4]
73
KX536648 RVA/Human-wt/IND/RV11/2011/G9P[4]
LC227911 RVA/Human-wt/IND/Kol-018/2011/G9P[4]
LC227910 RVA/Human-wt/IND/Kol-006/2011/G9P[4]
LC227918 RVA/Human-wt/IND/Kol-064/2013/G9P[4]
LC227919 RVA/Human-wt/IND/Kol-065/2013/G9P[4]
77
LC227920 RVA/Human-wt/IND/Kol-066/2013/G9P[4]
RVA/Human-wt/MOZ/HCN1595/2017/G9P[4]
KX638654 RVA/Human-wt/IND/RV0903/2009/G9P[4]
OR210317 RVA/Human-wt/IND/TN020260/2017/G1P[8]
DQ490560 RVA/Human-wt/BGD/RV176/2000/G12P[6]
DQ146691 RVA/Human-wt/BGD/N26/2002/G12P[6]
KR632629 RVA/Human-wt/ITA/ME848/12/2012/G12P[9]
JX406756 RVA/Human-tc/USA/Wa/1974/G1P[8]
Outgroup
0,050

NSP4-E2

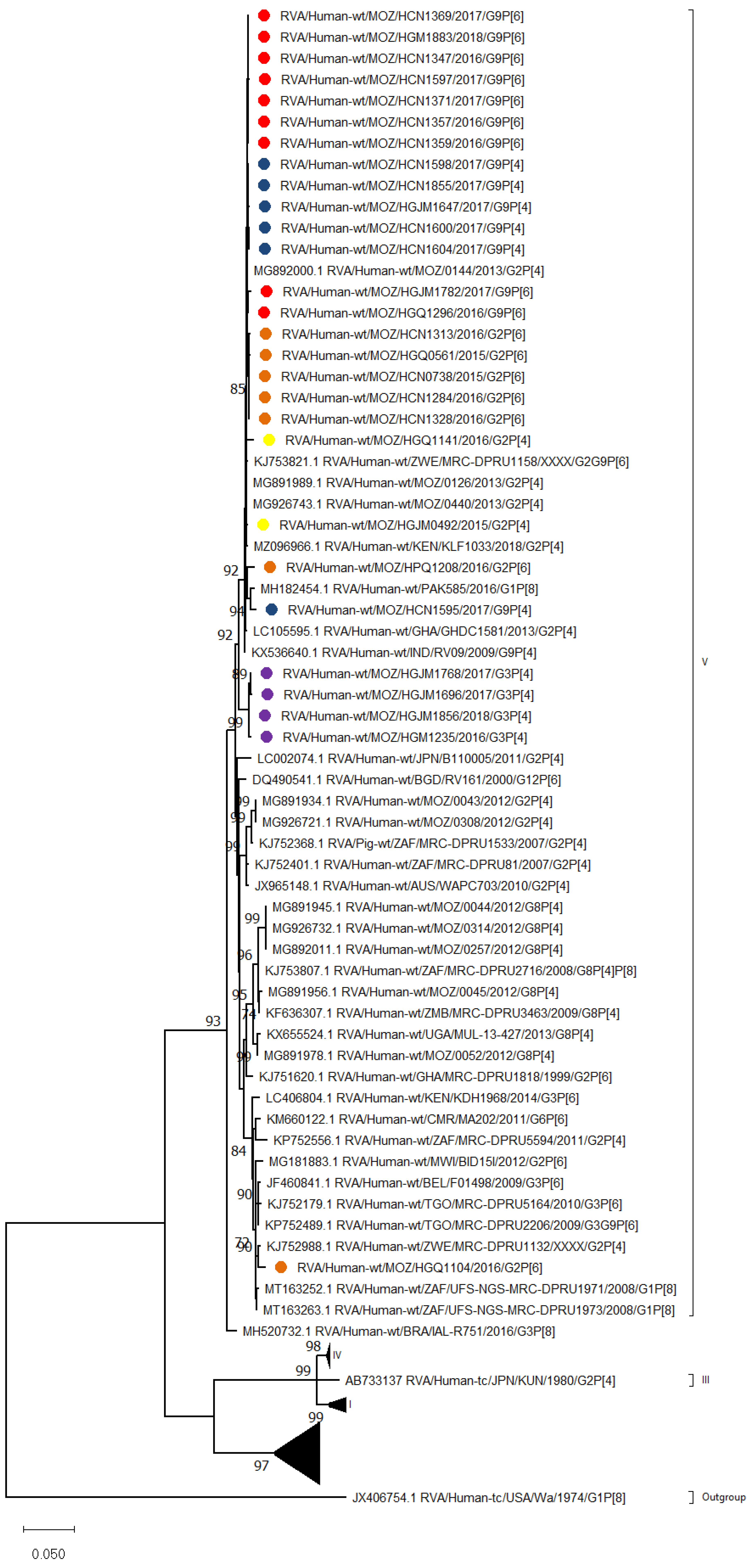
RVA/Human-wt/MOZ/HCN1369/2017/G9P[6]
RVA/Human-wt/MOZ/HGM1883/2018/G9P[6]
RVA/Human-wt/MOZ/HCN1347/2016/G9P[6]
RVA/Human-wt/MOZ/HCN1597/2017/G9P[6]
RVA/Human-wt/MOZ/HCN1371/2017/G9P[6]
RVA/Human-wt/MOZ/HCN1357/2016/G9P[6]
RVA/Human-wt/MOZ/HCN1359/2016/G9P[6]
RVA/Human-wt/MOZ/HCN1598/2017/G9P[4]
RVA/Human-wt/MOZ/HCN1855/2017/G9P[4]
RVA/Human-wt/MOZ/HGJM1647/2017/G9P[4]
RVA/Human-wt/MOZ/HCN1600/2017/G9P[4]
RVA/Human-wt/MOZ/HCN1604/2017/G9P[4]
MG892000.1 RVA/Human-wt/MOZ/0144/2013/G2P[4]
RVA/Human-wt/MOZ/HGJM1782/2017/G9P[6]
RVA/Human-wt/MOZ/HGQ1296/2016/G9P[6]
RVA/Human-wt/MOZ/HCN1313/2016/G2P[6]
RVA/Human-wt/MOZ/HGQ0561/2015/G2P[6]
RVA/Human-wt/MOZ/HCN0738/2015/G2P[6]
RVA/Human-wt/MOZ/HCN1284/2016/G2P[6]
RVA/Human-wt/MOZ/HCN1328/2016/G2P[6]
RVA/Human-wt/MOZ/HGQ1141/2016/G2P[4]
KJ753821.1 RVA/Human-wt/ZWE/MRC-DPRU1158/XXXX/G2G9P[6]
MG891989.1 RVA/Human-wt/MOZ/0126/2013/G2P[4]
MG926743.1 RVA/Human-wt/MOZ/0440/2013/G2P[4]
RVA/Human-wt/MOZ/HGJM0492/2015/G2P[4]
MZ096966.1 RVA/Human-wt/KEN/KLF1033/2018/G2P[4]
RVA/Human-wt/MOZ/HPQ1208/2016/G2P[6]
MH182454.1 RVA/Human-wt/PAK585/2016/G1P[8]
RVA/Human-wt/MOZ/HCN1595/2017/G9P[4]
LC105595.1 RVA/Human-wt/GHA/GHDC1581/2013/G2P[4]
KX536640.1 RVA/Human-wt/IND/RV09/2009/G9P[4]
RVA/Human-wt/MOZ/HGJM1768/2017/G3P[4]
RVA/Human-wt/MOZ/HGJM1696/2017/G3P[4]
RVA/Human-wt/MOZ/HGJM1856/2018/G3P[4]
RVA/Human-wt/MOZ/HGM1235/2016/G3P[4]
LC002074.1 RVA/Human-wt/JPN/B110005/2011/G2P[4]
DQ490541.1 RVA/Human-wt/BGD/RV161/2000/G12P[6]
MG891934.1 RVA/Human-wt/MOZ/0043/2012/G2P[4]
MG926721.1 RVA/Human-wt/MOZ/0308/2012/G2P[4]
KJ752368.1 RVA/Pig-wt/ZAF/MRC-DPRU1533/2007/G2P[4]
KJ752401.1 RVA/Human-wt/ZAF/MRC-DPRU81/2007/G2P[4]
JX965148.1 RVA/Human-wt/AUS/WAPC703/2010/G2P[4]
MG891945.1 RVA/Human-wt/MOZ/0044/2012/G8P[4]
MG926732.1 RVA/Human-wt/MOZ/0314/2012/G8P[4]
MG892011.1 RVA/Human-wt/MOZ/0257/2012/G8P[4]
KJ753807.1 RVA/Human-wt/ZAF/MRC-DPRU2716/2008/G8P[4]P[8]
MG891956.1 RVA/Human-wt/MOZ/0045/2012/G8P[4]
KF636307.1 RVA/Human-wt/ZMB/MRC-DPRU3463/2009/G8P[4]
KX655524.1 RVA/Human-wt/UGA/MUL-13-427/2013/G8P[4]
MG891978.1 RVA/Human-wt/MOZ/0052/2012/G8P[4]
KJ751620.1 RVA/Human-wt/GHA/MRC-DPRU1818/1999/G2P[6]
LC406804.1 RVA/Human-wt/KEN/KDH1968/2014/G3P[6]
KM660122.1 RVA/Human-wt/CMR/MA202/2011/G6P[6]
KP752556.1 RVA/Human-wt/ZAF/MRC-DPRU5594/2011/G2P[4]
MG181883.1 RVA/Human-wt/MWI/BID15I/2012/G2P[6]
JF460841.1 RVA/Human-wt/BEL/F01498/2009/G3P[6]
KJ752179.1 RVA/Human-wt/TGO/MRC-DPRU5164/2010/G3P[6]
KP752489.1 RVA/Human-wt/TGO/MRC-DPRU2206/2009/G3G9P[6]
KJ752988.1 RVA/Human-wt/ZWE/MRC-DPRU1132/XXXX/G2P[4]
RVA/Human-wt/MOZ/HGQ1104/2016/G2P[6]
MT163252.1 RVA/Human-wt/ZAF/UFS-NGS-MRC-DPRU1971/2008/G1P[8]
MT163263.1 RVA/Human-wt/ZAF/UFS-NGS-MRC-DPRU1973/2008/G1P[8]
MH520732.1 RVA/Human-wt/BRA/IAL-R751/2016/G3P[8]
V
IV
AB733137 RVA/Human-tc/JPN/KUN/1980/G2P[4]
III
I
JX406754.1 RVA/Human-tc/USA/Wa/1974/G1P[8]
Outgroup
0.050

NSP5/6-H2

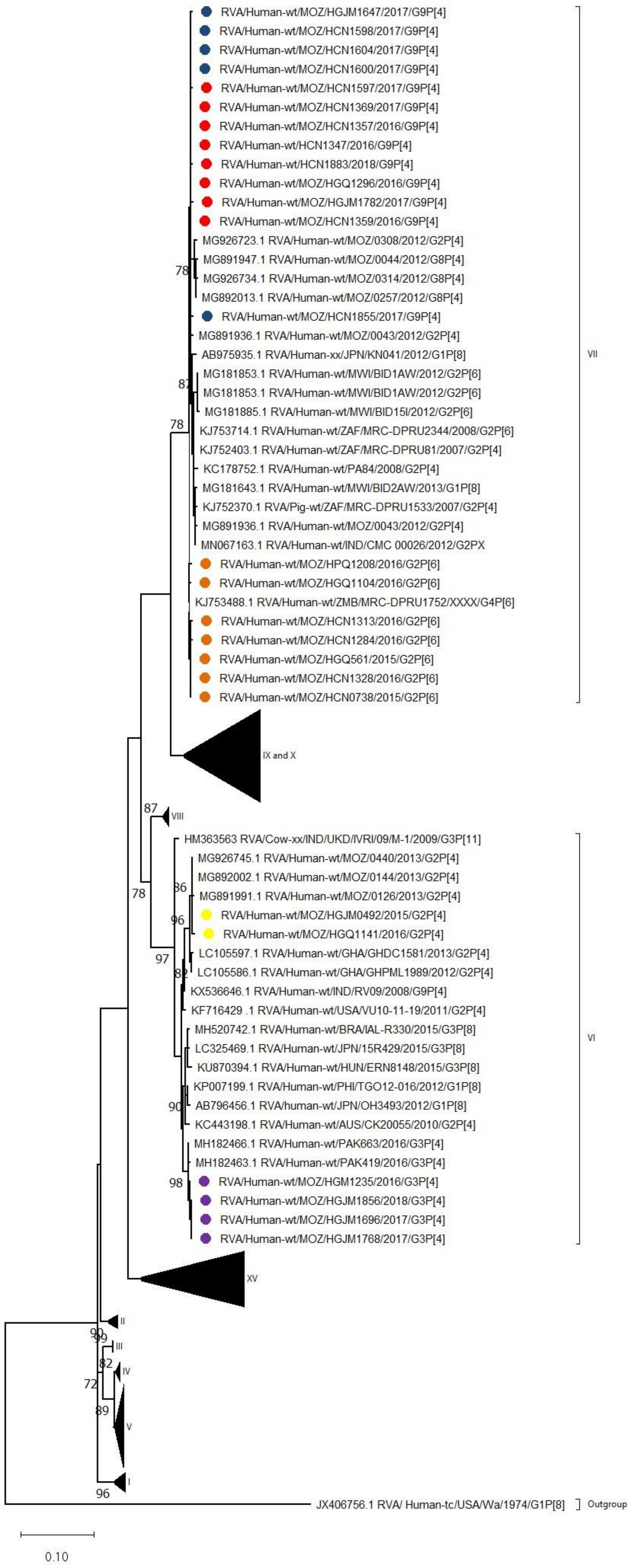

RVA/Human-wt/MOZ/HGJM1647/2017/G9P[4]
RVA/Human-wt/MOZ/HCN1598/2017/G9P[4]
RVA/Human-wt/MOZ/HCN1604/2017/G9P[4]
RVA/Human-wt/MOZ/HCN1600/2017/G9P[4]
RVA/Human-wt/MOZ/HCN1597/2017/G9P[4]
RVA/Human-wt/MOZ/HCN1369/2017/G9P[4]
RVA/Human-wt/MOZ/HCN1357/2016/G9P[4]
RVA/Human-wt/HCN1347/2016/G9P[4]
RVA/Human-wt/HCN1883/2018/G9P[4]
RVA/Human-wt/MOZ/HGQ1296/2016/G9P[4]
RVA/Human-wt/MOZ/HGJM1782/2017/G9P[4]
RVA/Human-wt/MOZ/HCN1359/2016/G9P[4]
MG926723.1 RVA/Human-wt/MOZ/0308/2012/G2P[4]
MG891947.1 RVA/Human-wt/MOZ/0044/2012/G8P[4]
MG926734.1 RVA/Human-wt/MOZ/0314/2012/G8P[4]
MG892013.1 RVA/Human-wt/MOZ/0257/2012/G8P[4]
RVA/Human-wt/MOZ/HCN1855/2017/G9P[4]
MG891936.1 RVA/Human-wt/MOZ/0043/2012/G2P[4]
AB975935.1 RVA/Human-xx/JPN/KN041/2012/G1P[8]
MG181853.1 RVA/Human-wt/MWI/BID1AW/2012/G2P[6]
MG181853.1 RVA/Human-wt/MWI/BID1AW/2012/G2P[6]
MG181885.1 RVA/Human-wt/MWI/BID15I/2012/G2P[6]
KJ753714.1 RVA/Human-wt/ZAF/MRC-DPRU2344/2008/G2P[6]
KJ752403.1 RVA/Human-wt/ZAF/MRC-DPRU81/2007/G2P[4]
KC178752.1 RVA/Human-wt/PA84/2008/G2P[4]
MG181643.1 RVA/Human-wt/MWI/BID2AW/2013/G1P[8]
KJ752370.1 RVA/Pig-wt/ZAF/MRC-DPRU1533/2007/G2P[4]
MG891936.1 RVA/Human-wt/MOZ/0043/2012/G2P[4]
MN067163.1 RVA/Human-wt/IND/CMC 00026/2012/G2PX
RVA/Human-wt/MOZ/HPQ1208/2016/G2P[6]
RVA/Human-wt/MOZ/HGQ1104/2016/G2P[6]
KJ753488.1 RVA/Human-wt/ZMB/MRC-DPRU1752/XXXX/G4P[6]
RVA/Human-wt/MOZ/HCN1313/2016/G2P[6]
RVA/Human-wt/MOZ/HCN1284/2016/G2P[6]
RVA/Human-wt/MOZ/HGQ561/2015/G2P[6]
RVA/Human-wt/MOZ/HCN1328/2016/G2P[6]
RVA/Human-wt/MOZ/HCN0738/2015/G2P[6]
VII
78
87
78
IX and X
87
VIII
HM363563 RVA/Cow-xx/IND/UKD/IVRI/09/M-1/2009/G3P[11]
MG926745.1 RVA/Human-wt/MOZ/0440/2013/G2P[4]
MG892002.1 RVA/Human-wt/MOZ/0144/2013/G2P[4]
MG891991.1 RVA/Human-wt/MOZ/0126/2013/G2P[4]
RVA/Human-wt/MOZ/HGJM0492/2015/G2P[4]
RVA/Human-wt/MOZ/HGQ1141/2016/G2P[4]
LC105597.1 RVA/Human-wt/GHA/GHDC1581/2013/G2P[4]
LC105586.1 RVA/Human-wt/GHA/GHPML1989/2012/G2P[4]
KX536646.1 RVA/Human-wt/IND/RV09/2008/G9P[4]
KF716429 .1 RVA/Human-wt/USA/VU10-11-19/2011/G2P[4]
MH520742.1 RVA/Human-wt/BRA/IAL-R330/2015/G3P[8]
LC325469.1 RVA/Human-wt/JPN/15R429/2015/G3P[8]
KU870394.1 RVA/Human-wt/HUN/ERN8148/2015/G3P[8]
KP007199.1 RVA/Human-wt/PHI/TGO12-016/2012/G1P[8]
AB796456.1 RVA/human-wt/JPN/OH3493/2012/G1P[8]
KC443198.1 RVA/Human-wt/AUS/CK20055/2010/G2P[4]
MH182466.1 RVA/Human-wt/PAK663/2016/G3P[4]
MH182463.1 RVA/Human-wt/PAK419/2016/G3P[4]
RVA/Human-wt/MOZ/HGM1235/2016/G3P[4]
RVA/Human-wt/MOZ/HGJM1856/2018/G3P[4]
RVA/Human-wt/MOZ/HGJM1696/2017/G3P[4]
RVA/Human-wt/MOZ/HGJM1768/2017/G3P[4]
VI
78
86
96
97
82
90
98
XV
II
90
99
III
82
IV
72
89
V
I
96
JX406756.1 RVA/ Human-tc/USA/Wa/1974/G1P[8]
Outgroup
0.10

VP7-G1

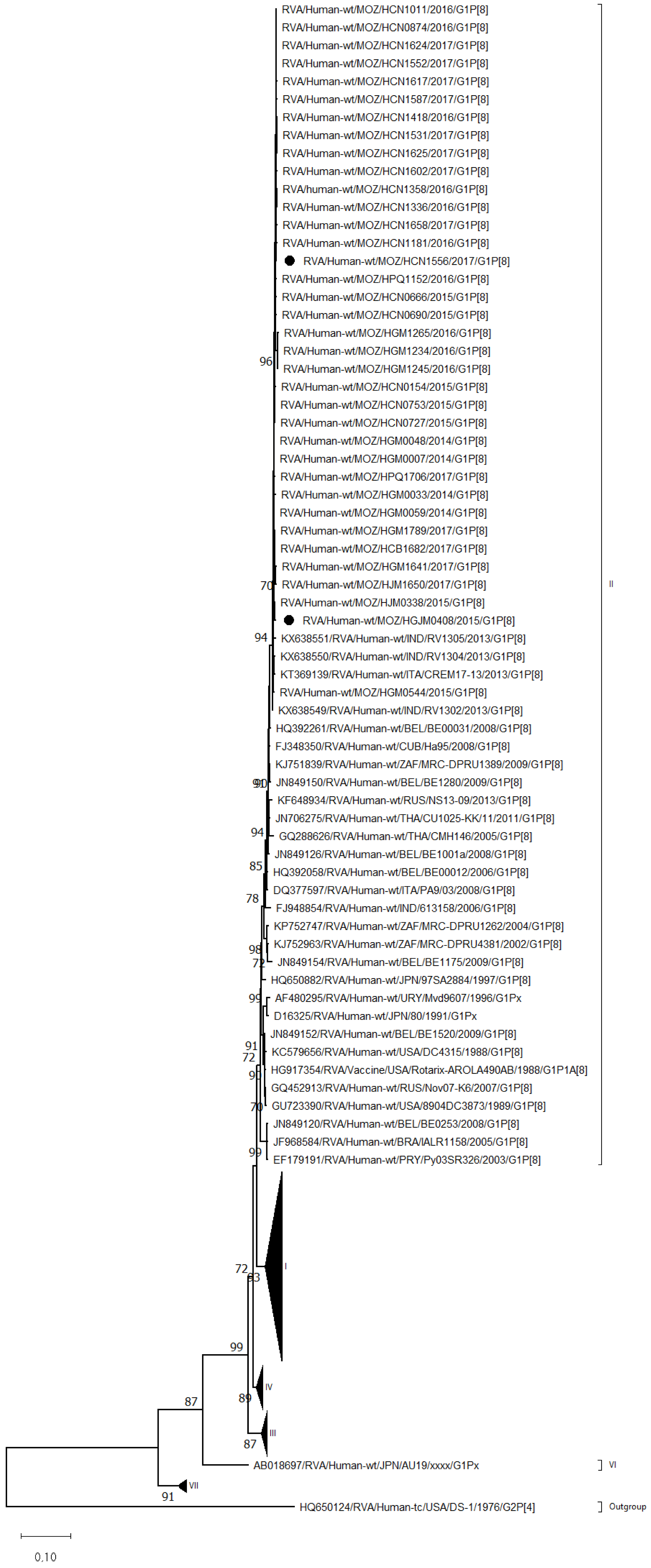
RVA/Human-wt/MOZ/HCN1011/2016/G1P[8]
RVA/Human-wt/MOZ/HCN0874/2016/G1P[8]
RVA/Human-wt/MOZ/HCN1624/2017/G1P[8]
RVA/Human-wt/MOZ/HCN1552/2017/G1P[8]
RVA/Human-wt/MOZ/HCN1617/2017/G1P[8]
RVA/Human-wt/MOZ/HCN1587/2017/G1P[8]
RVA/Human-wt/MOZ/HCN1418/2016/G1P[8]
RVA/Human-wt/MOZ/HCN1531/2017/G1P[8]
RVA/Human-wt/MOZ/HCN1625/2017/G1P[8]
RVA/Human-wt/MOZ/HCN1602/2017/G1P[8]
RVA/human-wt/MOZ/HCN1358/2016/G1P[8]
RVA/Human-wt/MOZ/HCN1336/2016/G1P[8]
RVA/Human-wt/MOZ/HCN1658/2017/G1P[8]
RVA/Human-wt/MOZ/HCN1181/2016/G1P[8]
RVA/Human-wt/MOZ/HCN1556/2017/G1P[8]
RVA/Human-wt/MOZ/HPQ1152/2016/G1P[8]
RVA/Human-wt/MOZ/HCN0666/2015/G1P[8]
RVA/Human-wt/MOZ/HCN0690/2015/G1P[8]
RVA/Human-wt/MOZ/HGM1265/2016/G1P[8]
RVA/Human-wt/MOZ/HGM1234/2016/G1P[8]
96
RVA/Human-wt/MOZ/HGM1245/2016/G1P[8]
RVA/Human-wt/MOZ/HCN0154/2015/G1P[8]
RVA/Human-wt/MOZ/HCN0753/2015/G1P[8]
RVA/Human-wt/MOZ/HCN0727/2015/G1P[8]
RVA/Human-wt/MOZ/HGM0048/2014/G1P[8]
RVA/Human-wt/MOZ/HGM0007/2014/G1P[8]
RVA/Human-wt/MOZ/HPQ1706/2017/G1P[8]
RVA/Human-wt/MOZ/HGM0033/2014/G1P[8]
RVA/Human-wt/MOZ/HGM0059/2014/G1P[8]
RVA/Human-wt/MOZ/HGM1789/2017/G1P[8]
RVA/Human-wt/MOZ/HCB1682/2017/G1P[8]
RVA/Human-wt/MOZ/HGM1641/2017/G1P[8]
70
RVA/Human-wt/MOZ/HJM1650/2017/G1P[8]
II
RVA/Human-wt/MOZ/HJM0338/2015/G1P[8]
RVA/Human-wt/MOZ/HGJM0408/2015/G1P[8]
94
KX638551/RVA/Human-wt/IND/RV1305/2013/G1P[8]
KX638550/RVA/Human-wt/IND/RV1304/2013/G1P[8]
KT369139/RVA/Human-wt/ITA/CREM17-13/2013/G1P[8]
RVA/Human-wt/MOZ/HGM0544/2015/G1P[8]
KX638549/RVA/Human-wt/IND/RV1302/2013/G1P[8]
HQ392261/RVA/Human-wt/BEL/BE00031/2008/G1P[8]
FJ348350/RVA/Human-wt/CUB/Ha95/2008/G1P[8]
KJ751839/RVA/Human-wt/ZAF/MRC-DPRU1389/2009/G1P[8]
JN849150/RVA/Human-wt/BEL/BE1280/2009/G1P[8]
KF648934/RVA/Human-wt/RUS/NS13-09/2013/G1P[8]
JN706275/RVA/Human-wt/THA/CU1025-KK/11/2011/G1P[8]
94
GQ288626/RVA/Human-wt/THA/CMH146/2005/G1P[8]
JN849126/RVA/Human-wt/BEL/BE1001a/2008/G1P[8]
85
HQ392058/RVA/Human-wt/BEL/BE00012/2006/G1P[8]
DQ377597/RVA/Human-wt/ITA/PA9/03/2008/G1P[8]
78
FJ948854/RVA/Human-wt/IND/613158/2006/G1P[8]
KP752747/RVA/Human-wt/ZAF/MRC-DPRU1262/2004/G1P[8]
98
KJ752963/RVA/Human-wt/ZAF/MRC-DPRU4381/2002/G1P[8]
72
JN849154/RVA/Human-wt/BEL/BE1175/2009/G1P[8]
HQ650882/RVA/Human-wt/JPN/97SA2884/1997/G1P[8]
AF480295/RVA/Human-wt/URY/Mvd9607/1996/G1Px
D16325/RVA/Human-wt/JPN/80/1991/G1Px
JN849152/RVA/Human-wt/BEL/BE1520/2009/G1P[8]
91
72
KC579656/RVA/Human-wt/USA/DC4315/1988/G1P[8]
HG917354/RVA/Vaccine/USA/Rotarix-AROLA490AB/1988/G1P1A[8]
GQ452913/RVA/Human-wt/RUS/Nov07-K6/2007/G1P[8]
GU723390/RVA/Human-wt/USA/8904DC3873/1989/G1P[8]
JN849120/RVA/Human-wt/BEL/BE0253/2008/G1P[8]
JF968584/RVA/Human-wt/BRA/IALR1158/2005/G1P[8]
99
EF179191/RVA/Human-wt/PRY/Py03SR326/2003/G1P[8]
72
I
99
IV
87
III
87
AB018697/RVA/Human-wt/JPN/AU19/xxxx/G1Px
VI
VII
91
HQ650124/RVA/Human-tc/USA/DS-1/1976/G2P[4]
Outgroup
0.10

VP6-I1

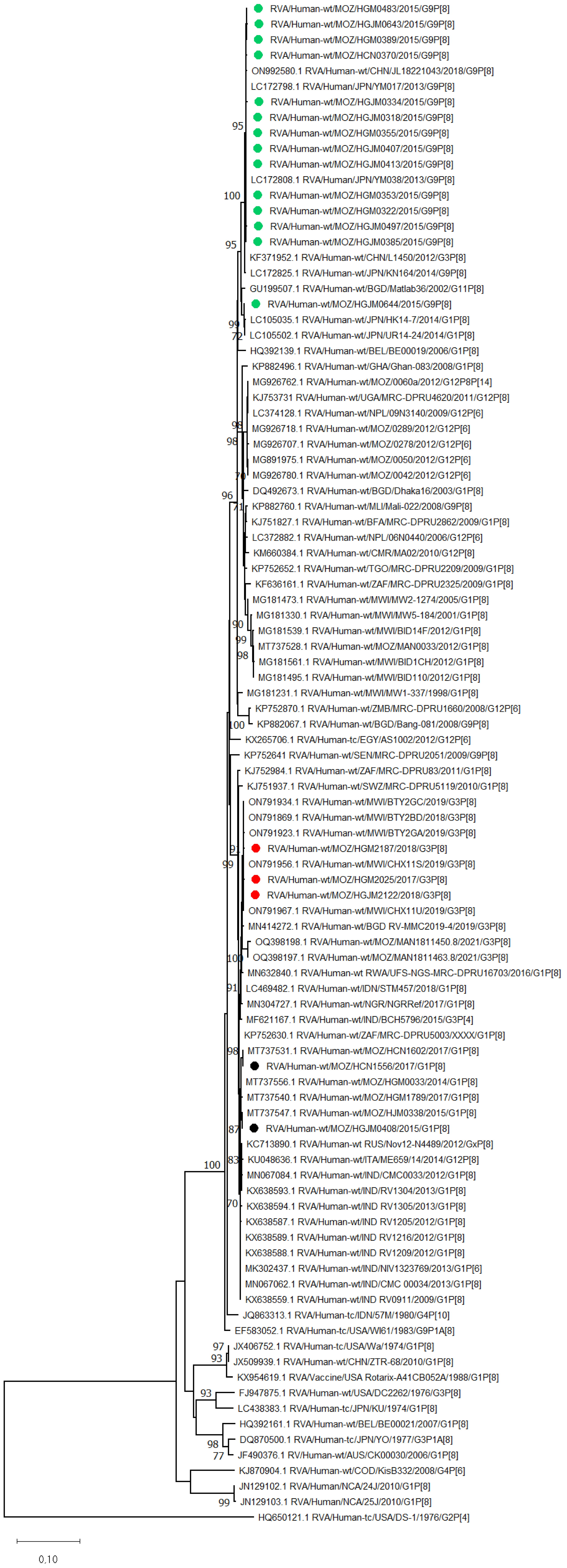
RVA/Human-wt/MOZ/HGM0483/2015/G9P[8]
RVA/Human-wt/MOZ/HGJM0643/2015/G9P[8]
RVA/Human-wt/MOZ/HGM0389/2015/G9P[8]
RVA/Human-wt/MOZ/HCN0370/2015/G9P[8]
ON992580.1 RVA/Human-wt/CHN/JL18221043/2018/G9P[8]
LC172798.1 RVA/Human/JPN/YM017/2013/G9P[8]
RVA/Human-wt/MOZ/HGJM0334/2015/G9P[8]
RVA/Human-wt/MOZ/HGJM0318/2015/G9P[8]
RVA/Human-wt/MOZ/HGM0355/2015/G9P[8]
RVA/Human-wt/MOZ/HGJM0407/2015/G9P[8]
RVA/Human-wt/MOZ/HGJM0413/2015/G9P[8]
LC172808.1 RVA/Human/JPN/YM038/2013/G9P[8]
RVA/Human-wt/MOZ/HGM0353/2015/G9P[8]
RVA/Human-wt/MOZ/HGM0322/2015/G9P[8]
RVA/Human-wt/MOZ/HGJM0497/2015/G9P[8]
RVA/Human-wt/MOZ/HGJM0385/2015/G9P[8]
KF371952.1 RVA/Human-wt/CHN/L1450/2012/G3P[8]
LC172825.1 RVA/Human-wt/JPN/KN164/2014/G9P[8]
GU199507.1 RVA/Human-wt/BGD/Matlab36/2002/G11P[8]
RVA/Human-wt/MOZ/HGJM0644/2015/G9P[8]
LC105035.1 RVA/Human-wt/JPN/HK14-7/2014/G1P[8]
LC105502.1 RVA/Human-wt/JPN/UR14-24/2014/G1P[8]
HQ392139.1 RVA/Human-wt/BEL/BE00019/2006/G1P[8]
KP882496.1 RVA/Human-wt/GHA/Ghan-083/2008/G1P[8]
MG926762.1 RVA/Human-wt/MOZ/0060a/2012/G12P8P[14]
KJ753731 RVA/Human-wt/UGA/MRC-DPRU4620/2011/G12P[8]
LC374128.1 RVA/Human-wt/NPL/09N3140/2009/G12P[6]
MG926718.1 RVA/Human-wt/MOZ/0289/2012/G12P[6]
MG926707.1 RVA/Human-wt/MOZ/0278/2012/G12P[6]
MG891975.1 RVA/Human-wt/MOZ/0050/2012/G12P[6]
MG926780.1 RVA/Human-wt/MOZ/0042/2012/G12P[6]
DQ492673.1 RVA/Human-wt/BGD/Dhaka16/2003/G1P[8]
KP882760.1 RVA/Human-wt/MLI/Mali-022/2008/G9P[8]
KJ751827.1 RVA/Human-wt/BFA/MRC-DPRU2862/2009/G1P[8]
LC372882.1 RVA/Human-wt/NPL/06N0440/2006/G12P[6]
KM660384.1 RVA/Human-wt/CMR/MA02/2010/G12P[8]
KP752652.1 RVA/Human-wt/TGO/MRC-DPRU2209/2009/G1P[8]
KF636161.1 RVA/Human-wt/ZAF/MRC-DPRU2325/2009/G1P[8]
MG181473.1 RVA/Human-wt/MWI/MW2-1274/2005/G1P[8]
MG181330.1 RVA/Human-wt/MWI/MW5-184/2001/G1P[8]
MG181539.1 RVA/Human-wt/MWI/BID14F/2012/G1P[8]
MT737528.1 RVA/Human-wt/MOZ/MAN0033/2012/G1P[8]
MG181561.1 RVA/Human-wt/MWI/BID1CH/2012/G1P[8]
MG181495.1 RVA/Human-wt/MWI/BID110/2012/G1P[8]
MG181231.1 RVA/Human-wt/MWI/MW1-337/1998/G1P[8]
KP752870.1 RVA/Human-wt/ZMB/MRC-DPRU1660/2008/G12P[6]
KP882067.1 RVA/Human-wt/BGD/Bang-081/2008/G9P[8]
KX265706.1 RVA/Human-tc/EGY/AS1002/2012/G12P[6]
KP752641 RVA/Human-wt/SEN/MRC-DPRU2051/2009/G9P[8]
KJ752984.1 RVA/Human-wt/ZAF/MRC-DPRU83/2011/G1P[8]
KJ751937.1 RVA/Human-wt/SWZ/MRC-DPRU5119/2010/G1P[8]
ON791934.1 RVA/Human-wt/MWI/BTY2GC/2019/G3P[8]
ON791869.1 RVA/Human-wt/MWI/BTY2BD/2018/G3P[8]
ON791923.1 RVA/Human-wt/MWI/BTY2GA/2019/G3P[8]
RVA/Human-wt/MOZ/HGM2187/2018/G3P[8]
ON791956.1 RVA/Human-wt/MWI/CHX11S/2019/G3P[8]
RVA/Human-wt/MOZ/HGM2025/2017/G3P[8]
RVA/Human-wt/MOZ/HGJM2122/2018/G3P[8]
ON791967.1 RVA/Human-wt/MWI/CHX11U/2019/G3P[8]
MN414272.1 RVA/Human-wt/BGD RV-MMC2019-4/2019/G3P[8]
OQ398198.1 RVA/Human-wt/MOZ/MAN1811450.8/2021/G3P[8]
OQ398197.1 RVA/Human-wt/MOZ/MAN1811463.8/2021/G3P[8]
MN632840.1 RVA/Human-wt RWA/UFS-NGS-MRC-DPRU16703/2016/G1P[8]
LC469482.1 RVA/Human-wt/IDN/STM457/2018/G1P[8]
MN304727.1 RVA/Human-wt/NGR/NGRRef/2017/G1P[8]
MF621167.1 RVA/Human-wt/IND/BCH5796/2015/G3P[4]
KP752630.1 RVA/Human-wt/ZAF/MRC-DPRU5003/XXXX/G1P[8]
MT737531.1 RVA/Human-wt/MOZ/HCN1602/2017/G1P[8]
RVA/Human-wt/MOZ/HCN1556/2017/G1P[8]
MT737556.1 RVA/Human-wt/MOZ/HGM0033/2014/G1P[8]
MT737540.1 RVA/Human-wt/MOZ/HGM1789/2017/G1P[8]
MT737547.1 RVA/Human-wt/MOZ/HJM0338/2015/G1P[8]
RVA/Human-wt/MOZ/HGJM0408/2015/G1P[8]
KC713890.1 RVA/Human-wt RUS/Nov12-N4489/2012/GxP[8]
KU048636.1 RVA/Human-wt/ITA/ME659/14/2014/G12P[8]
MN067084.1 RVA/Human-wt/IND/CMC0033/2012/G1P[8]
KX638593.1 RVA/Human-wt/IND/RV1304/2013/G1P[8]
KX638594.1 RVA/Human-wt/IND RV1305/2013/G1P[8]
KX638587.1 RVA/Human-wt/IND RV1205/2012/G1P[8]
KX638589.1 RVA/Human-wt/IND RV1216/2012/G1P[8]
KX638588.1 RVA/Human-wt/IND RV1209/2012/G1P[8]
MK302437.1 RVA/Human-wt/IND/NIV1323769/2013/G1P[6]
MN067062.1 RVA/Human-wt/IND/CMC 00034/2013/G1P[8]
KX638559.1 RVA/Human-wt/IND RV0911/2009/G1P[8]
JQ863313.1 RVA/Human-tc/IDN/57M/1980/G4P[10]
EF583052.1 RVA/Human-tc/USA/WI61/1983/G9P1A[8]
JX406752.1 RVA/Human-tc/USA/Wa/1974/G1P[8]
JX509939.1 RVA/Human-wt/CHN/ZTR-68/2010/G1P[8]
KX954619.1 RVA/Vaccine/USA Rotarix-A41CB052A/1988/G1P[8]
FJ947875.1 RVA/Human-wt/USA/DC2262/1976/G3P[8]
LC438383.1 RVA/Human-tc/JPN/KU/1974/G1P[8]
HQ392161.1 RVA/Human-wt/BEL/BE00021/2007/G1P[8]
DQ870500.1 RVA/Human-tc/JPN/YO/1977/G3P1A[8]
JF490376.1 RV/Human-wt/AUS/CK00030/2006/G1P[8]
KJ870904.1 RVA/Human-wt/COD/KisB332/2008/G4P[6]
JN129102.1 RVA/Human/NCA/24J/2010/G1P[8]
JN129103.1 RVA/Human/NCA/25J/2010/G1P[8]
HQ650121.1 RVA/Human-tc/USA/DS-1/1976/G2P[4]
0.10

VP1-R1

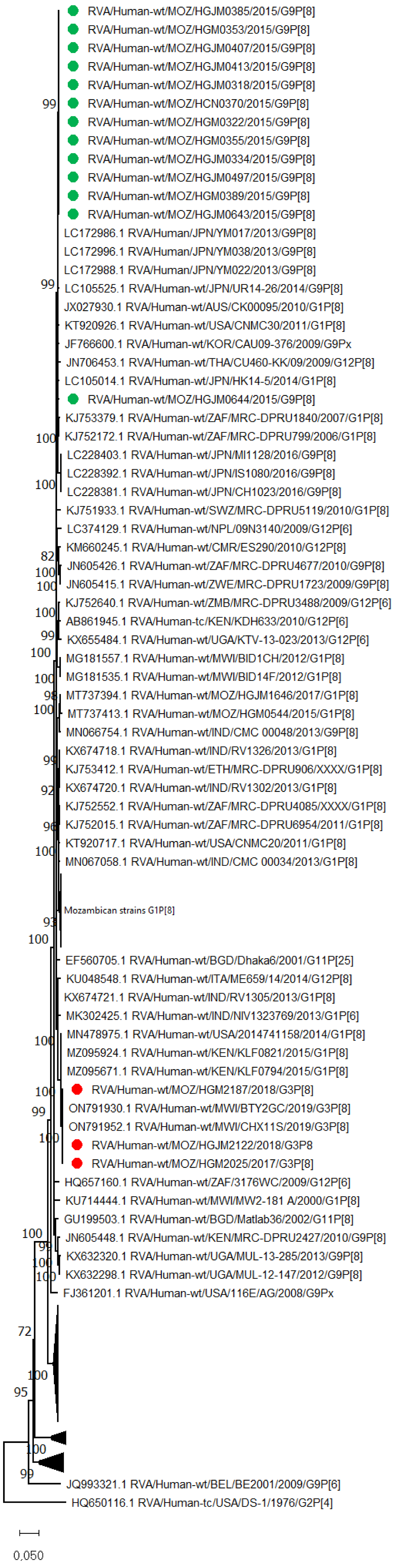

RVA/Human-wt/MOZ/HGJM0385/2015/G9P[8]
RVA/Human-wt/MOZ/HGM0353/2015/G9P[8]
RVA/Human-wt/MOZ/HGJM0407/2015/G9P[8]
RVA/Human-wt/MOZ/HGJM0413/2015/G9P[8]
RVA/Human-wt/MOZ/HGJM0318/2015/G9P[8]
RVA/Human-wt/MOZ/HCN0370/2015/G9P[8]
RVA/Human-wt/MOZ/HGM0322/2015/G9P[8]
RVA/Human-wt/MOZ/HGM0355/2015/G9P[8]
RVA/Human-wt/MOZ/HGJM0334/2015/G9P[8]
RVA/Human-wt/MOZ/HGJM0497/2015/G9P[8]
RVA/Human-wt/MOZ/HGM0389/2015/G9P[8]
RVA/Human-wt/MOZ/HGJM0643/2015/G9P[8]
LC172986.1 RVA/Human/JPN/YM017/2013/G9P[8]
LC172996.1 RVA/Human/JPN/YM038/2013/G9P[8]
LC172988.1 RVA/Human/JPN/YM022/2013/G9P[8]
LC105525.1 RVA/Human-wt/JPN/UR14-26/2014/G9P[8]
JX027930.1 RVA/Human-wt/AUS/CK00095/2010/G1P[8]
KT920926.1 RVA/Human-wt/USA/CNMC30/2011/G1P[8]
JF766600.1 RVA/Human-wt/KOR/CAU09-376/2009/G9Px
JN706453.1 RVA/Human-wt/THA/CU460-KK/09/2009/G12P[8]
LC105014.1 RVA/Human-wt/JPN/HK14-5/2014/G1P[8]
RVA/Human-wt/MOZ/HGJM0644/2015/G9P[8]
KJ753379.1 RVA/Human-wt/ZAF/MRC-DPRU1840/2007/G1P[8]
KJ752172.1 RVA/Human-wt/ZAF/MRC-DPRU799/2006/G1P[8]
LC228403.1 RVA/Human-wt/JPN/MI1128/2016/G9P[8]
LC228392.1 RVA/Human-wt/JPN/IS1080/2016/G9P[8]
LC228381.1 RVA/Human-wt/JPN/CH1023/2016/G9P[8]
KJ751933.1 RVA/Human-wt/SWZ/MRC-DPRU5119/2010/G1P[8]
LC374129.1 RVA/Human-wt/NPL/09N3140/2009/G12P[6]
KM660245.1 RVA/Human-wt/CMR/ES290/2010/G12P[8]
JN605426.1 RVA/Human-wt/ZAF/MRC-DPRU4677/2010/G9P[8]
JN605415.1 RVA/Human-wt/ZWE/MRC-DPRU1723/2009/G9P[8]
KJ752640.1 RVA/Human-wt/ZMB/MRC-DPRU3488/2009/G12P[6]
AB861945.1 RVA/Human-tc/KEN/KDH633/2010/G12P[6]
KX655484.1 RVA/Human-wt/UGA/KTV-13-023/2013/G12P[6]
MG181557.1 RVA/Human-wt/MWI/BID1CH/2012/G1P[8]
MG181535.1 RVA/Human-wt/MWI/BID14F/2012/G1P[8]
MT737394.1 RVA/Human-wt/MOZ/HGJM1646/2017/G1P[8]
MT737413.1 RVA/Human-wt/MOZ/HGM0544/2015/G1P[8]
MN066754.1 RVA/Human-wt/IND/CMC 00048/2013/G9P[8]
KX674718.1 RVA/Human-wt/IND/RV1326/2013/G1P[8]
KJ753412.1 RVA/Human-wt/ETH/MRC-DPRU906/XXXX/G1P[8]
KX674720.1 RVA/Human-wt/IND/RV1302/2013/G1P[8]
KJ752552.1 RVA/Human-wt/ZAF/MRC-DPRU4085/XXXX/G1P[8]
KJ752015.1 RVA/Human-wt/ZAF/MRC-DPRU6954/2011/G1P[8]
KT920717.1 RVA/Human-wt/USA/CNMC20/2011/G1P[8]
MN067058.1 RVA/Human-wt/IND/CMC 00034/2013/G1P[8]
Mozambican strains G1P[8]
EF560705.1 RVA/Human-wt/BGD/Dhaka6/2001/G11P[25]
KU048548.1 RVA/Human-wt/ITA/ME659/14/2014/G12P[8]
KX674721.1 RVA/Human-wt/IND/RV1305/2013/G1P[8]
MK302425.1 RVA/Human-wt/IND/NIV1323769/2013/G1P[6]
MN478975.1 RVA/Human-wt/USA/2014741158/2014/G1P[8]
MZ095924.1 RVA/Human-wt/KEN/KLF0821/2015/G1P[8]
MZ095671.1 RVA/Human-wt/KEN/KLF0794/2015/G1P[8]
RVA/Human-wt/MOZ/HGM2187/2018/G3P[8]
ON791930.1 RVA/Human-wt/MWI/BTY2GC/2019/G3P[8]
ON791952.1 RVA/Human-wt/MWI/CHX11S/2019/G3P[8]
RVA/Human-wt/MOZ/HGJM2122/2018/G3P8
RVA/Human-wt/MOZ/HGM2025/2017/G3P[8]
HQ657160.1 RVA/Human-wt/ZAF/3176WC/2009/G12P[6]
KU714444.1 RVA/Human-wt/MWI/MW2-181 A/2000/G1P[8]
GU199503.1 RVA/Human-wt/BGD/Matlab36/2002/G11P[8]
JN605448.1 RVA/Human-wt/KEN/MRC-DPRU2427/2010/G9P[8]
KX632320.1 RVA/Human-wt/UGA/MUL-13-285/2013/G9P[8]
KX632298.1 RVA/Human-wt/UGA/MUL-12-147/2012/G9P[8]
FJ361201.1 RVA/Human-wt/USA/116E/AG/2008/G9Px
JQ993321.1 RVA/Human-wt/BEL/BE2001/2009/G9P[6]
HQ650116.1 RVA/Human-tc/USA/DS-1/1976/G2P[4]
0,050

VP2-C1

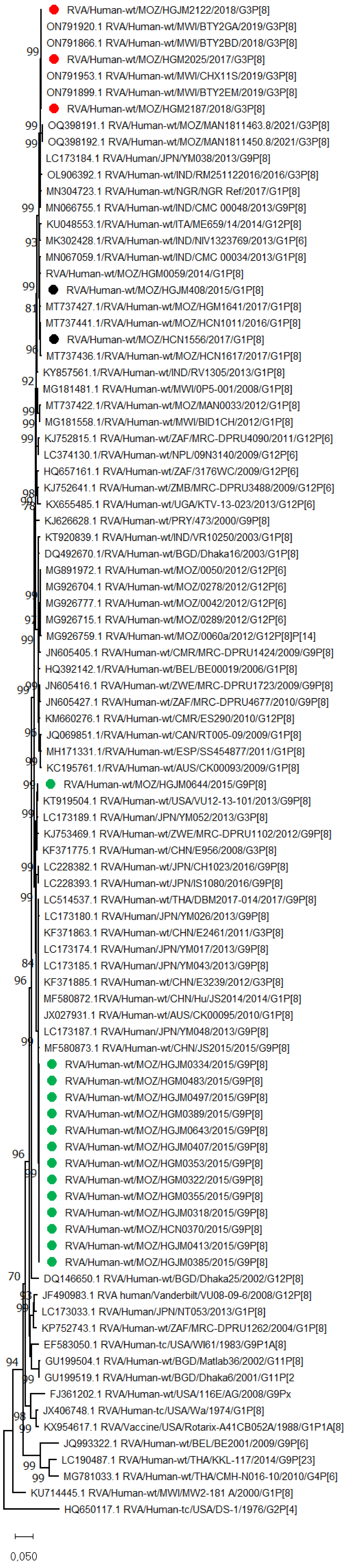
RVA/Human-wt/MOZ/HGJM2122/2018/G3P[8]
ON791920.1 RVA/Human-wt/MWI/BTY2GA/2019/G3P[8]
ON791866.1 RVA/Human-wt/MWI/BTY2BD/2018/G3P[8]
RVA/Human-wt/MOZ/HGM2025/2017/G3P[8]
ON791953.1 RVA/Human-wt/MWI/CHX11S/2019/G3P[8]
ON791899.1 RVA/Human-wt/MWI/BTY2EM/2019/G3P[8]
RVA/Human-wt/MOZ/HGM2187/2018/G3P[8]
OQ398191.1 RVA/Human-wt/MOZ/MAN1811463.8/2021/G3P[8]
OQ398192.1 RVA/Human-wt/MOZ/MAN1811450.8/2021/G3P[8]
LC173184.1 RVA/Human/JPN/YM038/2013/G9P[8]
OL906392.1 RVA/Human-wt/IND/RM251122016/2016/G3P[8]
MN304723.1 RVA/Human-wt/NGR/NGR Ref/2017/G1P[8]
MN066755.1 RVA/Human-wt/IND/CMC 00048/2013/G9P[8]
KU048553.1/RVA/Human-wt/ITA/ME659/14/2014/G12P[8]
MK302428.1/RVA/Human-wt/IND/NIV1323769/2013/G1P[6]
MN067059.1/RVA/Human-wt/IND/CMC 00034/2013/G1P[8]
RVA/Human-wt/MOZ/HGM0059/2014/G1P[8]
RVA/Human-wt/MOZ/HGJM408/2015/G1P[8]
MT737427.1/RVA/Human-wt/MOZ/HGM1641/2017/G1P[8]
MT737441.1/RVA/Human-wt/MOZ/HCN1011/2016/G1P[8]
RVA/Human-wt/MOZ/HCN1556/2017/G1P[8]
MT737436.1/RVA/Human-wt/MOZ/HCN1617/2017/G1P[8]
KY857561.1/RVA/Human-wt/IND/RV1305/2013/G1P[8]
MG181481.1 RVA/Human-wt/MWI/0P5-001/2008/G1P[8]
MT737422.1/RVA/Human-wt/MOZ/MAN0033/2012/G1P[8]
MG181558.1/RVA/Human-wt/MWI/BID1CH/2012/G1P[8]
KJ752815.1 RVA/Human-wt/ZAF/MRC-DPRU4090/2011/G12P[6]
LC374130.1/RVA/Human-wt/NPL/09N3140/2009/G12P[6]
HQ657161.1 RVA/Human-wt/ZAF/3176WC/2009/G12P[6]
KJ752641.1 RVA/Human-wt/ZMB/MRC-DPRU3488/2009/G12P[6]
KX655485.1 RVA/Human-wt/UGA/KTV-13-023/2013/G12P[6]
KJ626628.1 RVA/Human-wt/PRY/473/2000/G9P[8]
KT920839.1 RVA/Human-wt/IND/VR10250/2003/G1P[8]
DQ492670.1/RVA/Human-wt/BGD/Dhaka16/2003/G1P[8]
MG891972.1 RVA/Human-wt/MOZ/0050/2012/G12P[6]
MG926704.1 RVA/Human-wt/MOZ/0278/2012/G12P[6]
MG926777.1 RVA/Human-wt/MOZ/0042/2012/G12P[6]
MG926715.1 RVA/Human-wt/MOZ/0289/2012/G12P[6]
MG926759.1 RVA/Human-wt/MOZ/0060a/2012/G12P[8]P[14]
JN605405.1 RVA/Human-wt/CMR/MRC-DPRU1424/2009/G9P[8]
HQ392142.1/RVA/Human-wt/BEL/BE00019/2006/G1P[8]
JN605416.1 RVA/Human-wt/ZWE/MRC-DPRU1723/2009/G9P[8]
JN605427.1 RVA/Human-wt/ZAF/MRC-DPRU4677/2010/G9P[8]
KM660276.1 RVA/Human-wt/CMR/ES290/2010/G12P[8]
JQ069851.1/RVA/Human-wt/CAN/RT005-09/2009/G1P[8]
MH171331.1/RVA/Human-wt/ESP/SS454877/2011/G1P[8]
KC195761.1/RVA/Human-wt/AUS/CK00093/2009/G1P[8]
RVA/Human-wt/MOZ/HGJM0644/2015/G9P[8]
KT919504.1 RVA/Human-wt/USA/VU12-13-101/2013/G9P[8]
LC173189.1 RVA/Human/JPN/YM052/2013/G3P[8]
KJ753469.1 RVA/Human-wt/ZWE/MRC-DPRU1102/2012/G9P[8]
KF371775.1 RVA/Human-wt/CHN/E956/2008/G3P[8]
LC228382.1 RVA/Human-wt/JPN/CH1023/2016/G9P[8]
LC228393.1 RVA/Human-wt/JPN/IS1080/2016/G9P[8]
LC514537.1 RVA/Human-wt/THA/DBM2017-014/2017/G9P[8]
LC173180.1 RVA/Human/JPN/YM026/2013/G9P[8]
KF371863.1 RVA/Human-wt/CHN/E2461/2011/G3P[8]
LC173174.1 RVA/Human/JPN/YM017/2013/G9P[8]
LC173185.1 RVA/Human/JPN/YM043/2013/G9P[8]
KF371885.1 RVA/Human-wt/CHN/E3239/2012/G3P[8]
MF580872.1RVA/Human-wt/CHN/Hu/JS2014/2014/G1P[8]
JX027931.1 RVA/Human-wt/AUS/CK00095/2010/G1P[8]
LC173187.1 RVA/Human/JPN/YM048/2013/G9P[8]
MF580873.1 RVA/Human-wt/CHN/JS2015/2015/G9P[8]
RVA/Human-wt/MOZ/HGJM0334/2015/G9P[8]
RVA/Human-wt/MOZ/HGM0483/2015/G9P[8]
RVA/Human-wt/MOZ/HGJM0497/2015/G9P[8]
RVA/Human-wt/MOZ/HGM0389/2015/G9P[8]
RVA/Human-wt/MOZ/HGJM0643/2015/G9P[8]
RVA/Human-wt/MOZ/HGJM0407/2015/G9P[8]
RVA/Human-wt/MOZ/HGM0353/2015/G9P[8]
RVA/Human-wt/MOZ/HGM0322/2015/G9P[8]
RVA/Human-wt/MOZ/HGM0355/2015/G9P[8]
RVA/Human-wt/MOZ/HGJM0318/2015/G9P[8]
RVA/Human-wt/MOZ/HCN0370/2015/G9P[8]
RVA/Human-wt/MOZ/HGJM0413/2015/G9P[8]
RVA/Human-wt/MOZ/HGJM0385/2015/G9P[8]
DQ146650.1 RVA/Human-wt/BGD/Dhaka25/2002/G12P[8]
JF490983.1 RVA human/Vanderbilt/VU08-09-6/2008/G12P[8]
LC173033.1 RVA/Human/JPN/NT053/2013/G1P[8]
KP752743.1 RVA/Human-wt/ZAF/MRC-DPRU1262/2004/G1P[8]
EF583050.1 RVA/Human-tc/USA/WI61/1983/G9P1A[8]
GU199504.1 RVA/Human-wt/BGD/Matlab36/2002/G11P[8]
GU199519.1 RVA/Human-wt/BGD/Dhaka6/2001/G11P[2
FJ361202.1 RVA/Human-wt/USA/116E/AG/2008/G9Px
JX406748.1 RVA/Human-tc/USA/Wa/1974/G1P[8]
KX954617.1 RVA/Vaccine/USA/Rotarix-A41CB052A/1988/G1P1A[8]
JQ993322.1 RVA/Human-wt/BEL/BE2001/2009/G9P[6]
LC190487.1 RVA/Human-wt/THA/KKL-117/2014/G9P[23]
MG781033.1 RVA/Human-wt/THA/CMH-N016-10/2010/G4P[6]
KU714445.1 RVA/Human-wt/MWI/MW2-181 A/2000/G1P[8]
HQ650117.1 RVA/Human-tc/USA/DS-1/1976/G2P[4]
0.050

VP3-M1

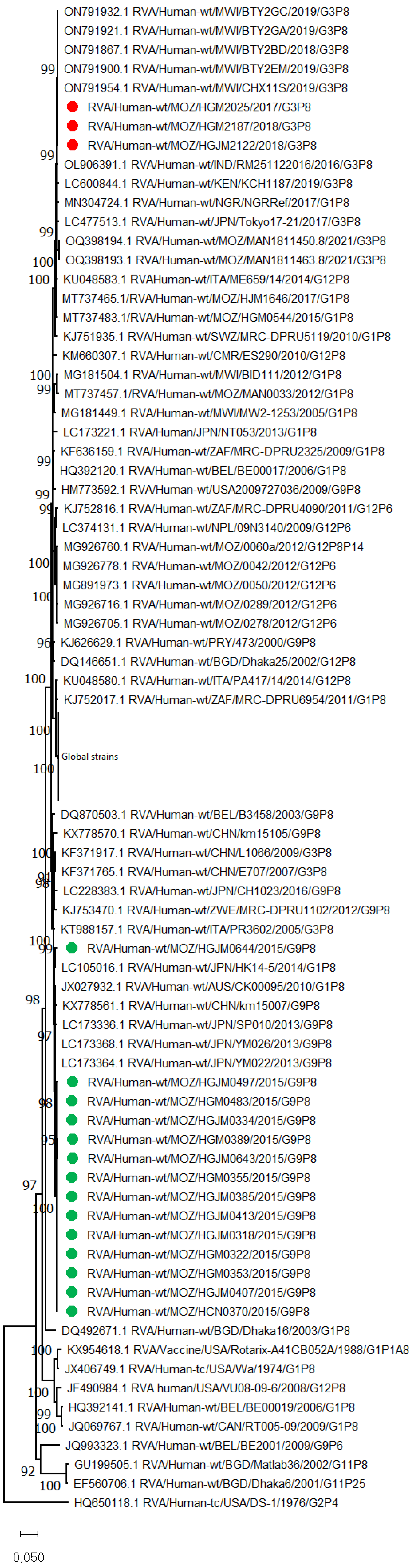
ON791932.1 RVA/Human-wt/MWI/BTY2GC/2019/G3P8
ON791921.1 RVA/Human-wt/MWI/BTY2GA/2019/G3P8
ON791867.1 RVA/Human-wt/MWI/BTY2BD/2018/G3P8
ON791900.1 RVA/Human-wt/MWI/BTY2EM/2019/G3P8
ON791954.1 RVA/Human-wt/MWI/CHX11S/2019/G3P8
RVA/Human-wt/MOZ/HGM2025/2017/G3P8
RVA/Human-wt/MOZ/HGM2187/2018/G3P8
RVA/Human-wt/MOZ/HGJM2122/2018/G3P8
OL906391.1 RVA/Human-wt/IND/RM251122016/2016/G3P8
LC600844.1 RVA/Human-wt/KEN/KCH1187/2019/G3P8
MN304724.1 RVA/Human-wt/NGR/NGRRef/2017/G1P8
LC477513.1 RVA/Human-wt/JPN/Tokyo17-21/2017/G3P8
OQ398194.1 RVA/Human-wt/MOZ/MAN1811450.8/2021/G3P8
OQ398193.1 RVA/Human-wt/MOZ/MAN1811463.8/2021/G3P8
KU048583.1 RVAHuman-wt/ITA/ME659/14/2014/G12P8
MT737465.1/RVA/Human-wt/MOZ/HJM1646/2017/G1P8
MT737483.1/RVA/Human-wt/MOZ/HGM0544/2015/G1P8
KJ751935.1 RVA/Human-wt/SWZ/MRC-DPRU5119/2010/G1P8
KM660307.1 RVA/Human-wt/CMR/ES290/2010/G12P8
MG181504.1 RVA/Human-wt/MWI/BID111/2012/G1P8
MT737457.1/RVA/Human-wt/MOZ/MAN0033/2012/G1P8
MG181449.1 RVA/Human-wt/MWI/MW2-1253/2005/G1P8
LC173221.1 RVA/Human/JPN/NT053/2013/G1P8
KF636159.1 RVA/Human-wt/ZAF/MRC-DPRU2325/2009/G1P8
HQ392120.1 RVA/Human-wt/BEL/BE00017/2006/G1P8
HM773592.1 RVA/Human-wt/USA2009727036/2009/G9P8
KJ752816.1 RVA/Human-wt/ZAF/MRC-DPRU4090/2011/G12P6
LC374131.1 RVA/Human-wt/NPL/09N3140/2009/G12P6
MG926760.1 RVA/Human-wt/MOZ/0060a/2012/G12P8P14
MG926778.1 RVA/Human-wt/MOZ/0042/2012/G12P6
MG891973.1 RVA/Human-wt/MOZ/0050/2012/G12P6
MG926716.1 RVA/Human-wt/MOZ/0289/2012/G12P6
MG926705.1 RVA/Human-wt/MOZ/0278/2012/G12P6
KJ626629.1 RVA/Human-wt/PRY/473/2000/G9P8
DQ146651.1 RVA/Human-wt/BGD/Dhaka25/2002/G12P8
KU048580.1 RVA/Human-wt/ITA/PA417/14/2014/G12P8
KJ752017.1 RVA/Human-wt/ZAF/MRC-DPRU6954/2011/G1P8
Global strains
DQ870503.1 RVA/Human-wt/BEL/B3458/2003/G9P8
KX778570.1 RVA/Human-wt/CHN/km15105/G9P8
KF371917.1 RVA/Human-wt/CHN/L1066/2009/G3P8
KF371765.1 RVA/Human-wt/CHN/E707/2007/G3P8
LC228383.1 RVA/Human-wt/JPN/CH1023/2016/G9P8
KJ753470.1 RVA/Human-wt/ZWE/MRC-DPRU1102/2012/G9P8
KT988157.1 RVA/Human-wt/ITA/PR3602/2005/G3P8
RVA/Human-wt/MOZ/HGJM0644/2015/G9P8
LC105016.1 RVA/Human-wt/JPN/HK14-5/2014/G1P8
JX027932.1 RVA/Human-wt/AUS/CK00095/2010/G1P8
KX778561.1 RVA/Human-wt/CHN/km15007/G9P8
LC173336.1 RVA/Human-wt/JPN/SP010/2013/G9P8
LC173368.1 RVA/Human-wt/JPN/YM026/2013/G9P8
LC173364.1 RVA/Human-wt/JPN/YM022/2013/G9P8
RVA/Human-wt/MOZ/HGJM0497/2015/G9P8
RVA/Human-wt/MOZ/HGM0483/2015/G9P8
RVA/Human-wt/MOZ/HGJM0334/2015/G9P8
RVA/Human-wt/MOZ/HGM0389/2015/G9P8
RVA/Human-wt/MOZ/HGJM0643/2015/G9P8
RVA/Human-wt/MOZ/HGM0355/2015/G9P8
RVA/Human-wt/MOZ/HGJM0385/2015/G9P8
RVA/Human-wt/MOZ/HGJM0413/2015/G9P8
RVA/Human-wt/MOZ/HGJM0318/2015/G9P8
RVA/Human-wt/MOZ/HGM0322/2015/G9P8
RVA/Human-wt/MOZ/HGM0353/2015/G9P8
RVA/Human-wt/MOZ/HGJM0407/2015/G9P8
RVA/Human-wt/MOZ/HCN0370/2015/G9P8
DQ492671.1 RVA/Human-wt/BGD/Dhaka16/2003/G1P8
KX954618.1 RVA/Vaccine/USA/Rotarix-A41CB052A/1988/G1P1A8
JX406749.1 RVA/Human-tc/USA/Wa/1974/G1P8
JF490984.1 RVA human/USA/VU08-09-6/2008/G12P8
HQ392141.1 RVA/Human-wt/BEL/BE00019/2006/G1P8
JQ069767.1 RVA/Human-wt/CAN/RT005-09/2009/G1P8
JQ993323.1 RVA/Human-wt/BEL/BE2001/2009/G9P6
GU199505.1 RVA/Human-wt/BGD/Matlab36/2002/G11P8
EF560706.1 RVA/Human-wt/BGD/Dhaka6/2001/G11P25
HQ650118.1 RVA/Human-tc/USA/DS-1/1976/G2P4
0.050

NSP1-A1

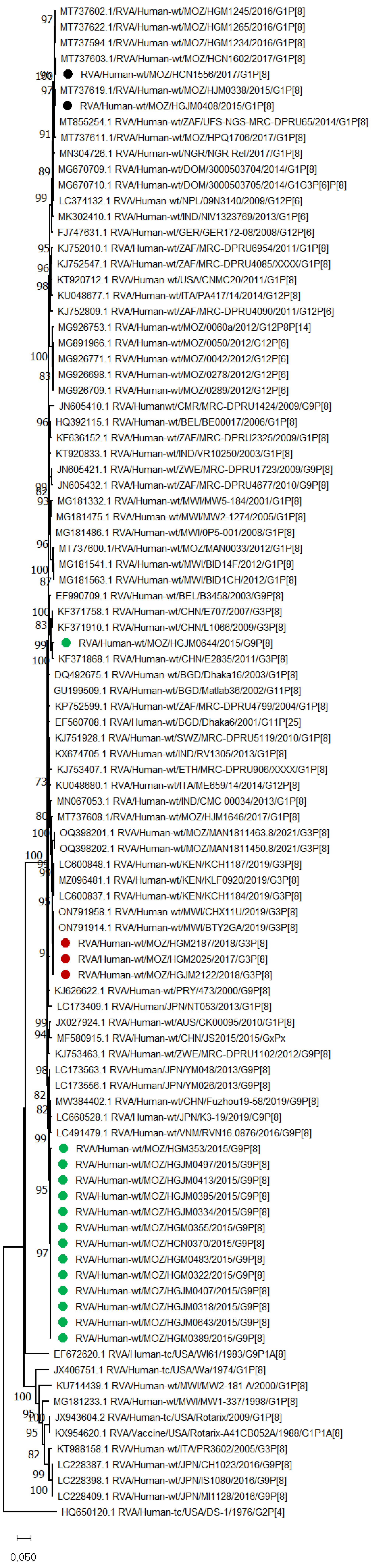

MT737602.1/RVA/Human-wt/MOZ/HGM1245/2016/G1P[8]
MT737622.1/RVA/Human-wt/MOZ/HGM1265/2016/G1P[8]
MT737594.1/RVA/Human-wt/MOZ/HGM1234/2016/G1P[8]
MT737603.1/RVA/Human-wt/MOZ/HCN1602/2017/G1P[8]
RVA/Human-wt/MOZ/HCN1556/2017/G1P[8]
MT737619.1/RVA/Human-wt/MOZ/HJM0338/2015/G1P[8]
RVA/Human-wt/MOZ/HGJM0408/2015/G1P[8]
MT855254.1 RVA/Human-wt/ZAF/UFS-NGS-MRC-DPRU65/2014/G1P[8]
MT737611.1/RVA/Human-wt/MOZ/HPQ1706/2017/G1P[8]
MN304726.1 RVA/Human-wt/NGR/NGR Ref/2017/G1P[8]
MG670709.1 RVA/Human-wt/DOM/3000503704/2014/G1P[8]
MG670710.1 RVA/Human-wt/DOM/3000503705/2014/G1G3P[6]P[8]
LC374132.1 RVA/Human-wt/NPL/09N3140/2009/G12P[6]
MK302410.1 RVA/Human-wt/IND/NIV1323769/2013/G1P[6]
FJ747631.1 RVA/Human-wt/GER/GER172-08/2008/G12P[6]
KJ752010.1 RVA/Human-wt/ZAF/MRC-DPRU6954/2011/G1P[8]
KJ752547.1 RVA/Human-wt/ZAF/MRC-DPRU4085/XXXX/G1P[8]
KT920712.1 RVA/Human-wt/USA/CNMC20/2011/G1P[8]
KU048677.1 RVA/Human-wt/ITA/PA417/14/2014/G12P[8]
KJ752809.1 RVA/Human-wt/ZAF/MRC-DPRU4090/2011/G12P[6]
MG926753.1 RVA/Human-wt/MOZ/0060a/2012/G12P8P[14]
MG891966.1 RVA/Human-wt/MOZ/0050/2012/G12P[6]
MG926771.1 RVA/Human-wt/MOZ/0042/2012/G12P[6]
MG926698.1 RVA/Human-wt/MOZ/0278/2012/G12P[6]
MG926709.1 RVA/Human-wt/MOZ/0289/2012/G12P[6]
JN605410.1 RVA/Humanwt/CMR/MRC-DPRU1424/2009/G9P[8]
HQ392115.1 RVA/Human-wt/BEL/BE00017/2006/G1P[8]
KF636152.1 RVA/Human-wt/ZAF/MRC-DPRU2325/2009/G1P[8]
KT920833.1 RVA/Human-wt/IND/VR10250/2003/G1P[8]
JN605421.1 RVA/Human-wt/ZWE/MRC-DPRU1723/2009/G9P[8]
JN605432.1 RVA/Human-wt/ZAF/MRC-DPRU4677/2010/G9P[8]
MG181332.1 RVA/Human-wt/MWI/MW5-184/2001/G1P[8]
MG181475.1 RVA/Human-wt/MWI/MW2-1274/2005/G1P[8]
MG181486.1 RVA/Human-wt/MWI/0P5-001/2008/G1P[8]
MT737600.1/RVA/Human-wt/MOZ/MAN0033/2012/G1P[8]
MG181541.1 RVA/Human-wt/MWI/BID14F/2012/G1P[8]
MG181563.1 RVA/Human-wt/MWI/BID1CH/2012/G1P[8]
EF990709.1 RVA/Human-wt/BEL/B3458/2003/G9P[8]
KF371758.1 RVA/Human-wt/CHN/E707/2007/G3P[8]
KF371910.1 RVA/Human-wt/CHN/L1066/2009/G3P[8]
RVA/Human-wt/MOZ/HGJM0644/2015/G9P[8]
KF371868.1 RVA/Human-wt/CHN/E2835/2011/G3P[8]
DQ492675.1 RVA/Human-wt/BGD/Dhaka16/2003/G1P[8]
GU199509.1 RVA/Human-wt/BGD/Matlab36/2002/G11P[8]
KP752599.1 RVA/Human-wt/ZAF/MRC-DPRU4799/2004/G1P[8]
EF560708.1 RVA/Human-wt/BGD/Dhaka6/2001/G11P[25]
KJ751928.1 RVA/Human-wt/SWZ/MRC-DPRU5119/2010/G1P[8]
KX674705.1 RVA/Human-wt/IND/RV1305/2013/G1P[8]
KJ753407.1 RVA/Human-wt/ETH/MRC-DPRU906/XXXX/G1P[8]
KU048680.1 RVA/Human-wt/ITA/ME659/14/2014/G12P[8]
MN067053.1 RVA/Human-wt/IND/CMC 00034/2013/G1P[8]
MT737608.1/RVA/Human-wt/MOZ/HJM1646/2017/G1P[8]
OQ398201.1 RVA/Human-wt/MOZ/MAN1811463.8/2021/G3P[8]
OQ398202.1 RVA/Human-wt/MOZ/MAN1811450.8/2021/G3P[8]
LC600848.1 RVA/Human-wt/KEN/KCH1187/2019/G3P[8]
MZ096481.1 RVA/Human-wt/KEN/KLF0920/2019/G3P[8]
LC600837.1 RVA/Human-wt/KEN/KCH1184/2019/G3P[8]
ON791958.1 RVA/Human-wt/MWI/CHX11U/2019/G3P[8]
ON791914.1 RVA/Human-wt/MWI/BTY2GA/2019/G3P[8]
RVA/Human-wt/MOZ/HGM2187/2018/G3P[8]
RVA/Human-wt/MOZ/HGM2025/2017/G3P[8]
RVA/Human-wt/MOZ/HGJM2122/2018/G3P[8]
KJ626622.1 RVA/Human-wt/PRY/473/2000/G9P[8]
LC173409.1 RVA/Human/JPN/NT053/2013/G1P[8]
JX027924.1 RVA/Human-wt/AUS/CK00095/2010/G1P[8]
MF580915.1 RVA/Human-wt/CHN/JS2015/2015/GxPx
KJ753463.1 RVA/Human-wt/ZWE/MRC-DPRU1102/2012/G9P[8]
LC173563.1 RVA/Human/JPN/YM048/2013/G9P[8]
LC173556.1 RVA/Human/JPN/YM026/2013/G9P[8]
MW384402.1 RVA/Human-wt/CHN/Fuzhou19-58/2019/G9P[8]
LC668528.1 RVA/Human-wt/JPN/K3-19/2019/G9P[8]
LC491479.1 RVA/Human-wt/VNM/RVN16.0876/2016/G9P[8]
RVA/Human-wt/MOZ/HGM353/2015/G9P[8]
RVA/Human-wt/MOZ/HGJM0497/2015/G9P[8]
RVA/Human-wt/MOZ/HGJM0413/2015/G9P[8]
RVA/Human-wt/MOZ/HGJM0385/2015/G9P[8]
RVA/Human-wt/MOZ/HGJM0334/2015/G9P[8]
RVA/Human-wt/MOZ/HGM0355/2015/G9P[8]
RVA/Human-wt/MOZ/HCN0370/2015/G9P[8]
RVA/Human-wt/MOZ/HGM0483/2015/G9P[8]
RVA/Human-wt/MOZ/HGM0322/2015/G9P[8]
RVA/Human-wt/MOZ/HGJM0407/2015/G9P[8]
RVA/Human-wt/MOZ/HGJM0318/2015/G9P[8]
RVA/Human-wt/MOZ/HGJM0643/2015/G9P[8]
RVA/Human-wt/MOZ/HGM0389/2015/G9P[8]
EF672620.1 RVA/Human-tc/USA/WI61/1983/G9P1A[8]
JX406751.1 RVA/Human-tc/USA/Wa/1974/G1P[8]
KU714439.1 RVA/Human-wt/MWI/MW2-181 A/2000/G1P[8]
MG181233.1 RVA/Human-wt/MWI/MW1-337/1998/G1P[8]
JX943604.2 RVA/Human-tc/USA/Rotarix/2009/G1P[8]
KX954620.1 RVA/Vaccine/USA/Rotarix-A41CB052A/1988/G1P1A[8]
KT988158.1 RVA/Human-wt/ITA/PR3602/2005/G3P[8]
LC228387.1 RVA/Human-wt/JPN/CH1023/2016/G9P[8]
LC228398.1 RVA/Human-wt/JPN/IS1080/2016/G9P[8]
LC228409.1 RVA/Human-wt/JPN/MI1128/2016/G9P[8]
HQ650120.1 RVA/Human-tc/USA/DS-1/1976/G2P[4]
0.050

NSP2-N1

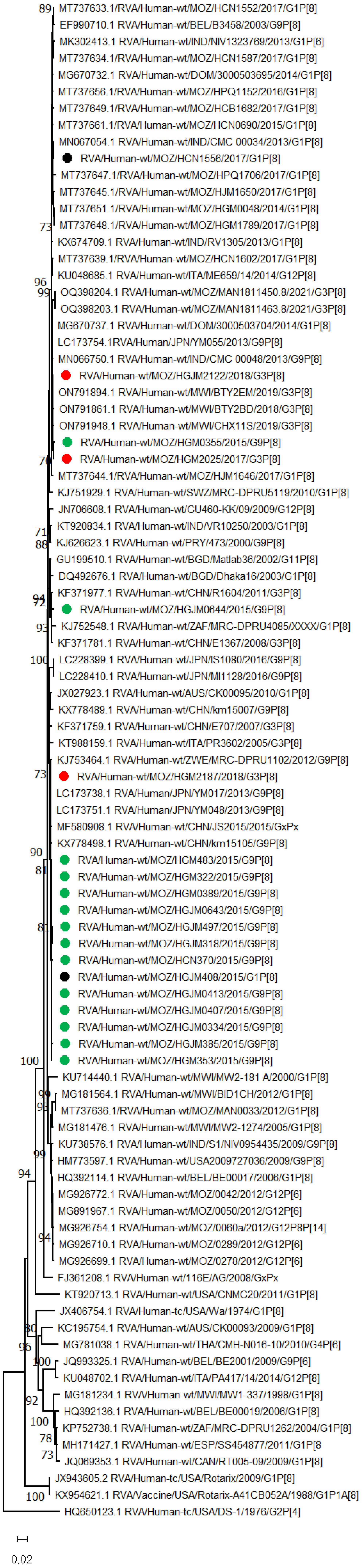

MT737633.1/RVA/Human-wt/MOZ/HCN1552/2017/G1P[8]
EF990710.1 RVA/Human-wt/BEL/B3458/2003/G9P[8]
MK302413.1 RVA/Human-wt/IND/NIV1323769/2013/G1P[6]
MT737634.1/RVA/Human-wt/MOZ/HCN1587/2017/G1P[8]
MG670732.1 RVA/Human-wt/DOM/3000503695/2014/G1P[8]
MT737656.1/RVA/Human-wt/MOZ/HPQ1152/2016/G1P[8]
MT737649.1/RVA/Human-wt/MOZ/HCB1682/2017/G1P[8]
MT737661.1/RVA/Human-wt/MOZ/HCN0690/2015/G1P[8]
MN067054.1 RVA/Human-wt/IND/CMC 00034/2013/G1P[8]
RVA/Human-wt/MOZ/HCN1556/2017/G1P[8]
MT737647.1/RVA/Human-wt/MOZ/HPQ1706/2017/G1P[8]
MT737645.1/RVA/Human-wt/MOZ/HJM1650/2017/G1P[8]
MT737651.1/RVA/Human-wt/MOZ/HGM0048/2014/G1P[8]
MT737648.1/RVA/Human-wt/MOZ/HGM1789/2017/G1P[8]
KX674709.1 RVA/Human-wt/IND/RV1305/2013/G1P[8]
MT737639.1/RVA/Human-wt/MOZ/HCN1602/2017/G1P[8]
KU048685.1 RVA/Human-wt/ITA/ME659/14/2014/G12P[8]
OQ398204.1 RVA/Human-wt/MOZ/MAN1811450.8/2021/G3P[8]
OQ398203.1 RVA/Human-wt/MOZ/MAN1811463.8/2021/G3P[8]
MG670737.1 RVA/Human-wt/DOM/3000503704/2014/G1P[8]
LC173754.1RVA/Human/JPN/YM055/2013/G9P[8]
MN066750.1 RVA/Human-wt/IND/CMC 00048/2013/G9P[8]
RVA/Human-wt/MOZ/HGJM2122/2018/G3P[8]
ON791894.1 RVA/Human-wt/MWI/BTY2EM/2019/G3P[8]
ON791861.1 RVA/Human-wt/MWI/BTY2BD/2018/G3P[8]
ON791948.1 RVA/Human-wt/MWI/CHX11S/2019/G3P[8]
RVA/Human-wt/MOZ/HGM0355/2015/G9P[8]
RVA/Human-wt/MOZ/HGM2025/2017/G3P[8]
MT737644.1/RVA/Human-wt/MOZ/HJM1646/2017/G1P[8]
KJ751929.1 RVA/Human-wt/SWZ/MRC-DPRU5119/2010/G1P[8]
JN706608.1 RVA/Human-wt/CU460-KK/09/2009/G12P[8]
KT920834.1 RVA/Human-wt/IND/VR10250/2003/G1P[8]
KJ626623.1 RVA/Human-wt/PRY/473/2000/G9P[8]
GU199510.1 RVA/Human-wt/BGD/Matlab36/2002/G11P[8]
DQ492676.1 RVA/Human-wt/BGD/Dhaka16/2003/G1P[8]
KF371977.1 RVA/Human-wt/CHN/R1604/2011/G3P[8]
RVA/Human-wt/MOZ/HGJM0644/2015/G9P[8]
KJ752548.1 RVA/Human-wt/ZAF/MRC-DPRU4085/XXXX/G1P[8]
KF371781.1 RVA/Human-wt/CHN/E1367/2008/G3P[8]
LC228399.1 RVA/Human-wt/JPN/IS1080/2016/G9P[8]
LC228410.1 RVA/Human-wt/JPN/MI1128/2016/G9P[8]
JX027923.1 RVA/Human-wt/AUS/CK00095/2010/G1P[8]
KX778489.1 RVA/Human-wt/CHN/km15007/G9P[8]
KF371759.1 RVA/Human-wt/CHN/E707/2007/G3P[8]
KT988159.1 RVA/Human-wt/ITA/PR3602/2005/G3P[8]
KJ753464.1 RVA/Human-wt/ZWE/MRC-DPRU1102/2012/G9P[8]
RVA/Human-wt/MOZ/HGM2187/2018/G3P[8]
LC173738.1 RVA/Human/JPN/YM017/2013/G9P[8]
LC173751.1 RVA/Human/JPN/YM048/2013/G9P[8]
MF580908.1 RVA/Human-wt/CHN/JS2015/2015/GxPx
KX778498.1 RVA/Human-wt/CHN/km15105/G9P[8]
RVA/Human-wt/MOZ/HGM483/2015/G9P[8]
RVA/Human-wt/MOZ/HGM322/2015/G9P[8]
RVA/Human-wt/MOZ/HGM0389/2015/G9P[8]
RVA/Human-wt/MOZ/HGJM0643/2015/G9P[8]
RVA/Human-wt/MOZ/HGJM497/2015/G9P[8]
RVA/Human-wt/MOZ/HGJM318/2015/G9P[8]
RVA/Human-wt/MOZ/HCN370/2015/G9P[8]
RVA/Human-wt/MOZ/HGJM408/2015/G1P[8]
RVA/Human-wt/MOZ/HGJM0413/2015/G9P[8]
RVA/Human-wt/MOZ/HGJM0407/2015/G9P[8]
RVA/Human-wt/MOZ/HGJM0334/2015/G9P[8]
RVA/Human-wt/MOZ/HGJM385/2015/G9P[8]
RVA/Human-wt/MOZ/HGM353/2015/G9P[8]
KU714440.1 RVA/Human-wt/MWI/MW2-181 A/2000/G1P[8]
MG181564.1 RVA/Human-wt/MWI/BID1CH/2012/G1P[8]
MT737636.1/RVA/Human-wt/MOZ/MAN0033/2012/G1P[8]
MG181476.1 RVA/Human-wt/MWI/MW2-1274/2005/G1P[8]
KU738576.1 RVA/Human-wt/IND/S1/NIV0954435/2009/G9P[8]
HM773597.1 RVA/Human-wt/USA2009727036/2009/G9P[8]
HQ392114.1 RVA/Human-wt/BEL/BE00017/2006/G1P[8]
MG926772.1 RVA/Human-wt/MOZ/0042/2012/G12P[6]
MG891967.1 RVA/Human-wt/MOZ/0050/2012/G12P[6]
MG926754.1 RVA/Human-wt/MOZ/0060a/2012/G12P8P[14]
MG926710.1 RVA/Human-wt/MOZ/0289/2012/G12P[6]
MG926699.1 RVA/Human-wt/MOZ/0278/2012/G12P[6]
FJ361208.1 RVA/Human-wt/116E/AG/2008/GxPx
KT920713.1 RVA/Human-wt/USA/CNMC20/2011/G1P[8]
JX406754.1 RVA/Human-tc/USA/Wa/1974/G1P[8]
KC195754.1 RVA/Human-wt/AUS/CK00093/2009/G1P[8]
MG781038.1 RVA/Human-wt/THA/CMH-N016-10/2010/G4P[6]
JQ993325.1 RVA/Human-wt/BEL/BE2001/2009/G9P[6]
KU048702.1 RVA/Human-wt/ITA/PA417/14/2014/G12P[8]
MG181234.1 RVA/Human-wt/MWI/MW1-337/1998/G1P[8]
HQ392136.1 RVA/Human-wt/BEL/BE00019/2006/G1P[8]
KP752738.1 RVA/Human-wt/ZAF/MRC-DPRU1262/2004/G1P[8]
MH171427.1 RVA/Human-wt/ESP/SS454877/2011/G1P[8
JQ069353.1 RVA/Human-wt/CAN/RT005-09/2009/G1P[8]
JX943605.2 RVA/Human-tc/USA/Rotarix/2009/G1P[8]
KX954621.1 RVA/Vaccine/USA/Rotarix-A41CB052A/1988/G1P1A[8]
HQ650123.1 RVA/Human-tc/USA/DS-1/1976/G2P[4]
0.02

NSP3-T1

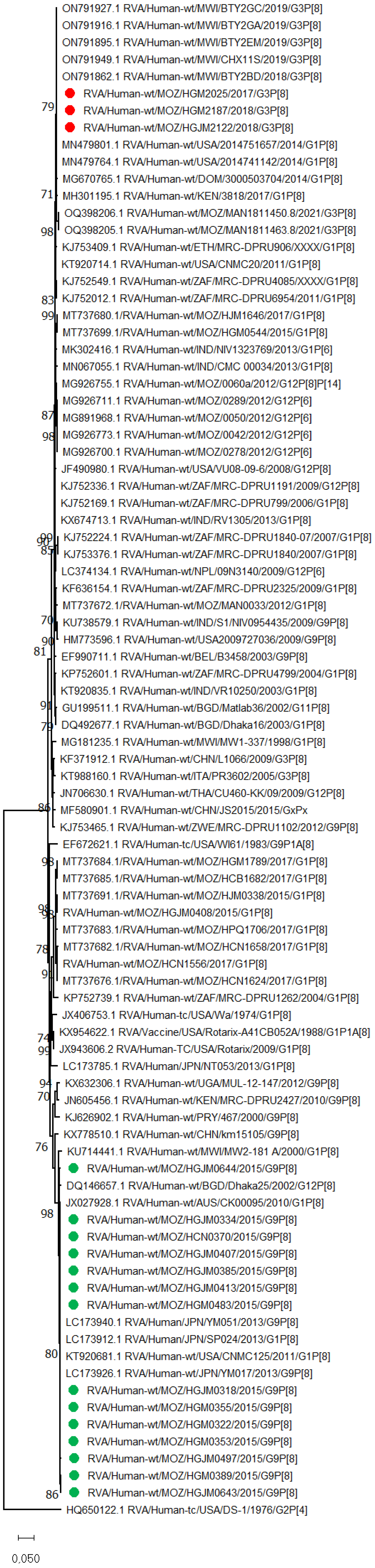
ON791927.1 RVA/Human-wt/MWI/BTY2GC/2019/G3P[8]
ON791916.1 RVA/Human-wt/MWI/BTY2GA/2019/G3P[8]
ON791895.1 RVA/Human-wt/MWI/BTY2EM/2019/G3P[8]
ON791949.1 RVA/Human-wt/MWI/CHX11S/2019/G3P[8]
ON791862.1 RVA/Human-wt/MWI/BTY2BD/2018/G3P[8]
RVA/Human-wt/MOZ/HGM2025/2017/G3P[8]
RVA/Human-wt/MOZ/HGM2187/2018/G3P[8]
RVA/Human-wt/MOZ/HGJM2122/2018/G3P[8]
MN479801.1 RVA/Human-wt/USA/2014751657/2014/G1P[8]
MN479764.1 RVA/Human-wt/USA/2014741142/2014/G1P[8]
MG670765.1 RVA/Human-wt/DOM/3000503704/2014/G1P[8]
MH301195.1 RVA/Human-wt/KEN/3818/2017/G1P[8]
OQ398206.1 RVA/Human-wt/MOZ/MAN1811450.8/2021/G3P[8]
OQ398205.1 RVA/Human-wt/MOZ/MAN1811463.8/2021/G3P[8]
KJ753409.1 RVA/Human-wt/ETH/MRC-DPRU906/XXXX/G1P[8]
KT920714.1 RVA/Human-wt/USA/CNMC20/2011/G1P[8]
KJ752549.1 RVA/Human-wt/ZAF/MRC-DPRU4085/XXXX/G1P[8]
KJ752012.1 RVA/Human-wt/ZAF/MRC-DPRU6954/2011/G1P[8]
MT737680.1/RVA/Human-wt/MOZ/HJM1646/2017/G1P[8]
MT737699.1/RVA/Human-wt/MOZ/HGM0544/2015/G1P[8]
MK302416.1 RVA/Human-wt/IND/NIV1323769/2013/G1P[6]
MN067055.1 RVA/Human-wt/IND/CMC 00034/2013/G1P[8]
MG926755.1 RVA/Human-wt/MOZ/0060a/2012/G12P[8]P[14]
MG926711.1 RVA/Human-wt/MOZ/0289/2012/G12P[6]
MG891968.1 RVA/Human-wt/MOZ/0050/2012/G12P[6]
MG926773.1 RVA/Human-wt/MOZ/0042/2012/G12P[6]
MG926700.1 RVA/Human-wt/MOZ/0278/2012/G12P[6]
JF490980.1 RVA/Human-wt/USA/VU08-09-6/2008/G12P[8]
KJ752336.1 RVA/Human-wt/ZAF/MRC-DPRU1191/2009/G12P[8]
KJ752169.1 RVA/Human-wt/ZAF/MRC-DPRU799/2006/G1P[8]
KX674713.1 RVA/Human-wt/IND/RV1305/2013/G1P[8]
KJ752224.1 RVA/Human-wt/ZAF/MRC-DPRU1840-07/2007/G1P[8]
KJ753376.1 RVA/Human-wt/ZAF/MRC-DPRU1840/2007/G1P[8]
LC374134.1 RVA/Human-wt/NPL/09N3140/2009/G12P[6]
KF636154.1 RVA/Human-wt/ZAF/MRC-DPRU2325/2009/G1P[8]
MT737672.1/RVA/Human-wt/MOZ/MAN0033/2012/G1P[8]
KU738579.1 RVA/Human-wt/IND/S1/NIV0954435/2009/G9P[8]
HM773596.1 RVA/Human-wt/USA2009727036/2009/G9P[8]
EF990711.1 RVA/Human-wt/BEL/B3458/2003/G9P[8]
KP752601.1 RVA/Human-wt/ZAF/MRC-DPRU4799/2004/G1P[8]
KT920835.1 RVA/Human-wt/IND/VR10250/2003/G1P[8]
GU199511.1 RVA/Human-wt/BGD/Matlab36/2002/G11P[8]
DQ492677.1 RVA/Human-wt/BGD/Dhaka16/2003/G1P[8]
MG181235.1 RVA/Human-wt/MWI/MW1-337/1998/G1P[8]
KF371912.1 RVA/Human-wt/CHN/L1066/2009/G3P[8]
KT988160.1 RVA/Human-wt/ITA/PR3602/2005/G3P[8]
JN706630.1 RVA/Human-wt/THA/CU460-KK/09/2009/G12P[8]
MF580901.1 RVA/Human-wt/CHN/JS2015/2015/GxPx
KJ753465.1 RVA/Human-wt/ZWE/MRC-DPRU1102/2012/G9P[8]
EF672621.1 RVA/Human-tc/USA/WI61/1983/G9P1A[8]
MT737684.1/RVA/Human-wt/MOZ/HGM1789/2017/G1P[8]
MT737685.1/RVA/Human-wt/MOZ/HCB1682/2017/G1P[8]
MT737691.1/RVA/Human-wt/MOZ/HJM0338/2015/G1P[8]
RVA/Human-wt/MOZ/HGJM0408/2015/G1P[8]
MT737683.1/RVA/Human-wt/MOZ/HPQ1706/2017/G1P[8]
MT737682.1/RVA/Human-wt/MOZ/HCN1658/2017/G1P[8]
RVA/Human-wt/MOZ/HCN1556/2017/G1P[8]
MT737676.1/RVA/Human-wt/MOZ/HCN1624/2017/G1P[8]
KP752739.1 RVA/Human-wt/ZAF/MRC-DPRU1262/2004/G1P[8]
JX406753.1 RVA/Human-tc/USA/Wa/1974/G1P[8]
KX954622.1 RVA/Vaccine/USA/Rotarix-A41CB052A/1988/G1P1A[8]
JX943606.2 RVA/Human-TC/USA/Rotarix/2009/G1P[8]
LC173785.1 RVA/Human/JPN/NT053/2013/G1P[8]
KX632306.1 RVA/Human-wt/UGA/MUL-12-147/2012/G9P[8]
JN605456.1 RVA/Human-wt/KEN/MRC-DPRU2427/2010/G9P[8]
KJ626902.1 RVA/Human-wt/PRY/467/2000/G9P[8]
KX778510.1 RVA/Human-wt/CHN/km15105/G9P[8]
KU714441.1 RVA/Human-wt/MWI/MW2-181 A/2000/G1P[8]
RVA/Human-wt/MOZ/HGJM0644/2015/G9P[8]
DQ146657.1 RVA/Human-wt/BGD/Dhaka25/2002/G12P[8]
JX027928.1 RVA/Human-wt/AUS/CK00095/2010/G1P[8]
RVA/Human-wt/MOZ/HGJM0334/2015/G9P[8]
RVA/Human-wt/MOZ/HCN0370/2015/G9P[8]
RVA/Human-wt/MOZ/HGJM0407/2015/G9P[8]
RVA/Human-wt/MOZ/HGJM0385/2015/G9P[8]
RVA/Human-wt/MOZ/HGJM0413/2015/G9P[8]
RVA/Human-wt/MOZ/HGM0483/2015/G9P[8]
LC173940.1 RVA/Human/JPN/YM051/2013/G9P[8]
LC173912.1 RVA/Human/JPN/SP024/2013/G1P[8]
KT920681.1 RVA/Human-wt/USA/CNMC125/2011/G1P[8]
LC173926.1 RVA/Human-wt/JPN/YM017/2013/G9P[8]
RVA/Human-wt/MOZ/HGJM0318/2015/G9P[8]
RVA/Human-wt/MOZ/HGM0355/2015/G9P[8]
RVA/Human-wt/MOZ/HGM0322/2015/G9P[8]
RVA/Human-wt/MOZ/HGM0353/2015/G9P[8]
RVA/Human-wt/MOZ/HGJM0497/2015/G9P[8]
RVA/Human-wt/MOZ/HGM0389/2015/G9P[8]
RVA/Human-wt/MOZ/HGJM0643/2015/G9P[8]
HQ650122.1 RVA/Human-tc/USA/DS-1/1976/G2P[4]
0,050

NSP4-E1

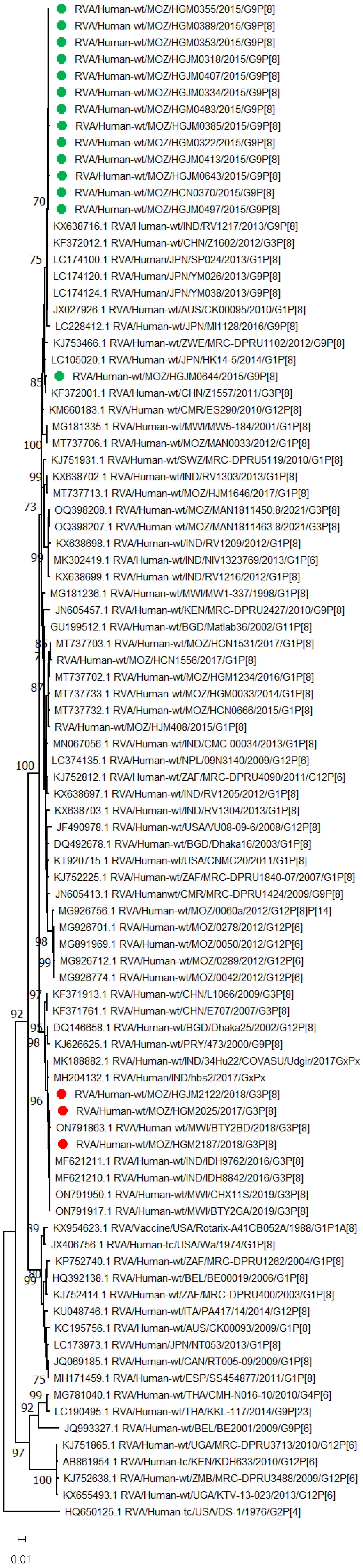
RVA/Human-wt/MOZ/HGM0355/2015/G9P[8]
RVA/Human-wt/MOZ/HGM0389/2015/G9P[8]
RVA/Human-wt/MOZ/HGM0353/2015/G9P[8]
RVA/Human-wt/MOZ/HGJM0318/2015/G9P[8]
RVA/Human-wt/MOZ/HGJM0407/2015/G9P[8]
RVA/Human-wt/MOZ/HGJM0334/2015/G9P[8]
RVA/Human-wt/MOZ/HGM0483/2015/G9P[8]
RVA/Human-wt/MOZ/HGJM0385/2015/G9P[8]
RVA/Human-wt/MOZ/HGM0322/2015/G9P[8]
RVA/Human-wt/MOZ/HGJM0413/2015/G9P[8]
RVA/Human-wt/MOZ/HGJM0643/2015/G9P[8]
RVA/Human-wt/MOZ/HCN0370/2015/G9P[8]
RVA/Human-wt/MOZ/HGJM0497/2015/G9P[8]
KX638716.1 RVA/Human-wt/IND/RV1217/2013/G9P[8]
KF372012.1 RVA/Human-wt/CHN/Z1602/2012/G3P[8]
LC174100.1 RVA/Human/JPN/SP024/2013/G1P[8]
LC174120.1 RVA/Human/JPN/YM026/2013/G9P[8]
LC174124.1 RVA/Human/JPN/YM038/2013/G9P[8]
JX027926.1 RVA/Human-wt/AUS/CK00095/2010/G1P[8]
LC228412.1 RVA/Human-wt/JPN/MI1128/2016/G9P[8]
KJ753466.1 RVA/Human-wt/ZWE/MRC-DPRU1102/2012/G9P[8]
LC105020.1 RVA/Human-wt/JPN/HK14-5/2014/G1P[8]
RVA/Human-wt/MOZ/HGJM0644/2015/G9P[8]
KF372001.1 RVA/Human-wt/CHN/Z1557/2011/G3P[8]
KM660183.1 RVA/Human-wt/CMR/ES290/2010/G12P[8]
MG181335.1 RVA/Human-wt/MWI/MW5-184/2001/G1P[8]
MT737706.1 RVA/Human-wt/MOZ/MAN0033/2012/G1P[8]
KJ751931.1 RVA/Human-wt/SWZ/MRC-DPRU5119/2010/G1P[8]
KX638702.1 RVA/Human-wt/IND/RV1303/2013/G1P[8]
MT737713.1 RVA/Human-wt/MOZ/HJM1646/2017/G1P[8]
OQ398208.1 RVA/Human-wt/MOZ/MAN1811450.8/2021/G3P[8]
OQ398207.1 RVA/Human-wt/MOZ/MAN1811463.8/2021/G3P[8]
KX638698.1 RVA/Human-wt/IND/RV1209/2012/G1P[8]
MK302419.1 RVA/Human-wt/IND/NIV1323769/2013/G1P[6]
KX638699.1 RVA/Human-wt/IND/RV1216/2012/G1P[8]
MG181236.1 RVA/Human-wt/MWI/MW1-337/1998/G1P[8]
JN605457.1 RVA/Human-wt/KEN/MRC-DPRU2427/2010/G9P[8]
GU199512.1 RVA/Human-wt/BGD/Matlab36/2002/G11P[8]
MT737703.1 RVA/Human-wt/MOZ/HCN1531/2017/G1P[8]
RVA/Human-wt/MOZ/HCN1556/2017/G1P[8]
MT737702.1 RVA/Human-wt/MOZ/HGM1234/2016/G1P[8]
MT737733.1 RVA/Human-wt/MOZ/HGM0033/2014/G1P[8]
MT737732.1 RVA/Human-wt/MOZ/HCN0666/2015/G1P[8]
RVA/Human-wt/MOZ/HJM408/2015/G1P[8]
MN067056.1 RVA/Human-wt/IND/CMC 00034/2013/G1P[8]
LC374135.1 RVA/Human-wt/NPL/09N3140/2009/G12P[6]
KJ752812.1 RVA/Human-wt/ZAF/MRC-DPRU4090/2011/G12P[6]
KX638697.1 RVA/Human-wt/IND/RV1205/2012/G1P[8]
KX638703.1 RVA/Human-wt/IND/RV1304/2013/G1P[8]
JF490978.1 RVA/Human-wt/USA/VU08-09-6/2008/G12P[8]
DQ492678.1 RVA/Human-wt/BGD/Dhaka16/2003/G1P[8]
KT920715.1 RVA/Human-wt/USA/CNMC20/2011/G1P[8]
KJ752225.1 RVA/Human-wt/ZAF/MRC-DPRU1840-07/2007/G1P[8]
JN605413.1 RVA/Humanwt/CMR/MRC-DPRU1424/2009/G9P[8]
MG926756.1 RVA/Human-wt/MOZ/0060a/2012/G12P[8]P[14]
MG926701.1 RVA/Human-wt/MOZ/0278/2012/G12P[6]
MG891969.1 RVA/Human-wt/MOZ/0050/2012/G12P[6]
MG926712.1 RVA/Human-wt/MOZ/0289/2012/G12P[6]
MG926774.1 RVA/Human-wt/MOZ/0042/2012/G12P[6]
KF371913.1 RVA/Human-wt/CHN/L1066/2009/G3P[8]
KF371761.1 RVA/Human-wt/CHN/E707/2007/G3P[8]
DQ146658.1 RVA/Human-wt/BGD/Dhaka25/2002/G12P[8]
KJ626625.1 RVA/Human-wt/PRY/473/2000/G9P[8]
MK188882.1 RVA/Human-wt/IND/34Hu22/COVASU/Udgir/2017GxPx
MH204132.1 RVA/Human/IND/hbs2/2017/GxPx
RVA/Human-wt/MOZ/HGJM2122/2018/G3P[8]
RVA/Human-wt/MOZ/HGM2025/2017/G3P[8]
ON791863.1 RVA/Human-wt/MWI/BTY2BD/2018/G3P[8]
RVA/Human-wt/MOZ/HGM2187/2018/G3P[8]
MF621211.1 RVA/Human-wt/IND/IDH9762/2016/G3P[8]
MF621210.1 RVA/Human-wt/IND/IDH8842/2016/G3P[8]
ON791950.1 RVA/Human-wt/MWI/CHX11S/2019/G3P[8]
ON791917.1 RVA/Human-wt/MWI/BTY2GA/2019/G3P[8]
KX954623.1 RVA/Vaccine/USA/Rotarix-A41CB052A/1988/G1P1A[8]
JX406756.1 RVA/Human-tc/USA/Wa/1974/G1P[8]
KP752740.1 RVA/Human-wt/ZAF/MRC-DPRU1262/2004/G1P[8]
HQ392138.1 RVA/Human-wt/BEL/BE00019/2006/G1P[8]
KJ752414.1 RVA/Human-wt/ZAF/MRC-DPRU400/2003/G1P[8]
KU048746.1 RVA/Human-wt/ITA/PA417/14/2014/G12P[8]
KC195756.1 RVA/Human-wt/AUS/CK00093/2009/G1P[8]
LC173973.1 RVA/Human/JPN/NT053/2013/G1P[8]
JQ069185.1 RVA/Human-wt/CAN/RT005-09/2009/G1P[8]
MH171459.1 RVA/Human-wt/ESP/SS454877/2011/G1P[8]
MG781040.1 RVA/Human-wt/THA/CMH-N016-10/2010/G4P[6]
LC190495.1 RVA/Human-wt/THA/KKL-117/2014/G9P[23]
JQ993327.1 RVA/Human-wt/BEL/BE2001/2009/G9P[6]
KJ751865.1 RVA/Human-wt/UGA/MRC-DPRU3713/2010/G12P[6]
AB861954.1 RVA/Human-tc/KEN/KDH633/2010/G12P[6]
KJ752638.1 RVA/Human-wt/ZMB/MRC-DPRU3488/2009/G12P[6]
KX655493.1 RVA/Human-wt/UGA/KTV-13-023/2013/G12P[6]
HQ650125.1 RVA/Human-tc/USA/DS-1/1976/G2P[4]
0,01

NSP5/6-H1

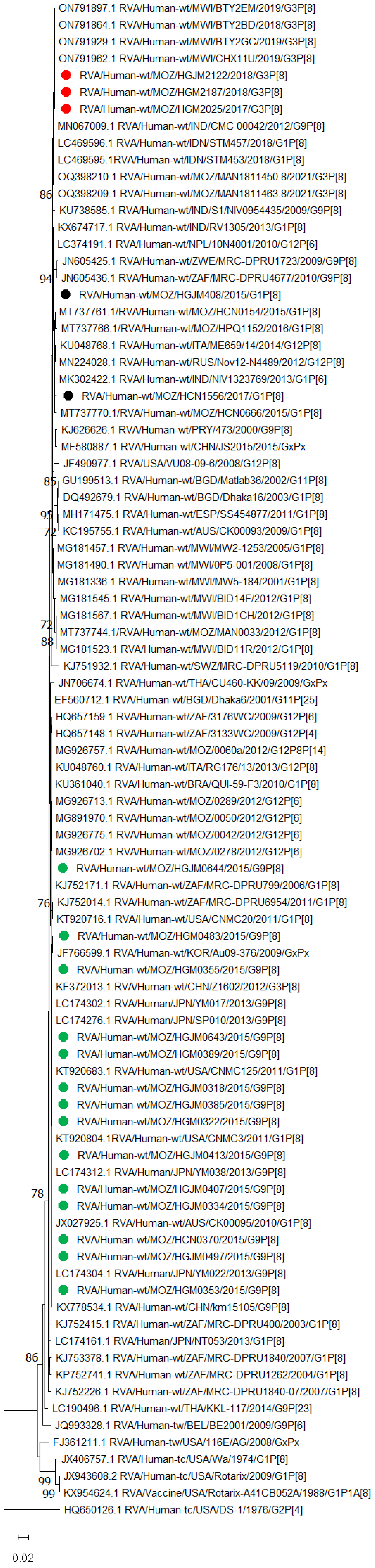
ON791897.1 RVA/Human-wt/MWI/BTY2EM/2019/G3P[8]
ON791864.1 RVA/Human-wt/MWI/BTY2BD/2018/G3P[8]
ON791929.1 RVA/Human-wt/MWI/BTY2GC/2019/G3P[8]
ON791962.1 RVA/Human-wt/MWI/CHX11U/2019/G3P[8]
RVA/Human-wt/MOZ/HGJM2122/2018/G3P[8]
RVA/Human-wt/MOZ/HGM2187/2018/G3P[8]
RVA/Human-wt/MOZ/HGM2025/2017/G3P[8]
MN067009.1 RVA/Human-wt/IND/CMC 00042/2012/G9P[8]
LC469596.1 RVA/Human-wt/IDN/STM457/2018/G1P[8]
LC469595.1RVA/Human-wt/IDN/STM453/2018/G1P[8]
OQ398210.1 RVA/Human-wt/MOZ/MAN1811450.8/2021/G3P[8]
86
OQ398209.1 RVA/Human-wt/MOZ/MAN1811463.8/2021/G3P[8]
KU738585.1 RVA/Human-wt/IND/S1/NIV0954435/2009/G9P[8]
KX674717.1 RVA/Human-wt/IND/RV1305/2013/G1P[8]
LC374191.1 RVA/Human-wt/NPL/10N4001/2010/G12P[6]
JN605425.1 RVA/Human-wt/ZWE/MRC-DPRU1723/2009/G9P[8]
94
JN605436.1 RVA/Human-wt/ZAF/MRC-DPRU4677/2010/G9P[8]
RVA/Human-wt/MOZ/HGJM408/2015/G1P[8]
MT737761.1/RVA/Human-wt/MOZ/HCN0154/2015/G1P[8]
MT737766.1/RVA/Human-wt/MOZ/HPQ1152/2016/G1P[8]
KU048768.1 RVA/Human-wt/ITA/ME659/14/2014/G12P[8]
MN224028.1 RVA/Human-wt/RUS/Nov12-N4489/2012/G12P[8]
MK302422.1 RVA/Human-wt/IND/NIV1323769/2013/G1P[6]
RVA/Human-wt/MOZ/HCN1556/2017/G1P[8]
MT737770.1/RVA/Human-wt/MOZ/HCN0666/2015/G1P[8]
KJ626626.1 RVA/Human-wt/PRY/473/2000/G9P[8]
MF580887.1 RVA/Human-wt/CHN/JS2015/2015/GxPx
JF490977.1 RVA/USA/VU08-09-6/2008/G12P[8]
85
GU199513.1 RVA/Human-wt/BGD/Matlab36/2002/G11P[8]
DQ492679.1 RVA/Human-wt/BGD/Dhaka16/2003/G1P[8]
95
MH171475.1 RVA/Human-wt/ESP/SS454877/2011/G1P[8]
72
KC195755.1 RVA/Human-wt/AUS/CK00093/2009/G1P[8]
MG181457.1 RVA/Human-wt/MWI/MW2-1253/2005/G1P[8]
MG181490.1 RVA/Human-wt/MWI/0P5-001/2008/G1P[8]
MG181336.1 RVA/Human-wt/MWI/MW5-184/2001/G1P[8]
MG181545.1 RVA/Human-wt/MWI/BID14F/2012/G1P[8]
MG181567.1 RVA/Human-wt/MWI/BID1CH/2012/G1P[8]
72
MT737744.1/RVA/Human-wt/MOZ/MAN0033/2012/G1P[8]
88
MG181523.1 RVA/Human-wt/MWI/BID11R/2012/G1P[8]
KJ751932.1 RVA/Human-wt/SWZ/MRC-DPRU5119/2010/G1P[8]
JN706674.1 RVA/Human-wt/THA/CU460-KK/09/2009/GxPx
EF560712.1 RVA/Human-wt/BGD/Dhaka6/2001/G11P[25]
HQ657159.1 RVA/Human-wt/ZAF/3176WC/2009/G12P[6]
HQ657148.1 RVA/Human-wt/ZAF/3133WC/2009/G12P[4]
MG926757.1 RVA/Human-wt/MOZ/0060a/2012/G12P8P[14]
KU048760.1 RVA/Human-wt/ITA/RG176/13/2013/G12P[8]
KU361040.1 RVA/Human-wt/BRA/QUI-59-F3/2010/G1P[8]
MG926713.1 RVA/Human-wt/MOZ/0289/2012/G12P[6]
MG891970.1 RVA/Human-wt/MOZ/0050/2012/G12P[6]
MG926775.1 RVA/Human-wt/MOZ/0042/2012/G12P[6]
MG926702.1 RVA/Human-wt/MOZ/0278/2012/G12P[6]
RVA/Human-wt/MOZ/HGJM0644/2015/G9P[8]
KJ752171.1 RVA/Human-wt/ZAF/MRC-DPRU799/2006/G1P[8]
76
KJ752014.1 RVA/Human-wt/ZAF/MRC-DPRU6954/2011/G1P[8]
KT920716.1 RVA/Human-wt/USA/CNMC20/2011/G1P[8]
RVA/Human-wt/MOZ/HGM0483/2015/G9P[8]
JF766599.1 RVA/Human-wt/KOR/Au09-376/2009/GxPx
RVA/Human-wt/MOZ/HGM0355/2015/G9P[8]
KF372013.1 RVA/Human-wt/CHN/Z1602/2012/G3P[8]
LC174302.1 RVA/Human/JPN/YM017/2013/G9P[8]
LC174276.1 RVA/Human/JPN/SP010/2013/G9P[8]
RVA/Human-wt/MOZ/HGJM0643/2015/G9P[8]
RVA/Human-wt/MOZ/HGM0389/2015/G9P[8]
KT920683.1 RVA/Human-wt/USA/CNMC125/2011/G1P[8]
RVA/Human-wt/MOZ/HGJM0318/2015/G9P[8]
RVA/Human-wt/MOZ/HGJM0385/2015/G9P[8]
RVA/Human-wt/MOZ/HGM0322/2015/G9P[8]
KT920804.1RVA/Human-wt/USA/CNMC3/2011/G1P[8]
RVA/Human-wt/MOZ/HGJM0413/2015/G9P[8]
LC174312.1 RVA/Human/JPN/YM038/2013/G9P[8]
78
RVA/Human-wt/MOZ/HGJM0407/2015/G9P[8]
RVA/Human-wt/MOZ/HGJM0334/2015/G9P[8]
JX027925.1 RVA/Human-wt/AUS/CK00095/2010/G1P[8]
RVA/Human-wt/MOZ/HCN0370/2015/G9P[8]
RVA/Human-wt/MOZ/HGJM0497/2015/G9P[8]
LC174304.1 RVA/Human/JPN/YM022/2013/G9P[8]
RVA/Human-wt/MOZ/HGM0353/2015/G9P[8]
KX778534.1 RVA/Human-wt/CHN/km15105/G9P[8]
KJ752415.1 RVA/Human-wt/ZAF/MRC-DPRU400/2003/G1P[8]
LC174161.1 RVA/Human/JPN/NT053/2013/G1P[8]
86
KJ753378.1 RVA/Human-wt/ZAF/MRC-DPRU1840/2007/G1P[8]
KP752741.1 RVA/Human-wt/ZAF/MRC-DPRU1262/2004/G1P[8]
KJ752226.1 RVA/Human-wt/ZAF/MRC-DPRU1840-07/2007/G1P[8]
LC190496.1 RVA/Human-wt/THA/KKL-117/2014/G9P[23]
JQ993328.1 RVA/Human-tw/BEL/BE2001/2009/G9P[8]
FJ361211.1 RVA/Human-tw/USA/116E/AG/2008/GxPx
JX406757.1 RVA/Human-tc/USA/Wa/1974/G1P[8]
99
JX943608.2 RVA/Human-tc/USA/Rotarix/2009/G1P[8]
99
KX954624.1 RVA/Vaccine/USA/Rotarix-A41CB052A/1988/G1P1A[8]
HQ650126.1 RVA/Human-tc/USA/DS-1/1976/G2P[4]
0.02
