# Supplementary material for: Whole-Genome Characterization of Rotavirus G9P[6] and G9P[4] Strains That Emerged after Rotavirus Vaccine Introduction in Mozambique
Source: Viruses. 2024 Jul 16;16(7):1140. doi: 10.3390/v16071140 (PMC11281483; doi:10.3390/v16071140)
Supplement: Supplementary file 1 [file viruses-16-01140-s001.zip › Table S1.pdf]

Supplementary Table S1: Vaccination status of the children infected by G2P[6], G2P[6], G3P[4], G3P[8] and G1P[8] Mozambican strains.

Strain	Age by months	Vaccination_status	Province	Year
RVA/Human-wt/MOZ/HGJM0318/2015/G9P[8]	0-11	NA	Maputo	2015
RVA/Human-wt/MOZ/HGM0322/2015/G9P[8]	12-23	NA	Maputo	2015
RVA/Human-wt/MOZ/HGJM0334/2015/G9P[8]	24-59	NA	Maputo	2015
RVA/Human-wt/MOZ/HGM0353/2015/G9P[8]	12-23	NA	Maputo	2015
RVA/Human-wt/MOZ/HGM0355/2015/G9P[8]	0-11	NA	Maputo	2015
RVA/Human-wt/MOZ/HCN0370/2015/G9P[8]	0-11	NA	Nampula	2015
RVA/Human-wt/MOZ/HGJM0385/2015/G9P[8]	12-23	NA	Maputo	2015
RVA/Human-wt/MOZ/HGM0389/2015/G9P[8]	12-23	NA	Maputo	2015
RVA/Human-wt/MOZ/HGJM0407/2015/G9P[8]	12-23	NA	Maputo	2015
RVA/Human-wt/MOZ/HGJM0408/2015/G1P[8]	12-23	NA	Maputo	2015
RVA/Human-wt/MOZ/HGJM0413/2015/G9P[8]	0-11	NA	Maputo	2015
RVA/Human-wt/MOZ/HGM483/2015/G9P[8]	0-11	NA	Maputo	2015
RVA/Human-wt/MOZ/HGJM0492/2015/G2P[4]	0-11	NA	Maputo	2015
RVA/Human-wt/MOZ/HGJM0497/2015/G9P[8]	0-11	NA	Maputo	2015
RVA/Human-wt/MOZ/HGQ0561/2015/G2P[6]	0-11	NA	Zambézia	2015
RVA/Human-wt/MOZ/HGJM0643/2015/G9P[8]	0-11	NA	Maputo	2015
RVA/Human-wt/MOZ/HGJM0644/2015/G9P[8]	0-11	NA	Maputo	2015
RVA/Human-wt/MOZ/HCN0738/2015/G2P[6]	0-11	NA	Nampula	2015
RVA/Human-wt/MOZ/HGQ1104/2016/G2P[6]	12-23	NV	Zambézia	2016
RVA/Human-wt/MOZ/HGQ1141/2016/G2P[4]	12-23	NV	Zambézia	2016
RVA/Human-wt/MOZ/HPQ1208/2016/G2P[6]	12-23	First dose	Zambézia	2016
RVA/Human-wt/MOZ/HGM1235/2016/G3P[4]	24-59	NV	Maputo	2016
RVA/Human-wt/MOZ/HCN1284/2016/G2P[6]	0-11	NV	Nampula	2016

RVA/Human-wt/MOZ/HGQ1296/2016/G9P[6]	0-11	NV	Zambezia	2016
RVA/Human-wt/MOZ/HCN1313/2016/G2P[6]	0-11	Second dose	Nampula	2016
RVA/Human-wt/MOZ/HCN1328/2016/G2P[6]	12-23	Second dose	Nampula	2016
RVA/Human-wt/MOZ/HCN1347/2016/G9P[6]	0-11	NV	Nampula	2016
RVA/Human-wt/MOZ/HCN1357/2016/G9P[6]	0-11	NV	Nampula	2016
RVA/Human-wt/MOZ/HCN1359/2016/G9P[6]	12-23	NV	Nampula	2016
RVA/Human-wt/MOZ/HCN1369/2017/G9P[6]	0-11	Second dose	Nampula	2017
RVA/Human-wt/MOZ/HCN1371/2017/G9P[6]	12-23	NV	Nampula	2017
RVA/Human-wt/MOZ/HCN1556/2017/G1P[8]	12-23	Second dose	Nampula	2017
RVA/Human-wt/MOZ/HCN1595/2017/G9P[4]	0-11	Second dose	Nampula	2017
RVA/Human-wt/MOZ/HCN1597/2017/G9P[6]	0-11	NV	Nampula	2017
RVA/Human-wt/MOZ/HCN1598/2017/G9P[4]	0-11	NV	Nampula	2017
RVA/Human-wt/MOZ/HCN1600/2017/G9P[4]	0-11	First dose	Nampula	2017
RVA/Human-wt/MOZ/HCN1604/2017/G9P[4]	0-11	Second dose	Nampula	2017
RVA/Human-wt/MOZ/HGJM1647/2017/G9P[4]	12-23	Second dose	Maputo	2017
RVA/Human-wt/MOZ/HGJM1696/2017/G3P[4]	24-59	NV	Maputo	2017
RVA/Human-wt/MOZ/HGJM1768/2017/G3P[4]	24-59	NV	Maputo	2017
RVA/Human-wt/MOZ/HGJM1782/2017/G9P[6]	12-23	Second dose	Maputo	2017
RVA/Human-wt/MOZ/HCN1855/2017/G9P[4]	0-11	Second dose	Nampula	2018
RVA/Human-wt/MOZ/HGJM1856/2018/G3P[4]	12-23	Second dose	Maputo	2018
RVA/Human-wt/MOZ/HGM1883/2018/G9P[6]	0-11	NV	Maputo	2018
RVA/Human-wt/MOZ/HGM2025/2017/G3P[8]	12-23	Second dose	Maputo	2017
RVA/Human-wt/MOZ/HGJM2122/2018/G3P[8]	0-11	Second dose	Maputo	2018
RVA/Human-wt/MOZ/HGM2187/2018/G3P[8]	12-23	Second dose	Maputo	2018

NV- not vaccinated
NA- not applicable. Refers to the pre-vaccine period.
