# Supplementary material for: Whole-Genome Characterization of Rotavirus G9P[6] and G9P[4] Strains That Emerged after Rotavirus Vaccine Introduction in Mozambique
Source: Viruses. 2024 Jul 16;16(7):1140. doi: 10.3390/v16071140 (PMC11281483; doi:10.3390/v16071140)
Supplement: Supplementary file 1 [file viruses-16-01140-s001.zip › Table S2.pdf]

Supplementary Table S2: Genotype constellation of Mozambican G2P[6], G2P[6], G3P[4], G3P[8] and G1P[8]strains with DS-1 like and Wa-like constellations.

**Strain name**	**VP7**	**VP4**	**VP6**	**VP1**	**VP2**	**VP3**	**NSP1**	**NSP2**	**NSP3**	**NSP4**	**NSP5/6**
**Segment**	**9**	**4**	**6**	**1**	**2**	**3**	**5**	**8**	**7**	**10**	**11**
**DS1**	G2	P[4]	I2	R2	C2	M2	A2	N2	T2	E2	H2
**Wa**	G1	P[8]	I1	R1	C2	M1	A1	N1	T1	E1	H1
RVA/Human-wt/MOZ/HGQ0561/2015/G2P[6]	G2	P[6]	I2	R2	C2	M2	A2	N2	T2	E2	H2
RVA/Human-wt/MOZ/HCN0738/2015/G2P[6]	G2	P[6]	I2	R2	C2	M2	A2	N2	T2	E2	H2
RVA/Human-wt/MOZ/HGQ1104/2016/G2P[6]	G2	P[6]	I2	R2	C2	M2	A2	N2	T2	E2	H2
RVA/Human-wt/MOZ/HGQ1208/2016/G2P[6]	G2	P[6]	I2	R2	C2	M2	A2	N2	T2	E2	H2
RVA/Human-wt/MOZ/HCN1284/2016/G2P[6]	G2	P[6]	I2	R2	C2	M2	A2	N2	T2	E2	H2
RVA/Human-wt/MOZ/HCN1313/2016/G2P[6]	G2	P[6]	I2	R2	C2	M2	A2	N2	T2	E2	H2
RVA/Human-wt/MOZ/HCN1328/2016/G2P[6]	G2	P[6]	I2	R2	C2	M2	A2	N2	T2	E2	H2
RVA/Human-wt/MOZ/HGJM0492/2015/G2P[4]	G2	P[4]	I2	R2	C2	M2	A2	N2	T2	E2	H2
RVA/Human-wt/MOZ/HGQ1141/2016/G2P[4]	G2	P[4]	I2	R2	C2	M2	A2	N2	T2	E2	H2
RVA/Human-wt/MOZ/HGJM1856/2018/G3P[4]	G3	P[4]	I2	R2	C2	M2	A2	N2	T2	E2	H2
RVA/Human-wt/MOZ/HGJM1768/2017/G3P[4]	G3	P[4]	I2	R2	C2	M2	A2	N2	T2	E2	H2
RVA/Human-wt/MOZ/HGJM1696/2017/G3P[4]	G3	P[4]	I2	R2	C2	M2	A2	N2	T2	E2	H2
RVA/Human-wt/MOZ/HGM1235/2016/G3P[4]	G3	P[4]	I2	R2	C2	M2	A2	N2	T2	E2	H2
RVA/Human-wt/MOZ/HGM2187/2018/G3P[8]	G3	P[8]	I1	R1	C2	M1	A1	N1	T1	E1	H1
RVA/Human-wt/MOZ/HGJM2122/2018/G3P[8]	G3	P[8]	I1	R1	C2	M1	A1	N1	T1	E1	H1
RVA/Human-wt/MOZ/HGM2025/2017/G3P[8]	G3	P[8]	I1	R1	C2	M1	A1	N1	T1	E1	H1
RVA/Human-wt/MOZ/HCN1556/2017/G1P[8]	G1	P[8]	I1	R1	C2	M1	A1	N1	T1	E1	H1
RVA/Human-wt/MOZ/HGJM0408/2015/G1P[8]	G1	P[8]	I1	R1	C2	M1	A1	N1	T1	E1	H1
